# 360-Degree Perspectives on Obesity

**DOI:** 10.3390/medicina59061119

**Published:** 2023-06-09

**Authors:** Magdalena Cuciureanu, Cătălin-Cezar Caratașu, Levon Gabrielian, Otilia Elena Frăsinariu, Laura Elisabeta Checheriță, Laura Mihaela Trandafir, Gabriela Dumitrița Stanciu, Andrei Szilagyi, Ina Pogonea, Gabriela Bordeianu, Radu Petru Soroceanu, Călin Vasile Andrițoiu, Maria Mihalache Anghel, Diana Munteanu, Irina Teodora Cernescu, Bogdan Ionel Tamba

**Affiliations:** 1 Department of Pharmacology, “Grigore T. Popa” University of Medicine and Pharmacy, 700115 Iasi, Romania; mag.cuciureanu@umfiasi.ro (M.C.); caratasu.catalin@umfiasi.ro (C.-C.C.); teodora.cernescu@umfiasi.ro (I.T.C.); bogdan.tamba@umfiasi.ro (B.I.T.); 2 Center for Advanced Research and Development in Experimental Medicine (CEMEX), “Grigore T. Popa” University of Medicine and Pharmacy, 700115 Iasi, Romania; gabriela-dumitrita.stanciu@umfiasi.ro (G.D.S.); andrei.szilagyi@umfiasi.ro (A.S.); 3 Department of Anatomy and Pathology, The University of Adelaide, Adelaide 5000, Australia; levon.gabrielian@adelaide.edu.au; 4 Department of Mother and Child, “Grigore T. Popa” University of Medicine and Pharmacy, 700115 Iasi, Romania; laura.trandafir@umfiasi.ro; 5 2nd Dental Medicine Department, “Grigore T. Popa” University of Medicine and Pharmacy, 700115 Iasi, Romania; 6 Department of Pharmacology and Clinical Pharmacology, “Nicolae Testemiţanu” State University of Medicine and Pharmacy, 2004 Chisinau, Moldova; ina.pogonea@usmf.md (I.P.); maria.mihalachi-anghel@usmf.md (M.M.A.); 7 Department of Biochemistry, “Grigore T. Popa” University of Medicine and Pharmacy, 700115 Iasi, Romania; gabriela.bordeianu@umfiasi.ro; 8 Department of Surgery, “Grigore T. Popa” University of Medicine and Pharmacy, 700115 Iasi, Romania; radu-petru.soroceanu@umfiasi.ro; 9 Specialization of Nutrition and Dietetics, “Vasile Goldis” Western University of Arad, 310025 Arad, Romania; 10 Institute of Mother and Child, “Nicolae Testemiţanu” State University of Medicine and Pharmacy, 2062 Chisinau, Moldova; diana.munteanu@usmf.md

**Keywords:** obesity, stress, PTSD, epigenetics, microbiota, genetics, pharmacotherapy, bariatric surgery

## Abstract

Alarming statistics show that the number of people affected by excessive weight has surpassed 2 billion, representing approximately 30% of the world’s population. The aim of this review is to provide a comprehensive overview of one of the most serious public health problems, considering that obesity requires an integrative approach that takes into account its complex etiology, including genetic, environmental, and lifestyle factors. Only an understanding of the connections between the many contributors to obesity and the synergy between treatment interventions can ensure satisfactory outcomes in reducing obesity. Mechanisms such as oxidative stress, chronic inflammation, and dysbiosis play a crucial role in the pathogenesis of obesity and its associated complications. Compounding factors such as the deleterious effects of stress, the novel challenge posed by the obesogenic digital (food) environment, and the stigma associated with obesity should not be overlooked. Preclinical research in animal models has been instrumental in elucidating these mechanisms, and translation into clinical practice has provided promising therapeutic options, including epigenetic approaches, pharmacotherapy, and bariatric surgery. However, more studies are necessary to discover new compounds that target key metabolic pathways, innovative ways to deliver the drugs, the optimal combinations of lifestyle interventions with allopathic treatments, and, last but not least, emerging biological markers for effective monitoring. With each passing day, the obesity crisis tightens its grip, threatening not only individual lives but also burdening healthcare systems and societies at large. It is high time we took action as we confront the urgent imperative to address this escalating global health challenge head-on.

## 1. Introduction

The World Health Organization defines obesity as an “abnormal or excessive accumulation of fat that poses a risk to health” [1,2]. However, can the medical community continue to treat obesity solely as a risk factor? This has been an unanswered question for more than 50 years, during which time obesity has continued to rise rapidly to pandemic proportions. Today, with an estimated 650 million adults and 124 million children affected in 2016, obesity is recognized as a complex, chronic, and multifactorial disease. The economic impact of obesity is substantial, with obesity-related healthcare costs alone projected to exceed USD 190 billion annually in the United States [2]. In addition, obesity is associated with reduced productivity, increased absenteeism, and reduced work capacity, resulting in significant economic losses at both the individual and societal levels [2,3].

The causes of obesity are multifaceted, including genetic, epigenetic, psycho-social, and microenvironmental factors. We are now gaining a better understanding of the upregulation of food cravings in the brain of obese individuals, as well as the role of gut hormones, adipose tissue, and gut dysbiosis in regulating appetite and satiety in the hypothalamus [4].

Oxidative stress triggers inflammatory cascades, while inflammation increases oxidative stress through various pathways, creating a reciprocal relationship. Dysbiosis, in turn, contributes to oxidative stress and inflammation through microbial-derived metabolites, lipopolysaccharides, and altered gut permeability. These interactions form a triad of intertwined mechanisms that perpetuate the pathophysiology of obesity and its complications [5].

Embracing a holistic approach that addresses oxidative stress, chronic inflammation, and dysbiosis is pivotal in developing effective preventive strategies and comprehensive management approaches to tackle the global obesity epidemic.

Obesity has traditionally been viewed as a condition resulting from excessive storage of energy in fat cells due to an imbalance between energy intake and expenditure [6]. Current research, however, has shown that the sources and quality of nutrients in the diet may play a more important role than quantity in weight control and disease prevention [7,8]. These studies have suggested that alterations in the gut microbiota and branched-chain amino acid (BCAA) metabolism may play an important role in the development and progression of obesity [9,10]. The gut microbiota, a diverse community of microorganisms that inhabits the gastrointestinal tract, has been shown to regulate energy metabolism and immune function, among other physiological processes [11]. Meanwhile, BCAAs, a group of essential amino acids that cannot be synthesized by the body and must be obtained from the diet, have been implicated in the pathogenesis of obesity and related metabolic disorders [10,12].

While environmental factors such as diet and physical activity play an important role in the development of obesity, there is some increasing evidence that genetic factors also play a key role in determining an individual’s susceptibility to obesity. Polygenic forms of obesity are influenced by the interaction of several genetic factors and are more common than monogenic forms [8]. Understanding the genetic basis of obesity is important because it can provide valuable insights into the underlying mechanisms of the disease and help identify new targets for therapeutic intervention.

From the perspective of fundamental research and the translation of results from experimental medicine into clinical practice, we consider the use of animal models to be an important tool for understanding the pathophysiology of obesity and developing effective interventions for its prevention and treatment. Among animal models, rodents have been extensively used because of their genetic, physiological, and metabolic similarities to humans. In particular, rodent models of obesity have provided valuable insights into the genetic basis of the disease and the mechanisms underlying its development and progression. In recent years, preclinical research has focused on the relationship between oxidative stress and chronic inflammation in obesity [13,14]. In particular, oxidative stress has been identified as a primary trigger of the inflammatory response.

However, the psychogenic factor plays a decisive role in the development of obesity [15]. Research has shown that there is a strong association between stress and weight gain, with obesity being more common in people with PTSD [16]. Chronic inflammation, a common denominator in both obesity and PTSD, serves as a potential mechanistic link, impacting neural circuits involved in stress response, emotion regulation, and appetite control. Furthermore, the complex association between obesity, chronic inflammation, and mental disorders extends beyond PTSD, encompassing other psychiatric conditions such as depression and anxiety disorders [17,18]. Therefore, in this paper, we will provide a comprehensive review of the current literature on the relationship between obesity and PTSD, with a particular focus on the mechanisms of high cortisol levels and sympathetic overactivity.

Despite significant public health efforts, the prevalence of obesity continues to rise at an alarming rate. Behavioral and lifestyle changes remain the cornerstone of obesity management, but epigenetic interventions, pharmacotherapy, and bariatric surgery have also emerged as important tools in the fight against obesity. Pharmacotherapy involves the use of drugs to aid weight loss, mainly by reducing appetite, increasing satiety, or blocking the absorption of fat. Bariatric surgery involves surgical modification of the gastrointestinal tract to induce weight loss. Despite their potential benefits, both drug therapy and bariatric surgery have limitations and risks. Medications can cause side effects and drug interactions and may not be effective for all patients [19]. Bariatric surgery carries risks such as infection, bleeding, and malnutrition and requires significant lifestyle changes after surgery [20]. It is therefore important to carefully consider the risks and benefits of these interventions on a case-by-case basis.

To sum up, this comprehensive review explores the intricate relationship between obesity, the gut-brain axis, chronic inflammation, epigenetics, and mental disorders, addressing the interconnected nature of these factors, transcending traditional boundaries, and embracing a holistic perspective in tackling this global epidemic. By bridging the gap between disciplines, adopting innovative research methodologies, and fostering collaboration, we can forge a path toward effective preventive strategies and personalized therapeutic interventions.

## 2. Rodent Models of Obesity

Our understanding of obesity and its complex interactions is largely due to the use of animal models, a practice that dates back to ancient Greece. Certain parameters must be met for the selection of an animal model: pathophysiological similarities to human disease, phenotypic similarity to disease status, simplicity, replicability, reproducibility, and cost-effectiveness [21]. Two frequently-used research models in this particular medical field are mice and rats [22]. All models have pros and cons that need to be taken into consideration before deciding upon a specific one (Table 1).

### 2.1. Monogenic Models

Animals with a single gene abnormality are referred to as monogenetic animal models [22]. They are reliable and efficient tools that are frequently used to investigate obesity [22]. These models have a molecular map that is well-structured [22]. However, they differ from people in one important manner: how they perform their energy distribution and fat deposition [22]. Generally speaking, they do not accurately represent human disorders [22,23].

*Ob*/*ob* mice were brought to light in 1949 by researchers at the Jackson Laboratory [24]. The gene product for this mutation had not been named leptin until 1994 when it was genetically identified as a single base pair deletion [22,25,26,27]. Due to their early-onset obesity, which is characterized by high energy income and low energy output, these mice are frequently employed [22,24,28].

*Db*/*db* mice–both the *db*/*db* mouse (short for diabetes) and the *ob*/*ob* mouse model have an abnormality in the leptin receptor gene, causing impaired leptin signaling [22,23,24]. These mice are characterized by high energy input and low energy output, which triggers early-onset obesity, insulin resistance, lower-than-normal insulin levels, and hypothermia [22]. Additionally, they are infertile, and their growth is hampered by a lack of growth hormones [22,24,25,26,29].

*S*/*s mice*–in the s/s mouse model, the long signaling pathways through which leptin exerts its functions are impaired [23,27,30]. In contrast to the *ob*/*ob* or *db*/*db* models, the s/s mice have an abnormally high appetite, are fat, have a standard body size, and are fertile [22,26].

*B6 (cg)-Tubtub*/*J*–tubby (tub), an autosomal recessive mutation, occurred by chance in a C57BL/6J colony [22,31]. Tub expression in the hypothalamic arcuate, paraventricular, and ventromedial nuclei implies a function in controlling body weight or food behavior [22,32].

*Zucker Fatty Rat*–ZFR, or the Zucker (*fa*/*fa*), is the offspring of the cross between the 13 M strain of rats from Merck and Sherman and was developed by L. M. Zucker and T. F. Zucker in 1961 [22,33,34]. The ZFRs’ mutation of the leptin receptor makes them have a rather weak response to leptin, making them phenotypically close to *ob*/*ob* and *db*/*db* mice [22,29,35].

*Otsuka Long-Evans Tokushima Fatty rat*–OLETF rats are deficient in type A cholecystokinin (CCK) receptors, which contributes to their phenotype [36]. They represent an important means of observing unbalanced eating behavior, as CCK impairs satiety [22,26].

### 2.2. Polygenic Models

Seeing as human obesity is influenced by numerous genes, polygenic models, as opposed to monogenic models, offer greater data on the nature of obesity [22,25]. The following are a few of the most popular polygenic models [22].

*New Zealand obese mice* resemble the *ob*/*ob* strain in many ways. Type 2 diabetes and obesity exclusively occur in males [22,24,25,37].

*Tsumura and Suzuki obesity and diabetes mice*–polygenic obesity, insulin resistance, polydipsia, hyperglycemia, polyuria, and hyperinsulinemia are characteristics of male TSOD mice [22,38,39].

*Kuo Kondo-Ay mice*–KK-Ay mice have unique adiposity and are grossly overweight. At eight weeks of age, they show hyperphagia, hyperglycemia, hyperinsulinemia, and glucose intolerance [22,38,40].

*M16 mice*–the M16 mouse develops early-onset obesity and moderate hyperglycemia alongside hyperphagia, hyperinsulinemia, and hyperleptinemia [25,41,42].

### 2.3. Genetically Modified Mice

Mice that have been genetically altered are frequently employed in research to examine biological processes in vivo, modify diseases, and study genetic factors [22,43]. Mice are thought to be the mammals best suited for this task since they share human organ and tissue structures [22,43].

*Transgenic mice*–to simplify obtaining animals with hereditary traits that are exactly like those observed in people that are obese, these types of research models were developed [22,23].

- Corticotropin-releasing factor overexpressing mice.
- Melanin-concentrating hormone overexpressing (MCH-OE) mice.
- Overexpression of 11β-hydroxysteroid dehydrogenase Type 1 (11β HSD1) mice.
- Overexpression of glucose transported subtype 4.
- UCP-DTA Mice

*Knockout mice* (KO) are an important weapon in exploring prospective plans for the production of cures for certain diseases, including drug-based and genetic therapeutic approaches [22]. Another important purpose of these mouse models is to understand the function and importance of specific genes [22]. They are a great way to analyze the metabolic activities of particular genes and mimic human illnesses [22,44].

- Beta-3 adrenergic receptor knockout mice.
- Bombesin receptor subtype 3 knockout mice.
- Deletion of the neuronal insulin receptor (NIRKO) in mice.
- Disruption of the neuropeptide-Y receptor (NPY1R) in mice.
- Knockout of the serotonin 5-HT-2C receptor gene.
- Neuropeptide Y receptor Y2 (NPY2R) knockout mice.

### 2.4. Drug-Induced and Surgery-Induced Models of Obesity

The hypothalamus plays a consequential role in signaling between the gut and brainstem and in regulating signals responsible for energy input and output, among other functions [22]. Chemical models are obtained by generating lesions in specific nuclei of the hypothalamus [22]. The lesions generated there can be produced mechanically, through surgical intervention, by using radio waves or electrolysis, or chemically by deploying neuronal toxins such as bipiperidyl mustard, monosodium glutamate, ibotenic acid, gold thioglucose, and kainic acid [22,45]. Ovariectomy is used as a surgical model to study obesity in women [22].

- Ventromedial hypothalamus damage.
- Hypothalamic paraventricular nucleus damage.
- Arcuate nucleus damage.
- Ovariectomy.

### 2.5. Diet-Induced Obesity (DIO)

Due to the fact that they enable us to mimic the most prevalent underlying factor causing this disease in humans, diet-induced obesity (DIO) animal models are helpful for research into the polygenic origins of obesity: an unhealthy diet [22,24,46,47]. Most widely employed laboratory animals, mice, and rats, are put on a special obesity-inducing diet designed to echo the particularities of the human metabolic syndrome as closely as possible [22].

- High-fat diet (HFD)/exposure to high-fat and palatable diets.
- High-carbohydrate diet (HCD).
- Cafeteria diet (CAF).
- Maternal overfeeding and exposure to high-fat diets.

Animal obesity models have played and continue to play a major role in understanding the intricacies of this disease, which has earned the title of a global epidemic. There is a wide range of models, each with its advantages and disadvantages, allowing researchers many different approaches to preventing or even treating obesity, which affects more than one billion people worldwide.

## 3. Genetic Forms of Obesity

It is well known that the incidence of obesity has increased rapidly with urbanization, but despite the negative effects of an obesogenic society, there is high individual variability in body weight [48,49]. The concept of an innate cause of obesity was described as early as 1907 by Von Noorden, and numerous studies have since strengthened this hypothesis [48,49]. Stunkard et al. (1986) conducted a study of 540 adopted Danish twins and concluded that the weight of the twins in adulthood was the same as that of their biological parents, even when they were raised in adoptive families [50]. Bouchard et al. (1990) showed in their study that there is a statistically significant similarity between monozygotic twins in weight gain (under overfeeding conditions), and a systematic review of twin studies estimated the heritability of obesity to be 45–90% [48,51]. Wardle et al. (2008) supported the hypothesis of a genetic influence on obesity by demonstrating a significant concordance in body weight between monozygotic and dizygotic twins [52].

In recent years, new genes that play a crucial role in this complex trait have been identified as a result of the availability of new and highly accurate diagnostic methods, in particular whole-exome sequencing [48,49]. The genetic factors involved in obesity lead to variable phenotypes, and there are three broad clinical presentations: monogenic obesity, polygenic obesity, and syndromic obesity [49].

Polygenic obesity is the most common phenotypic expression of obesity. It is caused by a complex interaction between genetic susceptibility and the obesogenic environment. Overeating, a sedentary lifestyle, a lack of sleep, and stress all increase individual genetic susceptibility [49,53].

Monogenic obesity is inherited in a Mendelian pattern, is rare, and is characterized by a severe early onset, typically before 10 years old. It is most commonly caused by mutations of the genes involved in the leptin-melanocortin axis, which plays a pivotal role in the hypothalamic control of food intake. This type of obesity is frequently associated with endocrinologic dysfunction [49,54].

Syndromic obesity is a term used when severe obesity is associated with intellectual disability, dysmorphic features, organ abnormalities, or signs of hypothalamic dysfunction. It can be caused by the mutation of a single gene or a larger chromosomal region and transmitted in an autosomal or X linked manner, but it can also be caused by de novo genetic mutations. Over 100 syndromes associated with obesity have been described, but the most common are Prader–Willi (PWS) and Bardet-Biedl (BBS) syndromes. The identification of syndromic obesity is important because it is often associated with specific comorbidities that may require prevention or at least assessment and treatment [49,55].

PWS is the most common cause of syndromic obesity, with an incidence of 1 in 20,000–25,000 births worldwide. It has complex effects on the metabolic, endocrine, and neurological systems. The disease is characterized by severe neonatal hypotonia, feeding difficulties with failure to thrive in the first years of life, followed by hyperphagia and the gradual development of morbid obesity in later childhood, dysmorphic features, developmental delay behavioral problems, hypogonadism, growth hormone deficiency, and hypothyroidism [49,56]. PWS is caused by the inactivation of the critical Prader–Willi region of the paternal chromosome [48].

BBS is a non-motile ciliopathy characterized by retinal dystrophy (progressive night blindness, photophobia, and loss of central and color vision), obesity and its complications, postaxial polydactyly, cognitive impairment, hypogonadotropic hypogonadism, and genitourinary malformations. At least 19 different genes have been implicated in the disease, all of which play a role in the normal functioning of the primary cilium, which appears to play a role in energy homeostasis [48,49,57].

Other causes of syndromic obesity include Cohen syndrome, Alström syndrome, X fragile syndrome, Borjeson–Forssman–Lehmann syndrome, 16p11.2 deletion syndrome, kinase suppressor of Ras2 (KSR2) variants, TUB mutations, ACP1, TMEM18, and MYT1L deletion [49].

The genes identified in monogenic obesity are most commonly involved in the hypothalamic leptin-melanocortin pathway. The central nervous system regulates food intake through the hypothalamic leptin-melanocortin axis, which receives signals from tissues. Signals are received from the gut by hormones such as ghrelin, peptide YY, cholecystokinin, glucagon-like peptide, and mechanoreceptors that measure distention, from the pancreas by insulin, or by the adipokine hormones (leptin and adiponectin). This axis is a key regulator of energy homeostasis and is activated by leptin and insulin receptors on the surface of neurons located in the arcuate nucleus. There are two types of neurons involved in the feedback loop: the pro-opiomelanocortin and cocaine and amphetamine-related transcript neurons (POMC/CART), which play a role in the production of the anorexigenic peptide POMC, and another set of neurons, which play a key role in the production of the orexigenic peptides agouti-related peptide (AGRP) and neuropeptide Y (NPY). POMC is processed by two enzymes to produce peptides such as α-, β- and γ-melanocyte stimulating hormone (MSH) and β-endorphins. AGRP and α-MSH compete for the same receptor, the melanocortin-4 receptor (MC4R); when α-MSH binds to the receptor, the result is anorexigenic signals, but when AGRP binds, the result is orexigenic signals. Mutations at any level of these regulatory mechanisms will result in monogenic obesity [48].

### 3.1. Leptin and Leptin Receptor Mutations

Leptin is a protein encoded by a gene located on chromosome 7q31.3. It is secreted into the blood from adipocytes, crosses the blood–brain barrier, binds to GABAergic neurons in the hypothalamus, induces satiety signals, and inhibits NPY and AGRP. Congenital leptin deficiency is characterized by the rapid onset of severe obesity caused by hyperphagia and its complications such as hyperinsulinemia, hepatic steatosis, and dyslipidemia. These patients may also have hypogonadotropic hypogonadism and T-cell dysfunction, leading to severe infections. Mutations in the gene that encodes the leptin receptors lead to similar symptoms as leptin gene mutations. The Food and Drug Administration has approved the administration of metreleptin for the treatment of congenital leptin deficiency, but it cannot be used in patients with leptin receptor mutations [48,58].

### 3.2. Pro-Opiomelanocortin (POMC) Mutations

Congenital POMC deficiency results in a deficiency of POMC peptides, including ACTH, α-MSH, and β-endorphins. Patients develop early-onset obesity, red hair, and hypopigmentation due to α-MSH deficiency and adrenal insufficiency due to ACTH deficiency, requiring rapid glucocorticoid replacement therapy [48,59].

### 3.3. Melanocortin Receptor (MC4R) Deficiency

MC4R deficiency is the most common cause of monogenic obesity and is typically characterized by obesity, increased bone mineral density, increased linear growth in early childhood, hyperphagia, and severe hyperinsulinemia [48,60].

### 3.4. Proconvertase (PC1/2) Deficiency

Proconvertase deficiency causes severe early-onset obesity, adrenal, gonadotropic, somatotropic, and thyrotropic insufficiency, postprandial hypoglycemia secondary to reduced insulin processing, severe neonatal malabsorption, and central diabetes insipidus [48].

### 3.5. Single-Minded Homologue of Drosophila (SIM1) Deficiency

SIM1 is expressed in the hypothalamus, and its mutations are associated with hyperphagia and food impulsivity with consequent obesity, impaired concentration, memory deficit, emotional lability, or autism spectrum disorder [48].

### 3.6. NTRK2/BDNF Mutations

Brain-derived neurotrophic factor (BDNF) regulates the energy balance through two receptors, p75 and TrkB. Mutations in the gene for TrkB but not p75 can cause severe obesity [48,61].

### 3.7. SH2B1 Mutations

Src homology 2 B adapter protein 1 (SH2B1) is a key endogenous positive regulator of leptin sensitivity. SH2B1 mutations are associated with severe early-onset obesity, insulin resistance, reduced height, hyperinsulinemia but without diabetes, delayed speech, and aggressive behavior [48,62].

### 3.8. Kinase Suppressor of Ras 2 (KSR2) Mutations

KSR2 mutations lead to obesity due to hyperphagia, a reduced metabolic rate, and severe insulin resistance [48].

## 4. Oxidative Stress and Obesity

Oxidative stress is defined as an imbalance between the production and scavenging of various ROS and reactive nitrogen species (RNS); this imbalance occurs either through the overproduction of reactive species or through a reduction in their defense mechanisms. ROS include molecules such as hydrogen peroxide (H_2_O_2_), superoxide (O_2_^•−^), and the hydroxyl radical (OH^−^); at the cellular level, several sources of ROS are recognized: leakage of electrons from complexes I and III of the electron transport chain with formation of superoxide (electron leakage), increase in the redox status of macrophages and neutrophils in the presence of pathogens (respiratory burst), and formation of superoxide radicals in reactions catalyzed by NADPH oxidases (NOXs) and xanthine oxidase (XO) [63,64,65,66].

The mechanisms that protect the cell from the damaging action of free radicals work with the help of enzymatic and non-enzymatic antioxidant systems; the enzymatic system comprises enzymes (e.g., glutathione peroxidases (GPXs), catalase (CAT), superoxide dismutases (SODs), peroxiredoxins (PRDXs)) localized in different cell compartments (mitochondrial matrix, cytosol, peroxisomes) [67]. Endogenous cofactors (glutathione, lipoic acid) and exogenous antioxidants from the diet (vitamin C, vitamin E, carotenoids, polyphenols, selenium, and zinc) also contribute to antioxidant defense mechanisms [68,69].

Scientific data links oxidative stress to obesity. Some mechanisms, such as hyper-glycaemia, chronic inflammation, increased free radical formation, increased muscle lipids, hyperleptinemia, low antioxidant capacity, altered mitochondrial function, and endothelial dysfunction, may explain the association of obesity with increased ROS production. [70,71,72].

During prolonged hyperglycemia, advanced glycosylation end products (AGE) lead to the activation of some cell surface receptors (RAGE), which activate nuclear factor-kB (NF-kB). This activation initiates the transcription of intracellular adhesion molecule-1 (ICAM-1) and vascular cell adhesion molecule (VCAM-1), which promote ROS production and inflammation [73,74]. Another mechanism that favors free radical formation in obese patients with hyperglycemia is the stimulation of the polyol pathway, in which glucose is converted to sorbitol, a compound that has been shown to cause oxidative damage [75]. In addition, sorbitol formation by aldose reductase uses NADPH and thus indirectly reduces available reduced glutathione, which exacerbates the oxidative effect [76,77].

Inflammation is a known cause of oxidative stress in obesity. Overweight patients have low-grade chronic inflammation and increased oxidative stress. Several inflammatory triggers have been suggested, such as hypoxia in adipose tissue, mechanical stress due to the interaction between the extracellular matrix and hypertrophic adipose tissue, and increased intestinal permeability that may lead to the presence of gut-derived antigens in plasma and dietary components. The mechanism of obesity-related inflammation is not fully understood, but an increase in inflammatory markers has been observed in obesity [78]. It is thought that the pro-inflammatory cytokines (tumor necrosis factor-alpha (TNF-α), interleukin (IL)-1β and IL-6) released by hypertrophic adipose tissue activate monocytes and their differentiation into macrophages with an increase in oxidative stress [79]. Activation of the NF-κB pathway by cytokines may also play a role in ROS production [80,81]. Furthermore, at the adipose tissue level, adipokines may stimulate chemotaxis with macrophage recruitment [82].

Adipokines, including leptin, adiponectin, visfatin, resistin, apelin, and plasminogen activator inhibitor type 1 (PAI-1), are secreted by dysfunctional adipose tissue and play a role in both inflammation and the initiation and maintenance of oxidative stress [78,83]. Leptin has a hypothalamic-mediated effect on food intake, and its plasma concentration is proportional to adipose tissue expansion. Several mechanisms explain the link between leptin and oxidative stress: direct production of H2O2 and OH-, decrease in antioxidant enzyme synthesis (paranoxaze-1), stimulation of monocyte proliferation and their transformation into macrophages, and also the production of inflammatory cytokines that increase NADPH oxidase production [70]. Visfatin, one of the recently discovered adipokines, increases the production of pro- and anti-inflammatory cytokines and generates ROS, particularly superoxide, and H2O2. It has been shown that visfatin-mediated ROS generation is associated with phosphorylation of the NF-κB pathway and is independent of activation of mitogen-activated protein kinases (MAPKs) [84].

Other mechanisms of free radical generation in obesity include mitochondrial and peroxisomal oxidation reactions during fatty acid degradation. Hyper-trophic adipocytes in obese patients have a lower density of insulin receptors and a higher density of beta-3 adrenergic receptors, favoring lipolysis mechanisms with the formation of free fatty acids (FFA) [85]. The production of FFA leads to the accumulation of fats and glucose in adipose, muscle, and liver tissues, which promotes the increased synthesis of free radicals through oxidative reactions [86,87].

Obesity may also induce oxidative stress through more complex mechanisms involving respiratory dynamics. Overweight patients have been shown to have an increased respiratory rate and may have mild hypoxemia with high free radical production [88]. Furthermore, obstructive sleep apnea is often observed in obese patients [89]. This pathology causes episodes of hypoxia/reoxygenation, which is also a cause of oxidative stress [90].

Although free radicals play a physiological, local role in processes such as antibody defense, apoptosis, and necrosis, they also exert systemic deleterious effects by damaging lipids, proteins, carbohydrates, and DNA [91]. On a macroscale, these phenomena manifest as damage to the cardiovascular, neurological, digestive, and renal systems and may play a role in carcinogenesis [92,93,94,95,96].

We have summarized some of the mechanisms that generate oxygen-free radicals in obesity. It seems that the increase in oxidative stress in obese people is known and leads to several comorbidities associated with obesity, but not all mechanisms that generate free radicals are fully understood. Further studies are needed to demonstrate both the influence of free radicals on obesity and the mechanisms by which obesity may generate oxidative stress. Since some of the comorbidities associated with obesity are life-threatening, it is necessary to find new therapies that target the molecules involved.

## 5. Environmental Factors and Obesity

Persistent organic pollutants (POPs) are organic chemicals that are persistent, semi-volatile, and bioaccumulative and are considered a major threat to human health and the world’s ecosystems [97]. Approximately 900 active compounds are currently used as pesticides worldwide in 60,000 preparations used to treat more than 4 billion hectares of land [98]. Recently identified POPs have adverse effects on humans and can be divided into three categories:

- Pesticides: aldrin, chlordane, dichlorodiphenyltrichloroethane (DDT), dieldrin, endrin, heptachlor, hexachlorobenzene, mirex, toxaphene;
- Industrial chemicals: hexachlorobenzene, polychlorinated biphenyls (PCBs);
- By-products: hexachlorobenzene; polychlorinated dibenzo-p-dioxins; polychlorinated dibenzofurans (PCDD/PCDF); and PCBs [99].

These chemicals and some solvents cause weight gain, and it is proposed that they interfere with weight homeostasis by altering weight-controlling hormones, modifying sensitivity to neurotransmitters, or altering sympathetic nervous system activity [100]. Environmental factors, including agrochemicals, may contribute to the rapid increase in the incidence of obesity, T2D (type 2 diabetes), and other aspects of the MetS (metabolic syndrome) observed in recent decades [101]. Although the mechanisms by which environmental chemicals induce obesity are not fully understood, several hypotheses have been put forward to explain the obesogenic effect [102].

Adipocytes are known to arise during embryogenesis from mesenchymal cells derived from the lateral layer of the mesoderm. Adipogenesis is a complex, multi-component, controlled process during which mesenchymal stem cells (MSCs) differentiate into mature adipocytes. Adipogenesis involves two stages: determination and terminal differentiation. In the determination phase, pluripotent stem cells are transformed into unipotent preadipocytes. During terminal differentiation, preadipocytes acquire the phenotype and functional characteristics of mature adipocytes. The current consensus is that the number of white adipocytes is fixed by the end of childhood and that all factors that increase the number of adipocytes in early life lead to a lifelong increase in the number of white adipocytes [103]. Early life events, including exposure to obesogenic factors, that alter the fate of MSCs could predispose the exposed individual to increased numbers of white adipocytes and consequently obesity [104].

There are several signaling pathways that converge to regulate MSC differentiation:

1. Peroxisome proliferator-activated receptor γ (PPARγ)-dependent pathway. PPARγ is a marker of adipocyte differentiation, and its ectopic expression is sufficient to induce fibroblast transformation according to the adipogenic program. PPARγ can bind to bisphenol A (BPA), perfluorinated compounds (PFCs), and phthalates, resulting in stimulation of adipogenesis in vitro and in vivo by promoting differentiation and acquisition of mature adipocyte characteristics such as insulin sensitivity, lipid synthesis and transport, and secretion of adipocyte-specific proteins [102,105,106]. Using a human adipose-derived stromal cell-based adipogenesis assay, Foley et al. found that some agrochemicals, including triphenyltin hydroxide, lactofen, triflumizole, halosulfuron-methyl, cyfluthrin, flufenacet, isoxaflutole, piperonyl butoxide, pyraclostrobin, and tebufenozide, may have moderate to strong activity on human adipogenesis [107].
2. Sex steroid hormones play an important role in the development of adipose tissue, and their imbalance can lead to dyslipidemia and obesity [108]. Many agrochemicals, such as dichlorodiphenyldichloroethylene (DDE), have been shown to inhibit 5α-reductase, the main enzyme that converts testosterone to dihydrotestosterone [109]; others, including DDT, can affect the expression levels and/or activity of several cytochrome P450 (P450) enzymes involved in the metabolism of steroid hormones [110,111] and the development of obesity. Numerous in vitro studies using reporter gene assays have demonstrated the estrogenic and antiandrogenic effects of agrochemicals [108].
3. There is considerable evidence of a strong link between exposure to endocrine disrupting chemicals (EDCs) such as polychlorinated biphenyls (PCBs), polybrominated diphenyl ethers (PBDEs), perfluoroalkyl substances (PFASs), phthalates, BPA, and perchlorate and thyroid disorders [112]. Many of these chemicals alter the balance between energy storage and expenditure, disrupting the thyroid signaling pathway and contributing to the development of obesity [113].
4. Numerous studies have shown that agrochemicals could affect the composition and function of gut microbiota and play an important role in agrochemical-induced toxicity [114,115,116]. Serum levels of organochlorine pesticides (cis-nonachlor, oxychlordane, and trans-nonachlor) were positively correlated with high levels of *Methanobacteriales*, which were associated with higher body weight and waist circumference [117].

In summary, technical and scientific progress has reached a high level in all areas, including organic chemicals, which are widely used in agriculture. However, pollutants and agrochemicals play a significant role in the development of obesity through various mechanisms and effects. We stress the importance of involving the public in the decision-making process for the monitoring and control of POPs. This process should be transparent to all interested groups of citizens and should provide free access to data on POPs.

## 6. Stress, PTSD, and Obesity-Related Morbidities

### 6.1. Stress

The discussion about obesity would not be complete without placing it in the background of everyday challenges, including stress and the extreme phenomenon of PTSD (which allows a better understanding of stress only by magnifying some of its features). Obesity cannot be reversed over a long period of time without resolving the trauma that caused it [118]. A major problem is the increasing stigmatization of the obese population; the prejudice associated with the personality and socio-professional performance of obese people only reinforces the vicious cycle of eating disorders [119]. The reduced physical activity that accompanies this new paradigm of living in a digital ecosystem, surrounded by an obesogenic digital food environment, reduces healthy ways of reducing stress and encourages the development of obesity [119,120].

Stress is a common contributor to the onset and progression of addiction [121]. Given that obese people report greater food cravings and consume larger portions in response to food cues (sights, sounds, and smells), obesity can be seen as a result of food addiction [122]. Moreover, flavor additives in ultra-processed foods increase palatability, promote hedonic eating, and interfere with homeostatic control of food intake [123]. Increased activation of mesocorticolimbic circuits, particularly dopamine and glutamate transmission in the nucleus accumbens (NAc), has been shown to correlate with weight gain and difficulty in weight loss. On the other hand, experimental data indicate that obese rats are more susceptible to the effects of cocaine, suggesting a bidirectional connection between weight gain and activation of the reward system [122,124]. Severe obesity has also been linked to decreased brain expression of D2 receptors, which indirectly stimulate endorphin release and appetite [125].

Strong and sustained activation of the sympathetic nervous system (SNS) and hypothalamic-pituitary-adrenal (HPA) axis during stress leads to hyperglycemia, hypercortisolism, inflammation, and insulin resistance [126,127]. Furthermore, Kim points out that the main deleterious effects of stress are caused primarily by alterations in neural circuits that affect the functions of all body systems [128]. Brain-induced stress changes are reversible, with the prefrontal cortex (PFC) and the hippocampus (HPC) recovering more quickly than the amygdala [129].

In acute stress, hyperglycemia represents a survival-optimizing response, but prolonged allostatic load from chronic stress leads to a downward spiral of metabolic imbalance and increases the risk of T2D [129,130]. In metabolic syndrome, hyperinsulinemia favors activation of the sympathetic nervous system, as shown by increased plasmatic and urinary concentrations of norepinephrine (NE) [131,132,133]. Stimulation of the parasympathetic nervous system improves glycemic control and insulin sensitivity in prediabetic patients [134].

In addition, stress-induced hypercortisolemia leads to changes in the brain circuits that control food intake, favoring increased intake of palatable foods [129]. Trauma in childhood, in particular, has a very detrimental effect on brain development [135].

The metabolism of lipids is deeply affected by stress, as demonstrated by the effect of stress on enzymes such as hepatic lipase, lipoprotein lipase, cholesterol esterase, HMG-CoA reductase, acyltransferase, acyl-CoA dehydrogenase, and others [134].

### 6.2. PTSD

Epidemiological studies in pediatric and adult populations show that PTSD is associated with a variety of comorbidities and doubles the risk of T2D, metabolic syndrome, and obesity [16,118,131,136,137,138,139,140,141,142,143,144,145]. Interestingly, a reduction in body weight may predict improvement in PTSD, and recovery from PTSD is associated with a reduced risk of T2D [137,146,147]. In PTSD, obesity caused by HPA dysregulation is exacerbated by unhealthy behaviors (high-fat, high-carbohydrate eating, smoking, alcohol consumption, reduced adherence to medication, sedentarism, insomnia) [131,148,149].

According to the DSM-5 criteria for PTSD, a person who has been exposed to a horrific event (either directly or by learning that a relative or close friend has suffered trauma) may experience unwanted disturbing memories, nightmares, emotional distress, and physical reactivity [150]. Studies about at-risk populations (refugees, victims of war, natural disasters, terrorism, domestic violence and accidents, sexual assault, healthcare workers during the COVID pandemic, COVID survivors, and war veterans) have found prevalence rates between 5% and 20% [131]. However, the actual prevalence could be even higher, as only part of the exposed population seeks treatment due to social stigma or a lack of resources [151,152,153,154]. Normally, in some situations, specific dental problems found in PTSD (myofascial pain, tooth bruxism, temporo-mandibular joint disorders, and tooth sensitivity) should alert the healthcare professional and facilitate the diagnosis [155].

Simplistically, PTSD is a fear disorder caused by exaggerated consolidation of fear memory or impaired extinction learning of trauma-related fear. This has profound effects on the entire body, leading to alterations of the neurotransmitters and the neuroendocrine system, mitochondrial dysfunction, metabolic changes, and proinflammatory changes [131,136,151,156,157]. Interestingly, only a small proportion of individuals exposed to a similar type of stress develop PTSD, so there is a genetic susceptibility that favors the pathology. Moreover, genotypes with the same SNP manifest different phenotypes under similar circumstances, which explains the heterogeneity of PTSD subtypes [158]. Most studies suggest that PTSD is characterized by low cortisol levels, but there are also studies showing that PTSD is correlated with high cortisol levels [140,156].

In obese patients, inflammation is triggered by sympathetic-induced activation of innate immunity, which promotes infiltration of M1-polarized macrophages in adipose tissue and lymphocytes and monocytes in the pancreas [129,131]. Chronic inflammation associated with prolonged stress opens up a vicious cycle as cytokines such as IL-6, the markers of inflammation, stimulate the stress response [134,159].

High levels of inflammatory markers such as TNF-alfa, IL -1beta, IL-6, and CRP are elevated in both obesity and PTSD, indicating a common pathological link [104,106,111,113,135,136]. The bidirectional relationship between obesity, inflammation, and PTSD is also confirmed by the fact that TNF-alfa is associated with prolonged fear memory and administration of anti-inflammatory therapy reduces anxiety symptoms [106,111,137]. Moreover, high TNF levels have been associated with alterations in insulin signaling, and treatment with neutralizing TNF antibodies has improved insulin resistance [159].

Recent data suggest that glucocorticoids increase the expression of fatty acid synthase and play an important role in the formation of adipose tissue by facilitating the conversion of pre-adipocytes into mature adipocytes [129].

Most of the hormonal regulators of appetite interfere with the fear response, either reinforcing the memory of the fear or enabling the extinction of the traumatic recollections.

Body weight depends on a multitude of factors, including those that regulate appetite. Two clusters of neurons located in the hypothalamic infundibular nucleus are important in this direction: POMC neurons that release α-MSH with anorexigenic properties and NPY neurons that release NPY orexigenic peptide [129,160,161]. These neuronal populations express leptin and insulin receptors that allow fine regulation in response to dynamic changes in metabolic status.

Interestingly, as POMC neurons exhibit increased vulnerability to stress, NPY action dominates, and the result leads to increased appetite and food intake [162]. NPY induces anxiolysis through inhibitory effects on corticotropin-releasing hormone (CRH) and norepinephrine signaling, reduces the acquisition of contextual fear memories, and stimulates the extinction of conditioned fear memories [131,163]. Glucocorticoids increase NPY levels, and obese individuals exhibit high levels of this hormone [129,131]. In contrast, PTSD is characterized by low levels of NPY; however, these levels reach the normal range after recovery from the illness or the administration of fluoxetine [131,161].

In PTSD, leptin levels are considered a neuroendocrinological marker for the hypervigilant state [161]. Although leptin exhibits anorexigenic effects and promotes the extinction of fear memories, hyperinsulinemia in PTSD patients results in leptin resistance, leading to increased appetite, decreased memory performance, and impaired synaptic plasticity in the hippocampus [16,131,138]. Normalization of insulinemia after weight loss attenuates leptin resistance and increases NPY levels, with beneficial effects on the HPA axis [138].

Ghrelin helps maximize individual survival during stress, facilitates the encoding of fear memories, and stimulates appetite [131]. Stress has been shown to increase ghrelin levels.

Adiponectin, the most abundant adipokine, facilitates environmental adaptation by reducing contextual fear memories and is inversely correlated with PTSD severity [161]. As adiponectin acts as a potential risk biomarker, the pharmacological elevation of adiponectin levels is a conceivable treatment for this pathology [138,144,164]. There is a parallel between PTSD and obesity, as low adiponectin levels correlate with obesity, and weight loss is associated with increased levels and improved insulin sensitivity [165].

Insulin-like growth factor 1 (IGF1) acts on orexin neurons from the lateral hypothalamus and exhibits anxiolytic properties, modulates coping behavior, reduces vulnerability to stress, but also promotes obesity due to stimulation of cell proliferation [166]. Part of its effect is due to the modulation of the GABA, glutamate, and serotoninergic systems [167].

High insulin levels in people with obesity and metabolic syndrome lead to low insulin sensitivity in the brain, decreased dopamine release after food intake, and consequently, an increased need to eat high-calorie, palatable foods [129].

A high-fat, high-sugar diet, obesity, or early stress exposure in rats reduces BNDF signaling and levels, leading to altered neuroplasticity and impaired learning [135,168]. BNDF activates the HPA axis by stimulating the release of CRH.

The main pathological link between obesity and stress is the dysregulation of the HPA axis. Some polymorphisms of genes involved in optimal HPA axis function (FKBP5, NR3C1, CRHR1, SLC6A4, and OXTR) have been associated with increased susceptibility to stress and a high risk of mental disorders [156,158,169,170]. The heritability of PTSD is estimated to be up to 20% [151,171].

Furthermore, exposure to stress leads to epigenetic changes (particularly affecting methylation) that are passed on transgenerationally, probably as an evolutionary adaptation to prolonged adverse living conditions [142]. Low methylation in the FKBP5, NR3C1, and CRHR1 genes has been associated with childhood adversity, and reduced methylation in the insulin-like growth factor 1 (IGF2) genes has been demonstrated in adults whose pregnant mothers suffered starvation in the winter of 1944–1945 [142,156]. Most interesting is the phenomenon described in Holocaust survivors and their offspring, where the first generation showed high methylation levels, whereas the second generation showed low methylation levels and lower self-reported anxiety [172,173].

After exposure to stress and elevated cortisol levels, FKBP5 (allele rs1360780) shows demethylation in intron 7 and consequently increased transcription of FKBP5 [169,174]. As FKBP5 is a co-chaperone of GR that inhibits GR binding and nuclear translocation of the ligand-receptor complex, increased expression of FKBP5 results in inhibition of glucocorticoid (GC) action and GC resistance [135,156,175,176]. GC resistance, along with HPA axis dysregulation and hypercortisolism, precedes hyperinsulinemia and obesity. The chronic fatigue associated with GC resistance further increases the risk of developing obesity [177,178].

Interestingly, increased methylation of SNPs in the FKBP5 gene (e.g., rs9296158) and some CRHR1 gene polymorphisms that produce high levels of CRH are associated with cortisol hypersensitivity and hypercortisolism and represent a distinct PTSD phenotype from that characterized by hypercortisolism [156].

As decreased anxiety levels are associated with low methylation of FKBP and low methylation is provoked by GC concentration, together with the fact that hypercortisolism is considered a risk factor for PTSD, it can be hypothesized that administration of dexamethasone/corticosterone at the time of trauma might improve memory extinction and interrupt the vicious cycle of HPA dysregulation and obesity [131,142,156,174]. Other pharmacological interventions for PTSD include selective FKBP5 blockers and CRHR1 antagonists [156,158,179]. Given the pathological processes that characterize PTSD, there are also some therapies that can alleviate the severity of the condition: metformin, thiazolidinediones, PPAR agonists, angiotensin receptor type I blockers, angiotensin converting enzyme inhibitors, cannabinoid receptor antagonists (upregulation of adiponectin), quercetin, coenzyme Q10, and resveratrol (improvement of mitochondrial dysfunction) [136]. Given the high cost of treating patients, early intervention and treatment are beneficial for the best outcomes.

The strong correlation between the methylation process and the severity of PTSD is also underlined by the fact that mindfulness and cognitive behavioral therapy reduced FKBP5 methylation and alleviated clinical symptoms [158,180].

Epigenetic regulatory mechanisms following stress/traumatic events have also been described for BDNF genes, IGF2, and the CYP17A1 gene, which is involved in the production of the enzyme 17-alpha-hydroxylase, which converts cortisol to cortisone [142].

Both obesity and PTSD can be described as metabolic disorders in which mitochondrial dysregulation is the central cellular alteration [136,181,182]. These pathologies also share common features such as dysregulation of fatty acid metabolism, disruption of the tricarboxylic acid cycle, and altered peroxisome proliferator-activated receptor (PPAR) function [136,181,183,184].

Another link between obesity and PTSD is demonstrated by functional magnetic resonance imaging, which shows alterations in analogous brain areas responsible for emotion, memory, reward, motivation, and volitional control [16,185,186]. Obese individuals showed greater food cue-induced activation in the hippocampus/amygdala, hypofunction of the medial and dorsal prefrontal cortex responsible for inhibitory processes, and neurodegeneration-induced reduced hippocampal volume [185,186,187,188].

Interestingly, obesity is associated with reduced hippocampal volume, but hippocampal deficits facilitate an enhanced stress response, data that further emphasize the bidirectional relationship between obesity and stress [189]. Animal models, as well as clinical data, suggest that acute stress disrupts activity in the prefrontal cortex and amygdala [190].

Altered activity in the prefrontal cortex predisposes to increased food intake, as the immediate reward of eating (due to dopamine release in the amygdala) overrides awareness of the long-term negative consequences of choosing unhealthy foods [191,192]. Interestingly, atenolol and propranolol reduce psychological distress by reducing adrenergic activity, making beta-blockers useful in the treatment of anxiety and PTSD (when given immediately after the event) [193,194].

Recent data suggest that obesity is associated with cognitive performance decline, with metabolically unhealthy individuals showing a faster rate of deterioration than the rest of the population [195]. Chronic exposure to GC, hyperinsulinemia, and insulin resistance impair synaptic plasticity, affecting learning and memory [196,197]. Stress-induced dysbiosis also disrupts the blood-brain barrier.

### 6.3. Obesity Never Comes on Its Own

It is unanimously accepted that obesity has been identified as a multifaceted risk factor for a range of serious medical conditions, including but not limited to type 2 diabetes, cardiovascular disease, gallstone disease, non-alcoholic fatty liver disease, acute pancreatitis, certain cancers, chronic kidney disease, ischemic stroke, obstructive sleep apnea, osteoarthritis, psychiatric comorbidities, and infertility [198].

Understanding the link between obesity and its associated complications is of paramount importance for developing effective prevention and management strategies. One of the main pillars of current public health policy is the association between T2D and obesity. As we know to date, T2D has reached epidemic proportions worldwide, and its strong association with obesity has been well established [199]. Obesity, characterized by excess adiposity, particularly visceral adipose tissue, is a major risk factor for the development of insulin resistance and subsequent T2D. The adipose tissue itself functions as an active endocrine organ, releasing a variety of adipokines, such as leptin, adiponectin, and resistin, which play crucial roles in glucose homeostasis and insulin sensitivity. In obesity, the dysregulation of adipokine secretion leads to a state of chronic low-grade inflammation and adipose tissue dysfunction, contributing to the pathogenesis of insulin resistance [200]. Additionally, adipose tissue expansion is accompanied by an increased release of free fatty acids into the circulation, which further promotes insulin resistance in peripheral tissues such as skeletal muscle and the liver. Adipose tissue dysfunction also impairs the production and release of adiponectin, an adipokine with insulin-sensitizing properties, further exacerbating insulin resistance [201]. Furthermore, obesity is closely linked to other metabolic abnormalities, including dyslipidemia, hypertension, and systemic inflammation, collectively referred to as metabolic syndrome. These comorbidities further contribute to the development and progression of T2D. Importantly, the bidirectional relationship between obesity and T2D creates a vicious cycle, as hyperglycemia resulting from insulin resistance promotes further adipose tissue dysfunction and exacerbates obesity [202].

In addition to the well-known metabolic and cardiovascular complications associated with obesity, emerging evidence suggests a link between the novel concepts of diabetes, non-alcoholic fatty liver disease (NAFLD), and thrombosis [203]. Insulin resistance, a key feature of T2D, plays a central role in the development and progression of NAFLD. It promotes hepatic lipid accumulation by enhancing the release of free fatty acids from adipose tissue and impairing lipid oxidation in the liver. On the other hand, NAFLD contributes to the development and exacerbation of insulin resistance by releasing inflammatory cytokines and adipokines that interfere with insulin signaling pathways. Moreover, both conditions are associated with systemic inflammation, oxidative stress, and dyslipidemia, which further fuel the pathogenesis of each other [204]. The presence of NAFLD in individuals with T2D is associated with an increased risk of developing advanced liver disease, including non-alcoholic steatohepatitis (NASH), fibrosis, and cirrhosis. Conversely, T2D is a strong predictor of NAFLD progression and the development of complications, such as hepatocellular carcinoma [205].

While NAFLD is primarily considered a hepatic manifestation of the metabolic syndrome, emerging evidence suggests that it is also associated with an increased risk of thrombotic events. The mechanisms underlying the association between NAFLD and thrombosis are complex and multifactorial. One proposed mechanism is the systemic inflammation and endothelial dysfunction observed in NAFLD, which can promote a prothrombotic state. Inflammatory cytokines, such as TNF-alpha and IL-6, derived from the liver and adipose tissue, contribute to endothelial dysfunction and activation of the coagulation cascade [206]. Furthermore, NAFLD is often accompanied by metabolic abnormalities, including insulin resistance, dyslipidemia, and hyperglycemia, all of which can further contribute to the prothrombotic milieu. Additionally, NAFLD is associated with alterations in coagulation factors and fibrinolytic pathways, such as increased levels of plasminogen activator inhibitor-1 (PAI-1), decreased tissue plasminogen activator (tPA) activity, and elevated levels of fibrinogen [207]. These abnormalities may disrupt the delicate balance between coagulation and fibrinolysis, favoring thrombus formation. The bidirectional relationship between NAFLD and thrombosis highlights the interplay between liver dysfunction, metabolic derangements, and the coagulation system. Recognizing the association between obesity and thrombosis is crucial for risk assessment, prevention, and management strategies. The identification of novel biomarkers, such as adipokines, inflammatory markers, and endothelial dysfunction markers, has provided insights into the underlying mechanisms linking obesity and cardiovascular events [208]. Furthermore, lifestyle interventions targeting weight loss, physical activity promotion, and dietary modifications have demonstrated their effectiveness in reducing cardiovascular risk in obese individuals with T2D. Pharmacological interventions, including anti-diabetic medications and lipid-lowering agents, play a crucial role in managing T2D and mitigating cardiovascular risk. In conclusion, understanding the complex interplay between obesity, T2D, and cardiovascular events is essential for developing effective preventive strategies, early detection, and comprehensive management approaches to reduce the burden of cardiovascular disease in this high-risk population.

## 7. Microbiota, Branched Chained Amino Acids and Obesity

### 7.1. Microbiota

As obesity has become a global epidemic in recent years, leading to a serious health crisis, it has been the subject of much research. It is well known that the etiology of obesity is a complex interaction between dietary, genetic, endocrinological, environmental, socio-economic, and cultural factors [209]. Recent research emphasizes the role of the gut microbiota, an important environmental factor, not only in the pathophysiology of obesity but also in the pathophysiology of related metabolic disorders. The gut microbiota appears to influence not only the progression of obesity but also its onset [210,211].

The gut microbiome of healthy, normal-weight individuals is complex and diverse, consisting mainly of *Firmicutes*, *Bacteroides*, *Proteus*, *Actinomycetes*, *Fusobacteria,* and *Verrucomicrobia* [212]. The gut microbiota consists of a dominant, sub-dominant, and transient microbiota [213]. The diversity of the gut microbiota can fluctuate rapidly over a few weeks, depending on dietary intake, stress levels, hormones, and physical activity, while another component is stable and resilient. The latter consists mainly of microorganisms inherited at birth and those established during the first years of life (especially the first 1000 days) [214,215]. The gut microbiota has a variety of important functions, such as the biodegradation of polysaccharides, the production of short-chain fatty acids, the enrichment of specific lipopolysaccharides, and the production of vitamins and essential amino acids [212]. Any disturbance of the structure, functions, and metabolism of the gut microbiota leads to pathological processes, including obesity [212].

An impoverished microbiota, with low microbial diversity or an excess of Gram-negative bacteria, tends to increase local inflammation in the gut and the porosity of the intestinal epithelium. This increase in intestinal permeability allows unwanted components (food fragments or microbes, toxins, and endotoxins, including lipopolysaccharides) to cross the intestinal barrier and enter the body, leading to chronic inflammation and insulin resistance. Recent data suggest that obesity and related diseases are associated with profound gut dysbiosis [216,217]. Several studies have shown that the diversity of the microbiota is lower in obese individuals [212].

In addition, several studies have shown that stress is associated with changes in the composition of the microbiota, the levels of junction proteins (both in the gastrointestinal tract and in the brain), and the concentration of monoamine transmitters [218,219]. The mechanisms linking stress and alterations of the gut microbiota are unknown; however, there has been evidence that mucin secretion, impaired gut motility, and norepinephrine-induced modifications in gene expression of some bacteria could contribute to changes in the microbiota. In addition, digestive microbiota may be altered by changes in dietary patterns, such as increased consumption of highly palatable foods encountered during periods of stress [218].

In 2004, Bäckhed et al. were the first to substantiate the hypothesis that gut microbiota is linked to obesity. In their studies with germ-free mice, they showed that transplantation of gut microbiota from conventionally reared mice into germ-free mice resulted in increased monosaccharide absorption, hepatic lipogenesis, and insulin resistance under starvation conditions, leading to the accumulation of excess adipose tissue [220]. The composition of the gut microbiota is even distinctly related to the degree of obesity [221].

There is strong evidence in the literature from sequencing of the 16S rRNA gene that obesity is associated with two dominant bacterial phyla: *Firmicutes* and *Bacteroidetes*. There are studies in mice that conclude that in obese individuals, the concentration of *Bacteroidetes* decreases by 50% and the concentration of *Firmicutes* increases proportionally [222,223]. Later, similar disturbances in the gut microbiota were found in obese children, and it was hypothesized that changes in *Firmicutes* and *Bacteroidetes* phyla may be an important indicator of childhood obesity [224,225,226,227,228]. A high concentration of *Firmicutes* is associated with markers of brown adipocytes in subcutaneous adipose tissue (but not in visceral adipose tissue), suggesting a positive effect on subcutaneous obesity [212]. However, there are conflicting results in the literature, as there are studies that report a statistically insignificant change in the concentration of *Bacteroides* and *Firmicutes* phyla in obese individuals [212,229,230].

Over time, many researchers have linked obesity to specific bacteria, such as the family *Christensenellaceae* and the genera *Methanobacteriales*, *Lactobacillus, Bifidobacteria,* and *Akkermansia* [212]. The *Christensenellaceae* family belongs to the phylum *Firmicutes* and is positively associated with weight loss and inversely associated with body mass index [231].

Depommier et al. (2019) conducted a randomized, double-blind, placebo-controlled pilot study in overweight and obese insulin-resistant volunteers with the primary endpoint of assessing safety, tolerability, and metabolic indicators (insulin resistance, circulating lipids, visceral adiposity, and body mass) after daily oral supplementation with *Akkermansia muciniphila* bacteria [232]. They showed that supplementation with *Akkermansia muciniphila* improved metabolic indicators and reduced body weight, fat mass, and hip circumference compared with the placebo group [232].

Croversy et al. (2017) showed that the effects of *Lactobacillus* on body weight are species-specific, as they found that *Lactobacillus paracasei* is inversely associated with obesity, whereas *Lactobacillus reuteri* and *Lactobacillus gasseri* are positively correlated with obesity [233]. Considering these results, it seems that bacteria of the same genus can affect the gut microbiota differently. In addition, low levels of *Bifidobacterium* are also associated with obesity [212]. The bacterium *Methanobrevibacter smithii* and the genera *Faecalibacterium, Oscillibacter,* and *Alistipes* are found in lower concentrations in the gut microbiota of obese people than in normal-weight people [211].

The microorganisms found in the gut microbiome of obese people induce a number of molecular processes that ultimately promote obesity. An important role is attributed to the metabolites of the gut microbiome: lipopolysaccharides (LPS) and short-chain fatty acids (SCFAs). Microorganisms from the gut microbiota may be a trigger for the systemic inflammation found in obese individuals. LPS, a membrane component of Gram-negative microorganisms, induces a chronic inflammatory process, and SCFAs induce adipocyte differentiation and promote adipogenesis. In obese individuals, LPS causes metabolic endotoxemia and chronic inflammation, leading to adipocyte hyperplasia and the proliferation of adipocyte precursors, whereas in normal-weight individuals, the microbiome can reduce intestinal LPS levels and thus adipogenesis [209].

Dysbiosis also affects the secretion of inflammatory cytokines, and it is known that obesity is associated with high levels of IL-6, TNF-alpha, and C-reactive protein [234].

Recent studies have shown that obese individuals have a distinct gut microbiota profile that regulates adipocyte accumulation and is more adept at extracting energy from the diet than normal-weight individuals, which appears to be related to a high concentration of short-chain fatty acids (SCFAs) [213,220,235]. Teixeira reported that a higher concentration of stool SCFAs is associated with increased waist circumference and adiposity [236]. Although SCFAs have beneficial effects on maintaining normal body weight by regulating appetite and lipid and glucose metabolism, when they are in excess, they extract more energy from the diet and promote weight gain [209].

The gut microbiota also influences the regulation of the gut-brain axis through peptide YY, pancreatic polypeptide, and glucagon-like peptide 1, which are secreted by enteroendocrine cells that are widely distributed throughout the gut epithelium. These hormones have anorexigenic effects when they bind to receptors in the brain and influence feeding behavior, and there is evidence that their concentration is lower in obese subjects than in non-obese subjects. There are also studies showing that the expression of genes encoding anorexigenic neuropeptides, such as proglucagon and BDNF, is decreased in obese people [210,212,237].

The gut microbiome also influences the secretion of GABA and serotonin, both of which affect appetite control. GABA stimulates feeding, while serotonin suppresses appetite and maintains balance [210].

Microorganisms from the gut microbiota can alter the expression of the fasting-induced adipose factor. Fasting-induced adipose factor is an inhibitor of lipoprotein lipase, and as a result of its alteration, triglycerides will accumulate in adipose tissue. A high level of triglycerides in adipocytes will lead to the accumulation of triglycerides in other organs, and this process will eventually induce insulin resistance [209].

As the gut microbiome is an essential factor in the pathophysiology of obesity, it may be a potential target for new therapeutic approaches. Due to the close relationship between microbiota and peripheral and central nervous system function, the concept of psychobiotics is currently being developed; one strategy would be to improve mental health outcomes by administering prebiotics and probiotics [219,238]. Special attention should be paid to the consumption of foods without food additives, as there is evidence that the addition of various flavors to highly processed foods can alter the composition of the microbiota and support the obesogenic process [239].

In addition, the microbial ecosystem of the gastrointestinal system acts as a thermogenic organ and could be a potential therapeutic intervention to address energetic disorders ranging from premature growth failure to adult obesity [240].

The inability of the microbiota to convert tryptophan into indoles that activate the aryl hydrocarbon receptor leads to impaired mucosal barrier integrity and decreased GLP-1 secretion, ultimately favoring the development of the metabolic syndrome. As supplementation with a *Lactobacillus* strain alleviates the metabolic disturbance, supplementation with the natural aryl hydrocarbon receptor ligand-producing bacteria has the potential to be a novel preventive or curative treatment for metabolic syndrome [241].

### 7.2. Branched Chained Amino Acids and Obesity

Branched-chain amino acids (BCAAs)–leucine, isoleucine, and valine are essential amino acids that are not produced by the body and must therefore be obtained from food. All three BCAAs together make up about 20% of total protein and account for a third of the essential amino acids in the diet. However, bacteria that are part of the human microbiota have been found to be able to synthetase BCAAs [242]. Interestingly, there is increasing evidence that BCAAs, or branched-chain α-keto acids (BCKAs), in addition to hyperactivation of mTOR signaling, are involved in the induction of oxidative stress, mitochondrial dysfunction, apoptosis, and, more importantly, insulin resistance and/or impaired glucose metabolism, all of which are key factors in the pathogenesis of metabolic disorders [243,244,245,246,247].

The available data on the relationship between BCAAs (particularly leucine) and insulin resistance is contradictory. BCAAs have been shown to have different or even opposite effects depending on the metabolic state (predominance of either catabolic or anabolic activity) [248].

According to Cuomo et al. (2022), a low-BCAA diet is often associated with promoting metabolic health. High levels of BCAAs are associated with a number of metabolic disorders, including insulin resistance, obesity, and diabetes. Further studies in rats have shown that low consumption of BCAAs is associated with reduced insulin resistance and fat accumulation [242]. In fact, the relationship between BCAAs and insulin resistance is stronger than the relationship between insulin resistance and lipoprotein levels. Western diets containing more than 20% BCAAs are one of the major factors contributing to the increase in type 2 diabetes and obesity worldwide [242]. Metabolomics data has shown that an increase in blood levels of BCAAs can predict the onset of type 2 diabetes more than 10 years in advance [249].

On the other hand, an association has been found between high plasma leucine levels and reduced all-cause mortality [250]. In obese animals, leucine and isoleucine alleviate insulin resistance and promote the browning of white adipose tissue [251]. Some authors suggest that dietary supplementation with leucine in obesity improves mitochondrial dysfunction, reduces inflammatory processes, and decreases body weight [252,253]. As butyrate is known to improve human metabolism by increasing mitochondrial activity, reducing metabolic endotoxemia, and activating gluconeogenesis in the gut (via gene expression and/or hormone release), the beneficial effects of leucine supplementation may be explained by leucine-induced stimulation of colonic butyrate and propionate production by gut bacteria [254].

Recent data shed light on these conflicting results: leucine supplementation in situations where catabolic processes predominate improves lipid oxidation and mitochondrial function in skeletal muscle, whereas supplementation of this amino acid when the body is characterized by anabolic processes leads to BCAA accumulation with mitochondrial dysfunction and incomplete lipid oxidation [248].

## 8. Epigenetics of Obesity

Many studies suggest that obesity is the result of an interaction between the epigenome and environmental factors. Unlike genetic factors, which are fixed at birth, epigenetic modifications are dynamic and can be influenced by diet, stress, physical activity, sleep patterns, microbiota, alcohol intake, and exposure to endocrine disruptors. These changes in genes, without variation in the nucleotide sequence of genes, can be transmitted intergenerationally or even transgenerationally [255,256]. Modifications that occur during intrauterine development exhibit the greatest impact on fetal health. Simply put, low calorie intake during pregnancy induces epigenetic changes in the fetus that ensure its survival in conditions of food scarcity. However, this metabolic survival pattern results in a thrifty phenotype, which is associated with an increased risk of obesity when exposed to a hypercaloric diet later in life [257,258].

Epigenetic modifications include DNA methylation, histone modifications, and non-coding RNA-mediated regulation (ncRNAs).

1. The first process, DNA methylation, is the best studied: hypermethylation at the 5′ position of a cytosine residue, specifically at cytosine-phosphate-guanine dinucleotides (CpG) in promoter regions, is generally associated with transcriptional repression, whereas hypomethylation and intragenic methylation are associated with gene activation [257,258,259,260]. Since a large number of DNA methylations have been described in obese patients, from a practical point of view, it is necessary to identify those specific gene changes that affect the transcriptional network responsible for body weight regulation and are consistently associated with weight gain [257]. For example, DNA methylation of the adiponectin gene in subcutaneous adipose tissue or the POMC gene at the neuronal level has been shown to correlate strongly with body mass index [259]. Interestingly, epigenetic changes are in principle a consequence of weight gain and not a determinant, as previously thought, according to an analysis of a group of 5387 people [258]. To date, DNA methylation in some genes has been shown to be associated with appetite control, insulin signaling, and metabolic processes.

In this context, we envisage that pharmacologically induced epigenetic modulation will be of interest in the future. This can be achieved by influencing the activity of DNA methyltransferases (DNMTs) involved in methylation and the ten-eleven translocation (TET) proteins that reverse this process [259,261,262]. In fact, some DNMT inhibitors, 5-azacytidine, and 5-aza-20-deoxycytidine (decitabine), approved for certain hematological malignancies, are being reconsidered in the light of methylation blockade [263]. Animal studies have already shown that these substances are modulators of adipogenesis, with distinct effects at different stages [264]. There is also renewed interest in metformin, which, by activating AMP-activated protein kinase (AMPK), facilitates the phosphorylation (and subsequent inhibition) of enzymes such as DNMTs, histone acetyltransferases (HATs), histone methyltransferases (HMTs), and class II histone deacetylases (HDACs) [265]. In addition, SAM supplementation, which is a universal methyl donor to both DNMTs and HMTs, can serve as a therapeutic intervention for obesity. Other supplements, such as resveratrol, nicotinamide riboside, genistein, epigallocatechin 3-gallate, and curcumin, have also proven promising results in experimental studies [259,266].

In terms of the effect of different diets on epigenetic changes, at one end of the spectrum are hypercaloric regimens, which modulate the methylome depending on their composition. Ling’s study showed that supplementation with PUFA (750 kcal/day) for 7 weeks increased methylation processes for 1797 genes, including IL6, insulin receptor, neuronal growth regulator 1 (NEGR1), and POMC. On the other hand, in the same study, the saturated fatty acid (SFA) overfed group was associated with methylation of only 125 genes (of which as few as 47 were common to both PUFA and SFA groups). Analysis of the data highlighted that correlations between DNA methylation and mRNA levels (gene expression) were only evident in the SFA-supplemented group, despite similar weight gain in the two groups [267]. Interestingly, several papers have suggested that DNA methylation of mitogen-activated protein kinase 7 (MAPK7), melanin-concentrating hormone receptor 1 (MCHR1), splicing factor SWAP homolog (SFRS8) in adipose tissue, and NPY and POMC in leukocytes could be used as markers to predict weight gain [267].

On the other pole, hypocaloric diets also induce epigenetic changes in genes such as ATP10A, AQP9, CD44, DUSP22, HIPK3, TNNT1, and TNNI3, to name just a few. The methylation of these genes can be used as biological markers that certify therapeutic success in obesity [258]. Worryingly, it is worth noting that the epigenome is more susceptible to changes with exposure to hypercaloric diets than it is with calorie restriction.

2. Histones are proteins around which DNA is wrapped, forming a structure called chromatin. Regarding histone modifications, numerous phosphorylation, methylation, acetylation, adenosine diphosphate (ADP) ribosylation, and carbonylation processes have been identified in the amino-terminal tails of histones in obese patients [257,258]. Of particular interest is the acetylation of PPARγ2 (a key regulator of important transcriptional pathways) during adipogenesis. Through chromatin remodeling, these histone modifications have effects such as increasing the expression of some genes or silencing others (acetylation catalyzed by histone acetyltransferases (HATs) favors transcription, whereas deacetylation catalyzed by HDACs is associated with suppression).

Murine studies have shown that histone carbonylation in adipose tissue can be considered a redox-related epigenetic marker, confirming the strong link between obesity, oxidative stress, and chronic inflammation. Remarkably, clinical studies have shown that insulin resistance correlates with reduced histone modifications in adipose tissue. These data have been confirmed by cell culture studies showing that this type of epigenetic change is mediated by the nicotine adenine dinucleotide (+)−dependent HDACs (SIRT family) and occurs 3 days after induction of insulin resistance with palmitic acid [268,269].

Sodium butyrate, an HDAC inhibitor, has been reported to reduce high-fat diet-induced obesity [259].

3. NcRNA molecules, such as microRNAs (miRNAs), long non-coding RNAs (lncRNAs), and circular RNAs (circRNAs), do not encode proteins but play important regulatory roles in gene expression. Extremely interesting data suggest that adipose tissue cells produce miRNAs that are encapsulated in small extracellular vesicles and then released into circulation [270]. Adipose tissue-derived miRNAs (miR-31, miR-519d, mi-R222, miR-130b, miR-15b, miR-125b, miR-122, and miR-142), which are considered novel adipokines, can be used as biological markers of weight gain. Additionally, recent data validates the traditional concept of milk siblings. Breast milk contains abundant amounts of miRNA-148a, miR-152, miR-29b, and miR-21, which confer epigenetic similarities between babies who have consumed milk from the same mother [271].

It is also of practical importance to know that obesity plays a role in reprofiling the type of miRNA released into the circulation, thus influencing insulin resistance, inflammatory processes, and the tumor microenvironment.

Furthermore, miRNAs play important roles in gene regulation (by cleaving mRNA or inhibiting protein translation), making them extremely valuable therapeutic targets. One such example could be targeting miRNAs that affect NO synthase and Sirtuin 1, with a potential beneficial effect on ameliorating the oxidative stress and endothelial dysfunction seen in obesity.

One future prospect is to repurpose compounds that are already in advanced stages of clinical evaluation for the treatment of obesity. For example, MRX34, a liposomal miR-34a, may have a beneficial effect in the treatment of obesity. However, the phase I clinical trial of this compound as an antineoplastic therapy failed due to severe immune side effects. Miravirsen, an antisense oligonucleotide drug inhibitor of miR-122 (phase II for hepatitis C), is also of potential interest for use in patients with hepatic steatosis and obesity [272].

circRNAs are known to compete with miRNA/mRNA binding and interfere with their functions. CircRNAs are reported to regulate the differentiation of adipocytes [259]. Similar functions have also been described for long non-coding RNAs (lncRNAs) [260].

Knowledge of fetal programming is an important preventive tool. Optimizing the mother’s diet and providing a safe environment during pregnancy can play a prophylactic role in combating obesity in (at least) the next two generations [255]. Perhaps unsurprisingly, children born after maternal bariatric surgery have a different epigenetic inheritance compared to children born before the intervention [260]. On the other hand, in the adult population, understanding the epigenetic patterns that favor the upregulation of obesity-promoting genes and discovering the methods to reverse the obesity-associated methylome (association of specific diets, nutraceuticals, exercise, and new therapeutic agents) open the way to personalized epigenetic medicine [257]. There is also an urgent need to use specific biological markers to identify at-risk populations—individuals who are resistant to weight loss and prone to rapid weight regain.

## 9. Pharmacotherapy of Obesity

Obesity, a chronic disease with significant complications in all age groups, has reached pandemic proportions. In Europe, obesity is the fourth most common risk factor for developing a non-communicable disease and has been associated with increased cardiac mortality, a higher risk of thirteen types of cancer, and reduced quality of life [119].

In this complicated puzzle represented by the pathology of obesity, it is unlikely that only one drug will be the silver bullet that represents a complete solution to such a large problem. Therefore, the best therapeutic results are obtained by combinations of drugs that also allow for lowering the doses and decreasing the side effects. Current medication includes four drugs approved by the FDA for short-term use (phentermine, phendimetrazine, diethylpropion, and benzphetamine) and six medications (orlistat, phentermine-topiramate, naltrexone-bupropion, liraglutide, semaglutide, and setmelanotide) for long-term use [273]. Established guidelines advise that drug treatment should be stopped or modified if at least 5% weight loss is not achieved after 12 weeks of drug treatment [274].

However, given that weight loss leads to a significant reduction in the chronic complications of obesity, the number of drugs currently in use is extremely small. Even in this context, recent data indicate that anti-obesity medications are underused, with only 2% of the obese population taking them [19,274]. There are many possible reasons for this: insufficient knowledge about these drugs, low accessibility due to high prices, low adherence due to side effects, the modest weight loss that these drugs can achieve, the poor reputation of drugs withdrawn from the market due to poor safety profiles, and the stigma associated with obesity that reduces access to health-care services [119].

A detailed understanding of the pathophysiological mechanisms underlying the onset of obesity has led to the discovery of new drugs and therapeutic strategies with the potential to alleviate this disease. Although a wide range of drugs are already used in obesity therapy, extensive preclinical and clinical ongoing studies aim to discover and bring to market new weight-loss drugs. It is hoped that the new molecules will bring benefits in terms of fewer side effects, greater weight loss, longer periods of optimal weight maintenance, and a lower cost/benefit ratio compared to current anti-obesity medication.

The increased interest in this area of research is reflected in the plethora of non-invasive approaches for treating obesity, including drugs that target numerous pathophysiological pathways (from the central nervous system, gastrointestinal tract, adipose tissue, kidney, liver, and skeletal muscle) but also modern delivery systems, gene therapy, and vaccines [275].

A synthetic overview of the pharmacotherapy for obesity from its beginnings to date is presented in Table 2.

A comprehensive review of obesity-promoting factors should include drugs that cause significant weight gain, such as glucocorticoids, antipsychotics (clozapine and olanzapine), antidepressants (tricyclic antidepressants and paroxetine belonging to selective serotonin reuptake inhibitors), antidiabetics (sulfonylureas and thiazolidinediones), antiepileptics (valproate), antihypertensives (beta-blockers), and oral contraceptives, to name but a few [432,433]. Fortunately, drugs have a variable risk of associated weight gain, with significant variation within classes; this characteristic allows switching from a molecule associated with significant weight gain to one with a lesser effect on BMI [434].

Despite advances in understanding the multifaceted nature of obesity, effective interventions that promote sustained weight loss and prevent weight regain remain elusive. Behavioral changes play a pivotal role in obesity prevention and management. However, achieving long-term adherence to healthy behaviors remains a significant challenge [435]. Further research is needed to identify novel strategies that can motivate individuals to adopt and maintain healthy lifestyles. Investigating personalized approaches, such as tailored dietary recommendations, physical activity programs, and psychological interventions, can optimize behavioral changes and enhance their sustainability. Exploring new targets and pathways, leveraging emerging technologies, and conducting rigorous clinical trials can lead to the discovery of novel pharmacological interventions. Additionally, investigating combination therapies and personalized medicine approaches may optimize treatment outcomes by tailoring interventions to individual characteristics and underlying biological mechanisms. Research should focus on identifying the most effective combinations, dosage regimens, and treatment durations to maximize weight loss, minimize adverse effects, and prevent weight regain.

Addressing the challenges in obesity prevention and treatment requires multidisciplinary collaborations and comprehensive investigations. Researchers, clinicians, policymakers, and industry stakeholders must join forces to exchange knowledge, share resources, and foster innovation. Large-scale epidemiological studies, clinical trials, and translational research should be conducted to generate robust evidence that informs clinical practice and policy development. Furthermore, promoting data sharing and standardization of research protocols will facilitate the comparison and integration of findings, accelerating progress in the field.

The one-size-fits-all approach to obesity prevention and treatment has yielded limited success, necessitating a paradigm shift toward personalized medicine [436]. This article underscores the importance of considering individual genetics and lifestyle factors in obesity management. By recognizing the inherent interplay between an individual’s genetic makeup, environmental exposures, and lifestyle choices, we can unlock a new era of precision medicine that optimizes treatment outcomes and promotes sustainable weight loss. Advancements in genetic research have unraveled the intricate role of genetics in obesity susceptibility and response to interventions. Personalized medicine harnesses this knowledge by integrating genetic information into clinical decision-making. Genetic profiling can identify individuals at higher risk of obesity, allowing for targeted prevention strategies tailored to their specific needs. Additionally, understanding genetic variations that influence drug metabolism and response can aid in the selection of pharmacological interventions, optimizing treatment outcomes, and minimizing adverse effects [437]. While genetics lay the foundation, lifestyle factors are equally crucial in the development and management of obesity. Personalized medicine recognizes this intricate interplay by considering an individual’s unique circumstances, such as dietary preferences, physical activity levels, socioeconomic factors, and psychosocial aspects.

Digital health technologies, such as wearable devices, mobile applications, and remote monitoring systems, offer unprecedented opportunities for personalized obesity management. These technologies provide real-time data on physical activity, dietary patterns, sleep quality, and other relevant metrics. By integrating this data with genetic information, healthcare providers can develop personalized intervention plans, offer real-time feedback, and facilitate remote coaching, ultimately empowering individuals to take control of their health and make informed choices.

Implementing personalized medicine approaches in obesity management does come with challenges. Ensuring accessibility and affordability of genetic testing, addressing privacy concerns, and promoting health equity are critical considerations. Additionally, the ethical implications of genetic information and its potential impact on individuals’ psychosocial well-being must be carefully addressed [438]. Collaborative efforts among healthcare professionals, policymakers, researchers, and ethicists are essential to navigate these challenges and ensure the responsible and equitable implementation of personalized medicine in obesity care. To harness the full potential of personalized medicine in obesity prevention and treatment, the translation of research findings into clinical practice is paramount.

## 10. Bariatric Surgery

Obesity is a widespread health problem with a major impact on the health and general well-being of the population and massive healthcare costs. Estimates by the World Obesity Federation of global obesity rates by 2030 are grim. More than one billion people are expected to be obese. One in five women and one in seven men will be affected [439]. As the prevalence of obesity continues to rise, especially in low- and middle-income countries, some experts in the field consider bariatric surgery as a possible solution to the coming obesity pandemic [440].

Since its introduction, bariatric surgery has been accepted and practiced worldwide and has proven to be one of the most effective methods of achieving sustained and reliable weight loss. Numerous studies have shown improved outcomes in all population groups that have undergone surgery compared with other methods, such as pharmacological approaches or lifestyle and dietary changes [441]. The improved outcomes are not only in terms of weight loss but also in terms of improvement and even remission of the most common comorbidities associated with obesity (hypertension, T2D, dyslipidemia, and sleep apnea) [442]. These surgical procedures, also known as metabolic surgery because of their profound effect on the anatomy and physiology of the digestive tract, are based on the principles of restriction, malabsorption, or a combination of the two [443]. The two most commonly performed procedures worldwide are longitudinal sleeve gastrectomy (LSG) and Roux-en-Y gastric bypass (RYGB). In addition to reducing excess body weight, long-term control of all metabolic comorbidities is equally important [444].

Bariatric surgery can now be safely considered for appropriately selected adolescents and patients over the age of 70, as several randomized trials and meta-analyses have demonstrated long-term safety and efficacy. As of November 2022, more than 20 years after the first recommendations for bariatric surgery, the American Society of Metabolic and Bariatric Surgery (ASMBS) and the International Federation for the Surgery of Obesity and Metabolic Disorders (IFSO) have updated the criteria for bariatric surgery and lowered the threshold as follows:

- Patients with a BMI >35 kg/m^2^ with or without comorbidities and who do not present a high anesthetic surgical risk;
- Patients with a BMI of 30–34.9 kg/m^2^ with one or more obesity-related comorbidities or with significant impairment of quality of life (T2D, essential hypertension, dyslipidemia, sleep apnea, or non-alcoholic fatty liver disease) [445].

Most studies show very good and consistent weight loss at 12 months after surgery. For both LSG and RYGB, the percentage of excess body weight loss is over 60% [446]. Although good results are consistent for the vast majority of patients, bariatric surgery is not without risk. In addition to the usual complications common to most laparoscopic surgeries, patients who have undergone bariatric surgery need to be closely monitored both clinically and nutritionally [447].

Micronutrient deficiencies can be a common problem following metabolic surgery, and many patients may require routine vitamin and mineral supplementation [448]. These deficiencies are usually explained by reduced dietary intake and anatomical and physiological changes in the gastrointestinal tract, particularly in the case of malabsorptive procedures [449,450,451]. Vitamin B12, which is only available exogenously, must be carefully monitored and supplemented. Unmonitored patients may develop anemia due to altered gastrointestinal absorption. Micronutrient deficiencies are usually more severe after RYGB than after LSG.

Perhaps one of the most spectacular improvements after bariatric surgery is the remission of T2D. The beneficial effects on glucose metabolism have been extensively studied. Criteria for complete remission of T2D include fasting plasma glucose <100 mg/dL and/or HbA1c <6% for at least 1 year after surgery in the absence of glucose-lowering pharmacological treatment. Complete remission for 5 or more years is considered curative [452]. Most randomized clinical trials report remission rates of 60–80% 2 years after surgery. There is still a risk of T2D recurrence in the postoperative period, but glycemic control remains satisfactory and insulin resistance has improved [453]. Most studies tend to focus on biomedical aspects such as weight loss and clinical and biological improvement of comorbidities, but improvement in quality of life (QoL) is equally important, especially as the number of procedures performed worldwide continues to increase [454]. QoL is severely impaired in obese patients and is directly proportional to BMI. In addition to weight loss, bariatric surgery has been shown to improve several aspects of QoL, including patient satisfaction, self-esteem, body image, and physical and social functioning [441,455,456].

Obesity is a complex and chronic disease with multiple and incompletely elucidated mechanisms. After more than half a century of study and research, the ideal solution remains elusive. Complete treatment cannot be achieved by bariatric surgery alone. Patients still need long-term nutritional and psychological counseling. The best results are achieved by an informed patient with realistic expectations, a well-trained surgeon, and a multidisciplinary team.

## 11. Implementation Gap and Public Health Strategies

**Figure 1:**
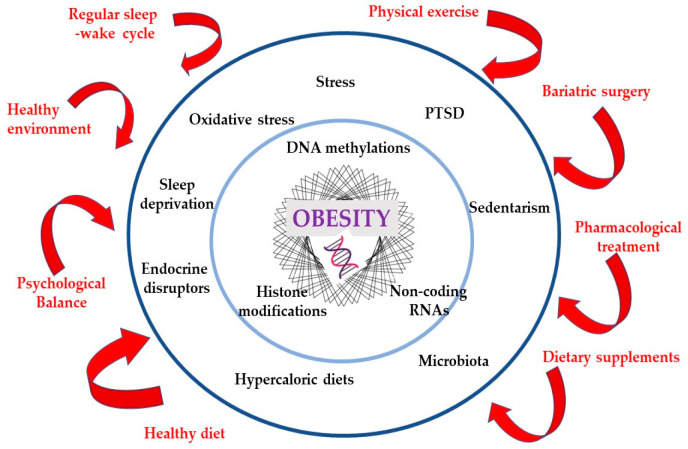
Key players at the “table” of obesity.

Despite significant progress in understanding the causes and contributing factors of obesity, the implementation of effective interventions has been disappointingly slow. Barriers to translating scientific evidence into practical solutions range from systemic issues, such as limited funding and resource allocation, to societal factors, such as cultural norms and social inequalities. The prevailing societal norms that promote sedentary lifestyles and unhealthy food environments contribute to an obesogenic environment (Figure 1). Moreover, cultural practices, traditions, and beliefs about food, body image, and physical activity influence individual behaviors and can pose challenges to the adoption of healthier habits [457,458]. Addressing these cultural and societal influences requires targeted and culturally sensitive interventions that respect and take into account different perspectives and values. Negative attitudes, stigma, and discrimination towards individuals with obesity are significant barriers that impede access to care, social support, and positive health-seeking behaviors. In addition, psychological factors such as stress, depression, and low self-esteem can undermine motivation and hinder sustained behavior change efforts [459].

Furthermore, the complexity of obesity demands a comprehensive and integrated approach across multiple sectors, including healthcare, education, urban planning, and food policy. The fragmented nature of these sectors hinders coordinated efforts and impedes the translation of knowledge into practical action [460]. The lack of coordinated efforts among stakeholders, including policymakers, healthcare providers, and community organizations, has also hindered progress.

Policy interventions have proven to be effective in reshaping environments and promoting healthier choices. Examples include implementing sugar-sweetened beverage taxes, food and nutrition labeling regulations, and restrictions on the marketing of unhealthy foods and beverages to children. These policy changes have the potential to influence consumer behaviors, reduce the use of unhealthy products, and encourage the adoption of healthier alternatives. Creating supportive environments that facilitate physical activity and healthy eating is crucial for obesity prevention. Promoting walkable communities, building bike lanes, and establishing parks and recreational facilities can encourage regular physical activity. Additionally, improving access to affordable, nutritious foods through initiatives such as farmers’ markets, urban gardens, and healthy food retail programs can enhance healthy eating behaviors [461].

Education plays a pivotal role in empowering individuals with knowledge and skills to make informed choices regarding their health. School-based interventions that incorporate nutrition education, physical activity programs, and healthy lifestyle curricula have shown positive outcomes in preventing obesity among children. Similarly, workplace wellness programs that offer health education, physical activity opportunities, and healthy food options can contribute to obesity prevention among adults. Engaging communities in obesity prevention efforts foster social support, promotes collective action, and addresses local needs and priorities. Community-based programs, such as family-focused interventions, peer support groups, and community gardens, have shown promise in promoting healthy behaviors and reducing obesity rates. By involving community members in the planning and implementation of interventions, these initiatives can have a lasting impact on obesity prevention [461,462].

Preventive measures for children are particularly important because of the long-term health effects of obesity in early life. Promoting breastfeeding, implementing nutrition standards in schools, and restricting the marketing of unhealthy foods to children are effective strategies that can shape healthier behaviors from an early age. By investing in comprehensive early intervention programs, we can build a strong foundation for lifelong health and reduce the risk of obesity-related complications [463].

Addressing obesity in adults requires a multifaceted approach that combines policy changes, community engagement, and individual empowerment. Implementing workplace wellness programs, offering nutrition and physical activity counseling, and providing access to evidence-based weight management programs are effective strategies to support adults in their weight management journey. By equipping adults with the necessary tools and resources, we can improve health outcomes and reduce the burden of obesity-related chronic diseases. The obesity pandemic demands urgent and concerted efforts to implement effective preventive measures. By leveraging policy changes, environmental modifications, educational initiatives, and community engagement, we can make substantial progress in curbing the obesity crisis. These examples of successful interventions highlight the potential impact of evidence-based strategies for both children and adults. By scaling up these initiatives and tailoring them to local contexts, we can create supportive environments, empower individuals, and ultimately reduce the burden of obesity, leading to a healthier future for generations to come.

## 12. Conclusions

The global incidence of obesity has increased dramatically since 1975, making it the leading lifestyle-related risk factor for premature death. To address this problem, WHO has proposed a Global Action Plan for the Prevention and Control of Noncommunicable Diseases 2013–2020 [1,2]. Tackling obesity is key to achieving the Sustainable Development Goals and is a priority in the European Work Programme 2020–2025: United Action for Better Health [119]. While progress has been made in identifying the causes and contributing factors of obesity, the implementation of effective interventions has been slow [3,4]. There is a need to translate knowledge into action. Prevention, public health education, and policy interventions are critical strategies to address the obesity pandemic, given the role of gene-environment interactions in human health and disease. Epigenetics may help explain why genetics alone cannot explain the prevalence of obesity [6]. Overall, a comprehensive understanding of mechanisms and the translation of research findings into clinical practice are essential for the development of effective strategies to combat the growing obesity epidemic and its chronic complications. Further research is needed to identify effective behavioral changes and develop safer and more effective medications. Preventive measures, targeting both children and adults, are critical to reversing the obesity epidemic. In the future, personalized medicine approaches that take into account individual genetics and lifestyle factors may hold promise for more effective prevention and treatment of obesity.

In conclusion, the growing obesity epidemic demands our immediate attention and calls for collective action. The staggering prevalence of obesity, coupled with its associated chronic complications, presents a formidable challenge that cannot be ignored. It is imperative that researchers, healthcare professionals, policymakers, and the public come together in a unified front to tackle this pressing issue head-on.

## Figures and Tables

**Table 1:** Pros and cons of various animal models of obesity. Adapted from [23].

	Model	Species	Examples	Pros	Cc
1	Monogenic	Mice and rats	ob/ob mice db/sb mice s/s mice B6 (cg)–Tubtub/J [22] Zucker fatty rat [22] Otsuka Long-Evans fatty rat	Dependable and practical Quick age development Inexpensive Controlled reproduction in high numbers Ability to perform low-density lipoprotein experiments (LDL) by controlling environmental factors Well-defined molecular marker map	Requires technical knowledge Fat storage and energy usage are different from that of humans Not equivalent to a human disease
2	Polygenic	Mice	New Zealand obese mice [22] Tsumura and Suzuki obesity and diabetes mice [22] Kuo Kondo-Ay mice M16 mouse	Easy technique Often used animal model Inexpensive and practical model Relatable to obesity in humans	Requires technical knowledge
3	Transgenic and Knockout	Mice	Transgenic Corticotropin-Releasing Factor-Overexpressing Mice Melanin-concentrating hormone overexpressing (MCH-OE) mice Overexpression of 11β-hydroxysteroid dehydrogenase type 1 (11β HSD1) mouse [22] Overexpression of glucose transporter subtype 4 [22] UCP-DTA mouse Knockout Beta-3 adrenergic receptor knockout mice Bombesin receptor subtype 3 knockouts Deletion of the neural insulin receptor (NIRKO) mouse Disruption of the Neuropeptide-Y 1 receptor (NPY1R) mice Knockout of the Serotonin 5-HT-2C Receptor Gene Neuropeptide Y Receptor Y2 (NPY2R) knockout mice	Dependable and practical Genetic tools available; genetic targeting is possible	Requires technical knowledge
4	Diet induced	Rats and mice	High carbohydrate-fed (HCD) Diet-induced obese cafeteria (CAF) Maternal overfeeding and exposure to high-fat diets Zucker fatty rat	Covers both hereditary and diet-related factors Rapidly occurring obesity and lack of insulin response Extremely comparable to obesity in humans Inexpensive Covers most elements of human metabolic syndrome X	Technical standards not sufficiently regulated Not time efficient Overly obese animal models may occur
5	Unfamiliar	Bats and seals	Dietary	These models go through seasonal fat storage cycles	The genetic basis has not yet been established Cannot develop in-house colonies
6	Non-human primates	Macaques, baboons, rhesus monkeys	Dietary	Very similar from an anatomical and physiological point of view. It is relevant in terms of translation. Blood sampling endoscopy and laparoscopy biopsies are possible	Expensive to maintain Approved facilities are limited Long life cycle and animals are uniparous
7	Seasonal	Hamsters	Syrian hamster Siberian hamster	Photoperiodism	Poor standardized animal model
8	Non-mammalian	Fish except for zebrafish	Dietary	Quick lifecycle Inexpensive	Unique anatomical and physiological features
9	Sizeable animals	Cats, dogs, and pigs	Dietary	Closely related to humans from a genetic standpoint Possibility of performing cannulations Closely related to humans from a pharmacokinetic point of view New model	Highly complex Specially designed laboratories are a must Long life cycle Little information available about this animal model
10	Chemical or Surgical	Rats	Lesions of the ventromedial hypothalamus (Monosodium glutamate; Electrical ventromedial hypothalamus lesion) Damage of the hypothalamic paraventricular nucleus Ovariectomy	Highly dependable and practical Avoiding using hazardous chemicals on organisms	Technical expertise Surgical protocols required High difficulty in locating VMH, PVN, and ARC of the brain Post-surgical protocols required High mortality is to be expected

**Table 2:** Overview of pharmacotherapy for obesity.

Targeted Pathway	Pathophysiologic Basis	Drug	Side Effects/Observations
Polypharmacy	Combined ampheta-mines, diuretics, thyroid hormones	Agent type: energy intake and expenditure Rainbow pills approved in 1941–1968 (FDA) [273]	Insomnia, palpitations, anxiety, increase in heart rate and blood pressure, death [276]
Sympathicomimetic	Release of norepi-nephrine, dopamine, and serotonin at the nerve terminals Inhibition of the reuptake of monoamines, increasing their synaptic levels [277]	Agent type: energy intake and expenditure Methamphetamine approved in 1947–1979 (FDA)	High risk for abusiveness and addiction [276]
Sympathicomimetic	Amphetamine con geners Direct α-adrenergic agonism Indirect stimulation of norepinephrine release, similar to ephedrine’s effect [278]	Agent type: energy intake and expenditure Phenylpropanolamine approved in 1976–2000 (FDA) [273]	Hemorrhagic stroke [276]
Sympathicomimetic	Phenylalkylamine sympathomimetic, similar to amphetamine [279]	Agent type: energy intake and expenditure Phenmetrazine (active compound) 1956–present (FDA) Phendimetrazine * (prodrug) 1959–present (FDA) for short term use	Nausea, diarrhea, dry mouth [276,280]
Sympathicomimetic	Amphetamine congeners [273]	Agent type: energy intake and expenditure Diethylpropion/am-fepramone * was approved in 1959 (FDA) for short-term use, withdrawn in 2022 (EMA) [281]	Pulmonary hypertension, psychiatric disorders [276,282,283]
Sympathicomimetic	Amphetamine congeners [273]	Agent type: energy intake and expenditure Benzphetamine * approved in 1960 (FDA) for short term use	Agitation, anxiety, confusion, dizziness, fast heartbeat
Sympathicomimetic	α1-adrenergic agonist Modulates neuronal activity in the nucleus accumbens shell, acting on dopamine D1 and D2 receptors [284]	Cathine (D-norpseudoephedrine) approved in 1975 (EMA) for short- term use, ≤12 weeks [276]	Tachycardia, increase in blood pressure, restlessness, sleep disorder, and depression [276,285]
Sympathicomimetic	A norepinephrine and serotonin reuptake inhibitor	Agent type: energy intake Sibutramine 1997–2010 (FDA, EMA)	Headache, insomnia, dry mouth, constipation, non- fatal myocardial infarction, and stroke (in individuals with pre- existing cardio-vascular diseases) [276,286]
Sympathicomimetic	Amphetamine congeners [273,287]	Agent type: energy intake and expenditure Phentermine * 1959–present (FDA) for short-term use	Dry mouth, insomnia, palpitations, tachycardia, hypertension, anxiety, dizziness, and constipation [274,276,286]
Sympathomimetic/anticonvulsant	Phentermine in creases mainly norepinephrine in the hypothalamus [275] Topiramate blocks voltage dependent sodium channels, glutamate receptors, and carbonic anhydrase, and augments GABA activity, promotes taste aversion, decreases caloric intake, and stimulates lipolysis [287]	Agent type: energy intake Phentermine/topiramate * approved in 2012 (FDA) for adults with BMI ≥ 30 kg/m^2^ or BMI ≥ 27 kg/m^2^ with at least one weight-related comorbidity but refused twice by EMA [288]	Elevation in heart rate, mood and sleep disorders, cognitive impairment, depression, suicidal ideation, metabolic acidosis, paresthesia, dry mouth An increased risk of cleft lip/palate in infants with exposure during the first trimester of pregnancy [274,289,290]
Sympathomimetic	ß3-adrenergic recep-tor agonist, which primarily targets the brown adipose tissue [291]	Agent type: energy expenditure Mirabegron approved in 2012 (FDA) for treating incontinence, phase II for obesity [292]	Hypertension (most com monly), nasopharyngitis, and urinary tract infection [276,293,294,295]
Combinations Targeting the Neurotransmitters and Neuropeptides	Bupropion is a reuptake inhibitor of dopamine and norepinephrine that promotes activation of the central melanocortin pathways Naltrexone is a com petitive MOR and DOR antagonist that exhibits synergic effects with bupropion (increases the release of anorexigenic melanocortins such as α-MSH and β-MSH Naltrexone diminishes the feedback inhibition caused by beta-endorphins which are known to stimulate food intake) [287,288,296]	Agent type: energy intake Bupropion/Naltrexone * 2014 (FDA), 2015 (EMA) [288]–indicated for long term use The combination of bu-propion with zonisamide was also investigated (phase II) [297]	Nausea, constipation, head ache, vomiting, dizziness, insomnia, dry mouth, seizures, and palpitations [273,276,287,289] Caution in patients treated with antidepressants and some antipsychotics [274]
Combinations targeting the Neurotransmitters	Multimode inhibitor of norepinephrine, serotonin, and dopamine reuptake/beta1 blocker	Agent type: energy intake Tesofensine/metoprolol (phase IIb–for hypothalamic obesity) [290]	Increased heart rate for tesofensine [298,299]
Targeting Dopamine pathway	Dopamine reuptake in hibitor Increases brain synap-tic dopamine action on the nucleus accumbens, and striatum [300]	Agent type: energy intake and expenditure Methylphenidate (phase III) [289,301] (approved by FDA for the treatment of attention-deficit/hyperactivity disorder or narcolepsy)	Irritability and insomnia [302]
Targeting Serotonin pathway	Stimulates the release of serotonin and inhibits its reuptake in the synaptic cleft	Agent type: energy intake Fenfluramine 1973–1997 (FDA) Fenfluramine associated with phentermine (never approved by FDA) Dexfenfluramine 1996–1997 (FDA)	Valvular regurgitation in sufficiency (direct stimulation of 5- H T2B receptors on the interstitial cells of the mitral and aortic valves), primary pulmonary hypertension, and cardiac fibrosis [273,303,304]
Targeting Serotonin pathway	Suppression of NPY/AgRP neurons Stimulatation of POMC/CART neurons Activation of melanocortin 4 receptor pathway [305]	Agent type: energy intake 5-HT2C receptor agonist Lorcaserin 2012–2020 (FDA), never approved by the EMA [287,306,307].	Increased incidence of certain cancers
Cannabinoids pathway	Decreases appetite, enhances thermogenesis, and diminish lipogenesis Cannabidiol activates the endocannabinoid system, 5HT-1A receptors, PPAR- γ and inhibits anandamide reuptake [308]	Agent type: energy intake CB1 R inverse agonist Rimonabant never approved by FDA, 2006–2008 EMA [290] Taranabant (discontinued) AM251 (preclinical) [309] Peripheral CB1 R inverse agonist AM6545 [275] JD5037 (phase I for Nonalcoholic steatohepatitis) [310,311] FB-024 for kidney disease [273] CB2 R antagonist JWH-015 (preclinical) Cannabidiol solution RAD011 (phase III–for Prader-Willi syndrome) [308,312]	Hepatic abnormalities, diarrhea, fatigue, vomiting, and somnolence (cannabidiol) [313] Warnings regarding drug-drug interactions [313]
Entero-endocrine pathway Incretin mimetics Glucagon-like peptide-1 (GLP-1)	Decreases appetite through direct activation of POMC/CART neurons [273,276] and suppression of AgRP/NPY neurons through GABA-dependent signaling [273,276]	Agent type: energy intake GLP-1 agonists/analogs Human GLP-1 backbone: Liraglutide * approved in 2014 (FDA), 2015 (EMA) for the treatment of adult obesity; approved in 2020 (FDA), 2021 (EMA) for the treatment of obesity in adolescents aged 12–17 years [314] Semaglutide * approved in 2014 (FDA), 2015 (EMA) for adults with BMI ≥ 30 kg/m^2^ or BMI ≥27 kg/m^2^ with at least one weight-related comorbidity), approved in 2021 (FDA), 2022 (EMA) for teens ages 12 and up who have a BMI at or above the 95th percentile for their age and sex [315,316]. Dulaglutide (registered for the treatment of T2D) but exhibiting weight reducing effects [294,317] Exendin-4 backbone: Exenatide (registered for the treatment of T2D) but exhibiting weight reducing effects [294] Lixisenatide Pipeline drugs: Efpeglenatide (phase III) Rybelsus (phase III) Danuglipron (PF-06882961) (phase II) GLPR- NPA (phase I) PF-07081532 (phase I) [276,318] Noiiglutide (SHR20004) (phase II) [319] LY3502970 (phase II) [320] XW003 (phase II) [289,321] XW004 (phase I) [273]	Increased heart rate, hypo glycemia, constipation, diarrhea, nausea, vomiting, headache, reversible increases in amylase/lipase activity (liraglutide) [287,289,322] Nausea, vomiting, diarrhea, abdominal pain, constipation, and headache (semaglutide) [289] Warnings about personal or family history of medullary thyroid carcinoma or in patients with multiple endocrine neoplasia syndrome type 2, pregnancy [289] Semaglutide induces greater weight loss compared to currently FDA-approved drugs (of up to 15–30% of baseline body weight as compared to 5–10%), which opens a new era in the pharmacotherapy of obesity [273,323]
Entero-endocrine pathway–Incretin mimetics	Increases insulin production and decreases hepatic glucose overproduction	Agent type: energy intake DPP-4 inhibitor Yogliptin (phase III for T2D) [273]	Potentially reduced risk for acute pancreatitis described for drugs found already in use for the treatment of T2D [324]
Entero-endocrine pathway–Incretin mimetics Glucose- dependent insulinotropic polypeptide (GIP) pathway–also known as gastric inhibitory polypeptide [325]	Postprandial potentiation of insulin secretion Activation and blocking of the GIPR receptor have both been shown to decrease body weight GIP regulates energy metabolism via CNS GIPR signaling [276,325]	Agent type: energy intake GIPR agonists GIPR agonist long acting (phase I for T2D) ZP 6590 (preclinical for obesity) Antagonistic GIPR antibodies [325,326,327] GIP Receptor Antagonist SKL-14959 [328]	GIP Receptor Agonism Attenuates GLP-1 Receptor Agonist-Induced Nausea and Emesis [329,330]
Pancreatic hormones pathway Glucagon	Increases blood glu-cose levels [331] Stimulates lipolysis and thermogenesis in brown adipose tissue Satiety via mediated via the liver-vagus-hypothalamus axis [276] The thermogenic effect is determined by the feeding status [276,332]	Agent type: energy intake Glucagon Analog HM15136 (phase I) [276] NN9030/NNC9204–0530 [273] Non-peptide glucagon receptor antagonist Bay 27–9955, LY2409021–discontinued [333]	Not known to date
Entero-endocrine pathway (twincretins) GIP/GLP-1	Mimics the actions of native GIP at the GIP Diminishes GLP-1 re-ceptor internalization Acts on arcuate nu-cleus and other hypothalamic regions, parietal cortex, insula, putamen, orbitofrontal cortex, adipose tissue, and gastrointestinal tract [289,334]	Agent type: energy intake GIP/GLP-1 dual agonist Tirzepatide * 2022 (FDA) [294,335,336] GIP/GLP peptide I (phase I–for T2D) GIP/GLP peptide II (phase I–for T2D) SCO-094 (phase I) [331] NN9709, formerly MAR709 and RG7697 (phase II–stopped) [276,331,337] CT-868 (phase II) AMG133 (phase I) [273] GMA106 (phase I) [338] CT-388 (phase I) [289,339]	Gastrointestinal side effects: nausea, diarrhea, vomiting, mild hypoglycemia [289,336,340] Caution for people with a personal or family history of medullary thyroid carcinoma. Patients with a history of multiple endocrine neoplasia type 2 (MEN 2) [294]
Pancreatic-entero-endocrine pathway GLP1/glucagon	Mimics the effects of GLP-1 and glucagon receptor activation [289]	Agent type: energy intake GLP1/glucagon dual agonists Cotadutide (MEDI0382) (phase IIb) BI 456,906 (phase II) Efinopegdutide (JNJ-64565111/HM12525A/MK6024 (phase IIa) Mazdutide (IBI362/LY3305677) (phase III) [289,331,341,342] JNJ-54728518 (phase I) [331] NN9277/NNC9204–1177 (phase I–stopped) CT-868 (phase 2) DD01 (phase 1) ZP2929 (phase I) TT-401 (phase II for D2T) Oxyntomodulin (OXM) analogs [276,343] G3215 (phase I) IBI362/LY3305677 (phase II) MOD-6031 (phase I) OPK-88003/LY294487 [273] (phase II) Oxytocin (phase II–for hypothalamic obesity) [289]	Nausea and vomiting (cotadutide and efinopegdutide) [333,344] Caution for patients with cardiovascular conditions (oxytocin) [345]
Pancreatic-entero-endocrine pathway GLP1/GIP/glucagon	Mimics the effects of GLP1, GIP, and glucagon activation Acts on the parietal cortex, insula, putamen, orbitofrontal cortex, arcuate nucleus and other hypothalamic regions, gastrointestinal tract, adipose tissue, liver	Agent type: energy intake GIP/GLP1/glucagon tri-agonists HM15211 (phase II for NASH) Peptide 20 (MAR423) (phase I) [331] Retatrutide (LY3437943) (phase II) [289,331,346] SAR441255 (phase I -stopped) [273] NN9423/NNC9204–1706 (phase I) [273]	Not known to date
Entero-endocrino pathway Cholescystokinin (CCK)	Short-term regulator of food intake reduction Transmits the satiety signal via the vagus to the brainstem, from which the satiety signal is projected to the hypothalamus [276]	Agent type: energy intake Cholecystokinin-1 receptor agonist GI181771X [347,348] (phase II–stopped) NN9056 (preclinical) [349,350] Positive allosteric modulators of CCK type 1 receptor–under investigation (preclinical) [351]	Delayed gastric emptying of solids [350], did not reduce body weight (GI181771X) [352] Induce acute pancreatitis and pancreas neoplasia in rodents, but not in primates [349]
Entero-endocrine pathway Peptide tyrosine tyrosine (PYY)	Co-secreted from the intestinal L cells as PYY1-36, together with GLP1 The bioactive form is PYY3-36 produced by cleavage of 2 amino-acid residues from PYY1-36 by DPP-IV PYY3–36 is a high-affinity agonist at the NPY receptor type 2 (Y2R). Decreases activity of NPY neurons and activate POMC neurons [276,353]	Agent type: energy intake PYY_3–36_ analogues NN9747 (PYY 1562 analogue) PYY analogue in combination with metreleptinb (leptin analogue) or amylin analog [350] NN9748, NNC0165-1875 (phase I) NNC0165-1875 in combination with semaglutide (phase II) [276] Combination of PYY, GLP-1, and oxyntomodulin administered as subcutaneous infusion [354]	Nausea was described in compounds from the same class [355]
Entero-endocrine pathway Neuropeptide Y	Orexigenic neuro peptide belonging to the neuropeptide Y family Is found at all levels of the gut–brain, and brain–gut axis [353] Promotes energy storage in white adipose tissue Inhibits brown adipose tissue activation [356]	Agent type: energy intake Type 5 neuropeptide Y receptor antagonist MK-0557 [278] (phase II) [275,357] Velneperit (phase IIb) [275,294,358]	Good tolerance [275]
Melanocortin system	Activation of POMC/CART neurons leads to the secretion of α- MSH, which activates MC4R to inhibit food intake Activation of NPY/AgRP neurons leads to the secretion of AgRP, which stimulates food intake through blocking of the melanocortin 4 receptor (MC4R) [276]	Agent type: energy intake Structurally related MC4R agonist Setmelanotide * approved in 2020 (FDA), 2021 (EMA)–treatment of obesity in patients aged 6 or older with proopiomelanocortin (POMC), proprotein convertase subtilisin/kexin type 1 (PCSK1) or leptin receptor (LEPR) deficiency, confirmed by genetic testing); approved in 2022 (FDA, EMA) for Bardet-Biedl syndrome) [276,287,289,359,360] LY2112688, MC4- NN-0453, MK-0493, AZD2820 (stopped in clinical phases due to lack of efficacy) [361]	Injection site reactions, hyperpigmentation, nausea, spontaneous penile erections in males, depression, and suicidal ideation (setmelanotide) [362]
Entero-endocrine pathway Ghrelin pathway	Short-term regulators, secreted in anticipation of food intake) Ghrelin is one of the most important orexigenic neuropeptides and represents the ligand of the growth hormone secretagogue receptor (GHS-R1a) In fat tissue, ghrelin increases fat storage [363] Uses vagal signaling, in order to stimulate food intake [363] Activation of NPY/AgRP neurons in the hypothalamus [276]	Agent type: energy intake Ghrelin neutralization: CYT009- GhrQb vaccine (phase I, lack of efficacy) NOX-B11-2 and NOX-B11-3 spiegelmers, antisense polyethylene glycol-modified L-oligonucleotides capable of specifically binding a target molecule (preclinical) [276,364] GHS-R1a Antagonists [D-Lys-3] GHRP-6, YIL-781, JMV2866, JMV2844, TZP-301 [364] GHS-R1a Inverse Agonists [D-Arg^1^, D-Phe^5^, D-Trp7,9, Leu11] substance P Bitter taste receptor (T2R) antagonists ARD-101 (phase II) [365] Ghrelin-O-acyltransferase (GOAT) inhibitor: GLWL 01 (phase II for Prader-Willi) [19,366] GHSR antagonists and inverse agonists: Liver-enriched antimicrobial peptide 2 (LEAP2), the des-acyl form of ghrelin (DAG) (phase I) [276,367]	AZP-531–discontinued due to hyperphagia in patients with Prader–Willi syndrome [276]
Leptin pathway	Long-term regulators of food intake Communicates signals to the cerebral cortex conforming to the amount of lipid stored in the organism. Inhibits orexigenic pathways and activate anorexigenic pathways targeted to suppress appetite Activation of POMC neurons and inhibition of AgRP neurons in the ARC [276,368] Site of action: adipose tissue, liver, hypothalamus [289]	Agent type: energy intake Recombinant analog of the human hormone leptin Metreleptin * approved in 2014 (FDA), 2018 (EMA) for individuals with congenital leptin deficiency MetHuLeptin [368] Pegylated recombinant leptin PEG-OB [368,369] Leptin sensitizers: ERX1000 (phase I) [273,289] Withaferin A (bioactive A compound derived from traditional Chinese medicinal herbs of the *Celastraceae* family) (phase I) Celastrol (C28 steroidal lactone derived from Ashwagandha) (preclinical) [370,371] Leptin/amylin discontinued [276]	Low effect in polygenetic obesity Antibodies appearance (metreleptin) [372]
Amylin pathway	Decreases homeostatic food intake Co-secreted with insulin from the pancreatic β- cells Activates calcitonin gene-related CGRP signaling through the AP area postrema. Signaling through the mesolimbic dopamine system in the ventral tegmental area and the nucleus accumbens (NAcc) [276,294]	Agent type: energy intake Amylin agonists Pramlintide registered for T2D treatment [373] Cagrilintide (phase III) in combination with semaglutide [276,289,294] AC164204, AC164209 (davalintide in combination with GLP-1R analogue) (preclinical) [275] NNC0174-0833 (phase II) [275,289] ZP8396 (phase I) [273,276]	Administered in different combinations [294]
Amylin and calcitonin pathway	Calcitonin of mammalian origin promotes insulin sensitivity Salmon calcitonin decreases gastric emptying, enhances energy expenditure, and promotes satiety [374]	Agent type: energy intake Dual- acting amylin and calcitonin receptor agonists (DACRAs) KBP-089 (phase II–T2D) Davalintide (AC2307) –discontinued KBP-088 [375] KBP-042 (phase II–T2D) [276]	Weight loss in animal models
Sodium-glucose co-transporters (SGLT) pathway	Blocks reabsorption of filtered glucose in the kidney, increasing urinary glucose excretion and reducing blood glucose levels Site of action: kidney, adipose tissue	Agent type: energy expenditure SGLT2 inhibitors registered for the treatment of T2D but exhibiting weight loss effects: canagliflozin, dapagliflozin, empagliflozin, bexagliflozin [19,294] Dual sodium-glucose transporter (SGLT)-1 and -2 inhibitor licogliflozin (LIK066) [376,377], sotagliflozin for T2D [378]	Gastrointestinal dose-related side effects: diarrhea, flatulence, urinary infections, ketoacidosis (SGLT2) SGLT2 and phentermine co administration resulted in significant body weight reduction from baseline compared to monotherapy [275]
Modulation of PPAR gamma pathway	Regulation of adipocyte differentiation and lipid storage	Agent type: energy storage AMG 133 (phase I) [379] MBL949 [289,380]	Not known to date
Phosphodiesterase-4 (PDE4) pathway	Influences the expression of the adipogenesis genes such as SREBP1C, FABP4, Glut4, and regulators as PPAR-γ via activation of the AMPK-mediated pathway [381]	Agent type: energy storage and expenditure PDE4/5 inhibitor Roflumilast (phase III) [382] Tadalafil (phase II)	Mainly gastro-intestinal [383]
Sirtuin 1 (Sirt1) pathway	Modulates energy metabolism Influences transcription of factors via the PPAR pathway [384]	Agent type: energy storage NS-0200 (combination of leucine, metformin, and sildenafil) (phase II) [385]	Not serious
Targeting mitochondrial uncouplers pathway	Increase energy expenditure, increase mitochondrial inefficiency, and renders ATP less efficient At therapeutic doses can protect cells against death but in high concentrations are cytotoxic due to a drop in ATP concentration and lysosomal membrane permeabilization [276]	Agent type: energy expenditure 2,4–dinitrophenol (DNP) 1933–1938 (FDA) BAM15 (preclinical) [276]	Side effects: hyperthermia, tachycardia, diaphoresis, fever, tachypnoea, and death [273] Benefits: improves insulin sensitivity in multiple tissues
Fibroblast growth factor 21 (FGF21) pathway	Secreted mainly from the liver in fasting conditions Activation of brown fat thermogenesis and augmented secretion of adiponectin [276,294,386]	Agent type: energy expenditure FGF21 analog: LY2405319 (modified human FGF21 expressed in yeast) PF05231023 (two FGF21 joint with an IgG backbone) [387] Pegbelfermin (BMS986036) (peglylated human FGF21) Efruxifermin (AKR-001 Fc-FGF21 engineered fusion protein) (phase I–for D2T) [388] AMG876 [246,299,386,387,389] FGF21-receptor agonists: C3201–HAS MimAb1 39F7 mAb FGF21 mimetics: BFKB8488A NGM313 [389,390] FGF21/FGFR1c/β-Klotho signaling LLF580 (phase I) MK-3655/NGM313 (phase I) NN9499/NNC0194-0499 (phase I)	Raised heart rate and blood pressure (PF-05231023) Moderate bone resorption
Farnesoid X receptor (FXR) (also known as bile acid receptor) pathway	Effects also mediated by FGF 19 and 21 CYP7A1 inhibition stimulates cholesterol excretion into bile and intestinal lumen [391]	Agent type: energy expenditure FXR agonist obeticholic acid derivatives: EDP-305, INT-767, INT-787 Non-steroidal compounds: MET409, tropifexor, cilofexorlization, vonafexor, TERN-101, ASC42, EDP-297, HPG1860, and HPG7233 (phases I and II for NASH) [391]	Pruritogenic potency of obeticholic acid derivatives [392]
Fibroblast growth factor receptor 4 (FGFR4) pathway	Decreases the body’s ability to store fat while simultaneously increasing fat burning and energy expenditure [393,394]	Agent type: energy expenditure FGFR4 inhibitor IONIS-FGFR4Rx an anti sense drug that diminishes the production of FGFR4 in the liver and fat tissues (phase II) [395]	Expected not to produce any CNS side effects are due to the fact that it is not distributed to the brain [396].
Macrophage inhibitory cytokine 1 (MIC-1; also known as growth differentiation factor GDF15) pathway	Belongs to TGF-β superfamily Activation of the GDNF family receptor α- like (GFRAL) [397] GDF15-GFRAL-mediated regulation of food intake is by a central mechanism [397] Possible induction of nausea and engagement of emetic neurocircuitries [276]	GDF15 agonist/analog LA- GFD15 (phase I) LY–3,463,251 (phase I) JNJ-9090/CIN-109 (phase I) [276]	Mild gastrointestinal side effects [398]
Cholinergic pathway	Releases GLP-1 and PYY [399]	Agent type: energy intake α7-nAChR agonist GTS-21/DMXB-A (phase I) [273]	Not known to date
Activin type II receptor (ActRII) pathway	Prevents the actions of natural ligands that negatively regulate skeletal muscle growth Activates functional brown adipogenesis and thermogenesis through increasing mitochondrial function [275]	Agent type: energy expenditure A fully humanized monoclonal antibody against activin type 2 receptors Bimagrumab (BYM338) (phase II) [400]	Mild diarrhea and muscle spasms [400]
Glabridin (prenylated isoflavan from the roots of *Glycyrrhiza glabra*) [401]	Acts on signaling pathways, including NF-κB, MAPK, Wnt/β-catenin, ERα/SRC-1, PI3K/AKT, and AMPK Site of action: muscles, Liver	Agent type: energy expenditure Glabridin analogue HSG4112 (phase I) [273,402]	Not known to date
*Labisia pumila* extract	Upregulation of PPARgamma pathway [403]	Agent type: energy storage SKF7 (phase II) was accepted as a food supplement in 2015 in the EU [404,405]	Not known to date
Probiotics	Anaerobic, Gram The negative and mucin-degrading bacterium of the phylum *Verrucomicrobia* [406]	Agent type: energy absorption *Akkermansia muciniphila* WST01 strain (phase II) [407]	Damaged the intestinal barrier Changes the bile acid metabolic profile when administered after antibiotics [408]
Melatonin pathway	Influences circadian rhythm, gut microbiota, sleep patterns, α7nAChR, and the opioidergic system [409,410] Protects against obesity-induced renal side-effects by inhibiting endoplasmic reticulum stress/apoptosis pathway [411]	Agent type: energy expenditure [412] Melatonin receptor agonists for the treatment of circadian rhythm sleep-wake disorders Prolonged-release melatonin (approved by EMA) Agomelatine (approved by EMA) Tasimelteon (approved by FDA and EMA) Ramelteon (approved by FDA) [410,413]	Safe in the short-term treatment and without abuse potential (tasimelteon) [414] Low-dose melatonin supplementation was not associated with low testosterone levels [415]
Thyroid hormones pathway	Upregulates free fatty acid uptake and oxidation stimulating lipolysis Enhances mitochondrial biogenesis and respiration, leading to increased energy expenditure. Activation of bile acid synthesis [391]	Agent type: energy expenditure THR-β agonist ASC41 (phase II for NASH) [416]	Reduced cardiac effects due to the fact that the TRβ receptor subtype is mainly expressed in the liver compared with TRα, which is mainly expressed in the heart [417]
Methionine aminopeptidase (MetAP) 2 pathway [299]	Reduces fat biosynthesis, and increases fat oxidation and lipolysis [288]	Agent type: energy expenditure MetAP2 inhibitor Beloranib (ZGN-440) (phase III for Prader-Willi Syndrome) [418]	Injection site bruising, venous thrombotic events [419]
Vitamin E pathway	Regulates pathways of lipid metabolism and fatty acid biosynthesis Reduces the expression of transcription factors regulating adipogenesis and increasing apoptosis of adipocytes [420]	Tocotrienols (phase I) [289,421]	Not known to date
Diacylglycerol acyltransferase 1 (DGAT) pathway	DGAT1 plays a role in very VLDL synthesis DGAT2 plays a role in steatosis	Agent type: energy storage DGAT1 inhibitor AZD7687 [422] DGAT2 inhibitor Ervogastat/PF-06865571 [273]	Nausea, vomiting and diarrhoea (DGAT1 inhibitor) [423] Well tolerated (DGAT2 inhibitor) [423]
Monoacylglycerol O-acyltransferase 2 (MGAT2) inhibitor	Facilitates the absorption of dietary fat in the small intestine Interferes with triglyceride resynthesis in the small intestine Plays a role in hepatic lipid metabolism [424,425]	Agent type: energy storage MGAT2 inhibitor BMS-963272 (phase I) S-309309 [273] JTP-103237 (preclinical) [424,426,427]	Not known to date
Fat absorption	Reduces fat absorb-tion by up to 30% [287,428]	Agent type: energy absorption Intestinal lipase inhibitor Orlistat * approved in 1999 (FDA), 1998 (EMA) for long time use Inhibits pancreatic and gastric lipase Cetilistat (phase II)	Oily rectal leakage, abdomi-nal distress, abdominal pain, flatulence with discharge, fecal urgency, steatorrhea, fecal incontinence, and increased defecation [286,289] Better tolerance of cetilistat compared to orlistat [294]
Nutrients absorption	Mechanical mode of action Composed of modi-field cellulose cross-linked with citric acid that absorbs water to occupy about one-fourth of the average stomach volume, promoting fullness [287]	Agent type: energy absorption Hydrogel matrix Gelesis100 (FDA approved in 2019 for adults with a BMI of at least 25 kg/m^2^, with or without comorbidities) [429,430]	Side effects: abdominal dis-tension, infrequent bowel movements, and dyspepsia [287] Should be considered food when administered simultaneously with drugs [287,431]

* Drugs in current use.

## Data Availability

Not applicable.

## References

[1] Products—Health E Stats—Homepage. http://www.cdc.gov/nchs/products/pubs/pubd/hestats/.

[2] Smith K.B., Smith M.S. (2016). Obesity Statistics. Prim. Care.

[3] Conway B., Rene A. (2004). Obesity as a Disease: No Lightweight Matter. Obes. Rev..

[4] Singer-Englar T., Barlow G., Mathur R. (2019). Obesity, Diabetes, and the Gut Microbiome: An Updated Review. Expert Rev. Gastroenterol. Hepatol..

[5] Weiss G.A., Hennet T. (2017). Mechanisms and Consequences of Intestinal Dysbiosis. Cell. Mol. Life Sci..

[6] Fontaine K.R., Redden D.T., Wang C., Westfall A.O., Allison D.B. (2003). Years of Life Lost Due to Obesity. JAMA.

[7] Sacks F.M., Bray G.A., Carey V.J., Smith S.R., Ryan D.H., Anton S.D., McManus K., Champagne C.M., Bishop L.M., Laranjo N. (2009). Comparison of Weight-Loss Diets with Different Compositions of Fat, Protein, and Carbohydrates. N. Engl. J. Med..

[8] Lin X., Li H. (2021). Obesity: Epidemiology, Pathophysiology, and Therapeutics. Front. Endocrinol..

[9] Cuevas-Sierra A., Ramos-Lopez O., Riezu-Boj J.I., Milagro F.I., Martinez J.A. (2019). Diet, Gut Microbiota, and Obesity: Links with Host Genetics and Epigenetics and Potential Applications. Adv. Nutr..

[10] Zhou M., Shao J., Wu C.-Y., Shu L., Dong W., Liu Y., Chen M., Wynn R.M., Wang J., Wang J. (2019). Targeting BCAA Catabolism to Treat Obesity-Associated Insulin Resistance. Diabetes.

[11] Asadi A., Shadab Mehr N., Mohamadi M.H., Shokri F., Heidary M., Sadeghifard N., Khoshnood S. (2022). Obesity and Gut-Microbiota-Brain Axis: A Narrative Review. J. Clin. Lab. Anal..

[12] White P.J., McGarrah R.W., Herman M.A., Bain J.R., Shah S.H., Newgard C.B. (2021). Insulin Action, Type 2 Diabetes, and Branched-Chain Amino Acids: A Two-Way Street. Mol. Metab..

[13] Zhang Y., Fischer K.E., Soto V., Liu Y., Sosnowska D., Richardson A., Salmon A.B. (2015). Obesity-Induced Oxidative Stress, Accelerated Functional Decline with Age and Increased Mortality in Mice. Arch. Biochem. Biophys..

[14] Luft V.C., Schmidt M.I., Pankow J.S., Couper D., Ballantyne C.M., Young J.H., Duncan B.B. (2013). Chronic Inflammation Role in the Obesity-Diabetes Association: A Case-Cohort Study. Diabetol. Metab. Syndr..

[15] Avila C., Holloway A.C., Hahn M.K., Morrison K.M., Restivo M., Anglin R., Taylor V.H. (2015). An Overview of Links between Obesity and Mental Health. Curr. Obes. Rep..

[16] Farr O.M., Sloan D.M., Keane T.M., Mantzoros C.S. (2014). Stress- and PTSD-Associated Obesity and Metabolic Dysfunction: A Growing Problem Requiring Further Research and Novel Treatments. Metabolism.

[17] Penninx B.W.J.H., Lange S.M.M. (2018). Metabolic Syndrome in Psychiatric Patients: Overview, Mechanisms, and Implications. Dialogues Clin. Neurosci..

[18] Schachter J., Martel J., Lin C.-S., Chang C.-J., Wu T.-R., Lu C.-C., Ko Y.-F., Lai H.-C., Ojcius D.M., Young J.D. (2018). Effects of Obesity on Depression: A Role for Inflammation and the Gut Microbiota. Brain Behav. Immun..

[19] Tak Y.J., Lee S.Y. (2021). Long-Term Efficacy and Safety of Anti-Obesity Treatment: Where Do We Stand?. Curr. Obes. Rep..

[20] Arterburn D.E., Telem D.A., Kushner R.F., Courcoulas A.P. (2020). Benefits and Risks of Bariatric Surgery in Adults: A Review. JAMA.

[21] Geiger B.M., Pothos E.N. (2019). Translating Animal Models of Obesity and Diabetes to the Clinic. Handbook of Behavioral Neuroscience.

[22] Martins T., Castro-Ribeiro C., Lemos S., Ferreira T., Nascimento-Gonçalves E., Rosa E., Oliveira P.A., Antunes L.M. (2022). Murine Models of Obesity. Obesities.

[23] Suleiman J.B., Mohamed M., Bakar A.B.A. (2020). A Systematic Review on Different Models of Inducing Obesity in Animals: Advantages and Limitations. J. Adv. Vet. Anim. Res..

[24] Kleinert M., Clemmensen C., Hofmann S.M., Moore M.C., Renner S., Woods S.C., Huypens P., Beckers J., de Angelis M.H., Schürmann A. (2018). Animal Models of Obesity and Diabetes Mellitus. Nat. Rev. Endocrinol..

[25] Kanasaki K., Koya D. (2011). Biology of Obesity: Lessons from Animal Models of Obesity. J. Biomed. Biotechnol..

[26] Lutz T.A., Woods S.C. (2012). Overview of Animal Models of Obesity. Curr. Protoc. Pharmacol..

[27] Speakman J., Hambly C., Mitchell S., Król E. (2008). The Contribution of Animal Models to the Study of Obesity. Lab. Anim..

[28] Ferguson D., Blenden M., Hutson I., Du Y., Harris C.A. (2018). Mouse Embryonic Fibroblasts Protect Ob/Ob Mice from Obesity and Metabolic Complications. Endocrinology.

[29] Kitada M., Ogura Y., Koya D. (2016). Rodent Models of Diabetic Nephropathy: Their Utility and Limitations. Int. J. Nephrol. Renovasc. Dis..

[30] Bates S.H., Stearns W.H., Dundon T.A., Schubert M., Tso A.W.K., Wang Y., Banks A.S., Lavery H.J., Haq A.K., Maratos-Flier E. (2003). STAT3 Signalling Is Required for Leptin Regulation of Energy Balance but Not Reproduction. Nature.

[31] Coleman D.L., Eicher E.M. (1990). Fat (Fat) and Tubby (Tub): Two Autosomal Recessive Mutations Causing Obesity Syndromes in the Mouse. J. Hered..

[32] Kleyn P.W., Fan W., Kovats S.G., Lee J.J., Pulido J.C., Wu Y., Berkemeier L.R., Misumi D.J., Holmgren L., Charlat O. (1996). Identification and Characterization of the Mouse Obesity Gene Tubby: A Member of a Novel Gene Family. Cell.

[33] Yorek M.A. (2016). Alternatives to the Streptozotocin-Diabetic Rodent. Int. Rev. Neurobiol..

[34] Zucker L.M., Zucker T.F. (1961). Fatty, a new mutation in the rat. J. Hered..

[35] Van der Spek R., Kreier F., Fliers E., Kalsbeek A. (2012). Circadian Rhythms in White Adipose Tissue. Prog. Brain Res..

[36] Bi S., Moran T.H. (2002). Actions of CCK in the Controls of Food Intake and Body Weight: Lessons from the CCK-A Receptor Deficient OLETF Rat. Neuropeptides.

[37] Fang J.-Y., Lin C.-H., Huang T.-H., Chuang S.-Y. (2019). In Vivo Rodent Models of Type 2 Diabetes and Their Usefulness for Evaluating Flavonoid Bioactivity. Nutrients.

[38] Wang Y.-W., Sun G.-D., Sun J., Liu S.-J., Wang J., Xu X.-H., Miao L.-N. (2013). Spontaneous Type 2 Diabetic Rodent Models. J. Diabetes Res..

[39] Rodrigues R. (2016). A Comprehensive Review: The Use of Animal Models in Diabetes Research. J. Anal. Pharm. Res..

[40] Scroyen I., Hemmeryckx B., Lijnen H.R. (2013). From Mice to Men--Mouse Models in Obesity Research: What Can We Learn?. Thromb. Haemost..

[41] Fajardo R.J., Karim L., Calley V.I., Bouxsein M.L. (2014). A Review of Rodent Models of Type 2 Diabetic Skeletal Fragility. J. Bone Miner. Res..

[42] Allan M.F., Eisen E.J., Pomp D. (2004). The M16 Mouse: An Outbred Animal Model of Early Onset Polygenic Obesity and Diabesity. Obes. Res..

[43] Huijbers I.J. (2017). Generating Genetically Modified Mice: A Decision Guide. Site-Specific Recombinases: Methods in Molecular Biology.

[44] Hui D.Y. (1998). Utility and Importance of Gene Knockout Animals for Nutritional and Metabolic Research. J. Nutr..

[45] Tschöp M., Heiman M.L. (2002). Overview of Rodent Models for Obesity Research. Curr. Protoc. Neurosci..

[46] Speakman J., Hambly C., Mitchell S., Król E. (2007). Animal Models of Obesity. Obes. Rev..

[47] De Moura E., Dias M., Dos Reis S.A., da Conceição L.L., de Oliveira Sediyama C.M.N., Pereira S.S., de Oliveira L.L., do Carmo Gouveia Peluzio M., Martinez J.A., Milagro F.I. (2021). Diet-Induced Obesity in Animal Models: Points to Consider and Influence on Metabolic Markers. Diabetol. Metab. Syndr..

[48] Thaker V.V. (2017). Genetic and Epigenetic Causes of Obesity. Adolesc. Med. State Art Rev..

[49] Huvenne H., Dubern B., Clément K., Poitou C. (2016). Rare Genetic Forms of Obesity: Clinical Approach and Current Treatments in 2016. Obes. Facts.

[50] Stunkard A.J., Sørensen T.I., Hanis C., Teasdale T.W., Chakraborty R., Schull W.J., Schulsinger F. (1986). An Adoption Study of Human Obesity. N. Engl. J. Med..

[51] Bouchard C., Tremblay A., Després J.P., Nadeau A., Lupien P.J., Thériault G., Dussault J., Moorjani S., Pinault S., Fournier G. (1990). The Response to Long-Term Overfeeding in Identical Twins. N. Engl. J. Med..

[52] Wardle J., Carnell S., Haworth C.M., Plomin R. (2008). Evidence for a Strong Genetic Influence on Childhood Adiposity despite the Force of the Obesogenic Environment. Am. J. Clin. Nutr..

[53] Yadav H.M., Jawahar A. (2023). Environmental Factors and Obesity.

[54] Loos R.J.F., Yeo G.S.H. (2022). The Genetics of Obesity: From Discovery to Biology. Nat. Rev. Genet..

[55] Irizarry K.A., Haqq A.M. (2018). Syndromic Obesity. Contemporary Endocrinology.

[56] Fermin Gutierrez M.A., Mendez M.D. (2023). Prader-Willi Syndrome.

[57] Forsyth R., Gunay-Aygun M., Adam M.P., Mirzaa G.M., Pagon R.A., Wallace S.E., Bean L.J.H., Gripp K.W., Amemiya A. (2003). Bardet-Biedl Syndrome Overview. GeneReviews^®^.

[58] Funcke J.-B., von Schnurbein J., Lennerz B., Lahr G., Debatin K.-M., Fischer-Posovszky P., Wabitsch M. (2014). Monogenic Forms of Childhood Obesity Due to Mutations in the Leptin Gene. Mol. Cell. Pediatr..

[59] Çetinkaya S., Güran T., Kurnaz E., Keskin M., Sağsak E., Savaş Erdeve S., Suntharalingham J.P., Buonocore F., Achermann J.C., Aycan Z. (2018). A Patient with Proopiomelanocortin Deficiency: An Increasingly Important Diagnosis to Make. J. Clin. Res. Pediatr. Endocrinol..

[60] Abdullah S., Reginold W., Kiss C., Harrison K.J., MacKenzie J.J. (2016). Melanocortin-4 Receptor Deficiency Phenotype with an Interstitial 18q Deletion: A Case Report of Severe Childhood Obesity and Tall Stature. Case Rep. Pediatr..

[61] Xu B., Xie X. (2016). Neurotrophic Factor Control of Satiety and Body Weight. Nat. Rev. Neurosci..

[62] Doche M.E., Bochukova E.G., Su H.-W., Pearce L.R., Keogh J.M., Henning E., Cline J.M., Saeed S., Dale A., Cheetham T. (2012). Human SH2B1 Mutations Are Associated with Maladaptive Behaviors and Obesity. J. Clin. Investig..

[63] Sakellariou G.K., Jackson M.J., Vasilaki A. (2014). Redefining the Major Contributors to Superoxide Production in Contracting Skeletal Muscle. The Role of NAD(P)H Oxidases. Free Radic. Res..

[64] Matsumoto S., Koshiishi I., Inoguchi T., Nawata H., Utsumi H. (2003). Confirmation of Superoxide Generation via Xanthine Oxidase in Streptozotocin-Induced Diabetic Mice. Free Radic. Res..

[65] Babior B.M., Lambeth J.D., Nauseef W. (2002). The Neutrophil NADPH Oxidase. Arch. Biochem. Biophys..

[66] St-Pierre J., Buckingham J.A., Roebuck S.J., Brand M.D. (2002). Topology of Superoxide Production from Different Sites in the Mitochondrial Electron Transport Chain. J. Biol. Chem..

[67] Gough D.R., Cotter T.G. (2011). Hydrogen Peroxide: A Jekyll and Hyde Signalling Molecule. Cell Death Dis..

[68] Da Costa L.A., Badawi A., El-Sohemy A. (2012). Nutrigenetics and Modulation of Oxidative Stress. Ann. Nutr. Metab..

[69] Johnson L.J., Meacham S.L., Kruskall L.J. (2003). The Antioxidants--Vitamin C, Vitamin E, Selenium, and Carotenoids. J. Agromed..

[70] Vincent H.K., Innes K.E., Vincent K.R. (2007). Oxidative Stress and Potential Interventions to Reduce Oxidative Stress in Overweight and Obesity. Diabetes Obes. Metab..

[71] Keaney J.F., Larson M.G., Vasan R.S., Wilson P.W.F., Lipinska I., Corey D., Massaro J.M., Sutherland P., Vita J.A., Benjamin E.J. (2003). Obesity and Systemic Oxidative Stress: Clinical Correlates of Oxidative Stress in the Framingham Study. Arterioscler. Thromb. Vasc. Biol..

[72] Ozata M., Mergen M., Oktenli C., Aydin A., Sanisoglu S.Y., Bolu E., Yilmaz M.I., Sayal A., Isimer A., Ozdemir I.C. (2002). Increased Oxidative Stress and Hypozincemia in Male Obesity. Clin. Biochem..

[73] Vincent H.K., Taylor A.G. (2006). Biomarkers and Potential Mechanisms of Obesity-Induced Oxidant Stress in Humans. Int. J. Obes..

[74] Tóbon-Velasco J.C., Cuevas E., Torres-Ramos M.A. (2014). Receptor for AGEs (RAGE) as Mediator of NF-KB Pathway Activation in Neuroinflammation and Oxidative Stress. CNS Neurol. Disord. Drug Targets.

[75] Evans J.L., Goldfine I.D., Maddux B.A., Grodsky G.M. (2002). Oxidative Stress and Stress-Activated Signaling Pathways: A Unifying Hypothesis of Type 2 Diabetes. Endocr. Rev..

[76] Matsuda M., Shimomura I. (2013). Increased Oxidative Stress in Obesity: Implications for Metabolic Syndrome, Diabetes, Hypertension, Dyslipidemia, Atherosclerosis, and Cancer. Obes. Res. Clin. Pract..

[77] Obrosova I.G., Minchenko A.G., Vasupuram R., White L., Abatan O.I., Kumagai A.K., Frank R.N., Stevens M.J. (2003). Aldose Reductase Inhibitor Fidarestat Prevents Retinal Oxidative Stress and Vascular Endothelial Growth Factor Overexpression in Streptozotocin-Diabetic Rats. Diabetes.

[78] Karczewski J., Śledzińska E., Baturo A., Jończyk I., Maleszko A., Samborski P., Begier-Krasińska B., Dobrowolska A. (2018). Obesity and Inflammation. Eur. Cytokine Netw..

[79] Fonseca-Alaniz M.H., Takada J., Alonso-Vale M.I.C., Lima F.B. (2007). Adipose Tissue as an Endocrine Organ: From Theory to Practice. J. Pediatr..

[80] Fischer-Posovszky P., Möller P. (2020). Das Fettgewebe Im Fokus Des Immunsystems: Adipositasassoziierte Inflammation. Pathologe.

[81] Liu T., Zhang L., Joo D., Sun S.-C. (2017). NF-ΚB Signaling in Inflammation. Signal Transduct. Target. Ther..

[82] Guilherme A., Virbasius J.V., Puri V., Czech M.P. (2008). Adipocyte Dysfunctions Linking Obesity to Insulin Resistance and Type 2 Diabetes. Nat. Rev. Mol. Cell Biol..

[83] Marseglia L., Manti S., D’Angelo G., Nicotera A., Parisi E., Di Rosa G., Gitto E., Arrigo T. (2014). Oxidative Stress in Obesity: A Critical Component in Human Diseases. Int. J. Mol. Sci..

[84] Kim S.-R., Bae Y.-H., Bae S.-K., Choi K.-S., Yoon K.-H., Koo T.H., Jang H.-O., Yun I., Kim K.-W., Kwon Y.-G. (2008). Visfatin Enhances ICAM-1 and VCAM-1 Expression through ROS-Dependent NF-KappaB Activation in Endothelial Cells. Biochim. Biophys. Acta.

[85] Fernández-Sánchez A., Madrigal-Santillán E., Bautista M., Esquivel-Soto J., Morales-González A., Esquivel-Chirino C., Durante-Montiel I., Sánchez-Rivera G., Valadez-Vega C., Morales-González J.A. (2011). Inflammation, Oxidative Stress, and Obesity. Int. J. Mol. Sci..

[86] Tereshin E.V. (2007). A role of fatty acids in the development of oxidative stress in aging. A hypothesis. Adv. Gerontol..

[87] Goossens G.H. (2008). The Role of Adipose Tissue Dysfunction in the Pathogenesis of Obesity-Related Insulin Resistance. Physiol. Behav..

[88] Littleton S.W. (2012). Impact of Obesity on Respiratory Function. Respirology.

[89] Gami A.S., Caples S.M., Somers V.K. (2003). Obesity and Obstructive Sleep Apnea. Endocrinol. Metab. Clin. N. Am..

[90] Kargar B., Zamanian Z., Hosseinabadi M.B., Gharibi V., Moradi M.S., Cousins R. (2021). Understanding the Role of Oxidative Stress in the Incidence of Metabolic Syndrome and Obstructive Sleep Apnea. BMC Endocr. Disord..

[91] Staerck C., Gastebois A., Vandeputte P., Calenda A., Larcher G., Gillmann L., Papon N., Bouchara J.-P., Fleury M.J.J. (2017). Microbial Antioxidant Defense Enzymes. Microb. Pathog..

[92] Lindqvist D., Dhabhar F.S., James S.J., Hough C.M., Jain F.A., Bersani F.S., Reus V.I., Verhoeven J.E., Epel E.S., Mahan L. (2017). Oxidative Stress, Inflammation and Treatment Response in Major Depression. Psychoneuroendocrinology.

[93] Kumari S., Verma A.K., Rungta S., Mitra R., Srivastava R., Kumar N. (2013). Serum Prolidase Activity, Oxidant and Antioxidant Status in Nonulcer Dyspepsia and Healthy Volunteers. ISRN Biochem..

[94] Verma A.K., Chandra S., Singh R.G., Singh T.B., Srivastava S., Srivastava R. (2014). Serum Prolidase Activity and Oxidative Stress in Diabetic Nephropathy and End Stage Renal Disease: A Correlative Study with Glucose and Creatinine. Biochem. Res. Int..

[95] Panth N., Paudel K.R., Parajuli K. (2016). Reactive Oxygen Species: A Key Hallmark of Cardiovascular Disease. Adv. Med..

[96] Cheung E.C., Vousden K.H. (2022). The Role of ROS in Tumour Development and Progression. Nat. Rev. Cancer.

[97] Popli S., Badgujar P.C., Agarwal T., Bhushan B., Mishra V. (2022). Persistent Organic Pollutants in Foods, Their Interplay with Gut Microbiota and Resultant Toxicity. Sci. Total Environ..

[98] Tudi M., Daniel Ruan H., Wang L., Lyu J., Sadler R., Connell D., Chu C., Phung D.T. (2021). Agriculture Development, Pesticide Application and Its Impact on the Environment. Int. J. Environ. Res. Public Health.

[99] Guo W., Pan B., Sakkiah S., Yavas G., Ge W., Zou W., Tong W., Hong H. (2019). Persistent Organic Pollutants in Food: Contamination Sources, Health Effects and Detection Methods. Int. J. Environ. Res. Public Health.

[100] Kelishadi R., Poursafa P., Jamshidi F. (2013). Role of Environmental Chemicals in Obesity: A Systematic Review on the Current Evidence. J. Environ. Public Health.

[101] Heindel J.J., Balbus J., Birnbaum L., Brune-Drisse M.N., Grandjean P., Gray K., Landrigan P.J., Sly P.D., Suk W., Cory Slechta D. (2015). Developmental Origins of Health and Disease: Integrating Environmental Influences. Endocrinology.

[102] Heindel J.J., Blumberg B., Cave M., Machtinger R., Mantovani A., Mendez M.A., Nadal A., Palanza P., Panzica G., Sargis R. (2017). Metabolism Disrupting Chemicals and Metabolic Disorders. Reprod. Toxicol..

[103] Spalding K.L., Arner E., Westermark P.O., Bernard S., Buchholz B.A., Bergmann O., Blomqvist L., Hoffstedt J., Näslund E., Britton T. (2008). Dynamics of Fat Cell Turnover in Humans. Obstet. Gynecol. Surv..

[104] Janesick A.S., Blumberg B. (2016). Obesogens: An Emerging Threat to Public Health. Am. J. Obstet. Gynecol..

[105] Jeffery E., Church C.D., Holtrup B., Colman L., Rodeheffer M.S. (2015). Rapid Depot-Specific Activation of Adipocyte Precursor Cells at the Onset of Obesity. Nat. Cell Biol..

[106] Francis C.E., Allee L., Nguyen H., Grindstaff R.D., Miller C.N., Rayalam S. (2021). Endocrine Disrupting Chemicals: Friend or Foe to Brown and Beige Adipose Tissue?. Toxicology.

[107] Foley B., Doheny D.L., Black M.B., Pendse S.N., Wetmore B.A., Clewell R.A., Andersen M.E., Deisenroth C. (2017). Editor’s Highlight: Screening ToxCast Prioritized Chemicals for PPARG Function in a Human Adipose-Derived Stem Cell Model of Adipogenesis. Toxicol. Sci..

[108] Ren X.-M., Kuo Y., Blumberg B. (2020). Agrochemicals and Obesity. Mol. Cell. Endocrinol..

[109] Lo S., King I., Alléra A., Klingmüller D. (2007). Effects of Various Pesticides on Human 5alpha-Reductase Activity in Prostate and LNCaP Cells. Toxicol. In Vitro.

[110] Abass K., Pelkonen O. (2013). The Inhibition of Major Human Hepatic Cytochrome P450 Enzymes by 18 Pesticides: Comparison of the N-in-One and Single Substrate Approaches. Toxicol. In Vitro.

[111] Blizard D., Sueyoshi T., Negishi M., Dehal S.S., Kupfer D. (2001). Mechanism of Induction of Cytochrome P450 Enzymes by the Proestrogenic Endocrine Disruptor Pesticide-Methoxychlor: Interactions of Methoxychlor Metabolites with the Constitutive Androstane Receptor System. Drug Metab. Dispos..

[112] Zoeller T.R. (2010). Environmental Chemicals Targeting Thyroid. Hormones.

[113] Mendoza A., Hollenberg A.N. (2017). New Insights into Thyroid Hormone Action. Pharmacol. Ther..

[114] Condette J., Khorsi-Cauet C., Morliere H., Zabijak P., Reygner L., Bach J., Gay-Queheillard V. (2014). Increased Gut Permeability and Bacterial Translocation after Chronic Chlorpyrifos Exposure in Rats. PLoS ONE.

[115] Yuan L., Lin J., Xu Y., Peng Y., Clark J.M., Gao R., Sun P.Y. (2019). Deltamethrin Promotes Adipogenesis via AMPKα and ER Stress-Mediated Pathway in 3T3-L1 Adipocytes and *Caenorhabditis elegans*. Food Chem. Toxicol..

[116] Mao Q., Manservisi F., Panzacchi S., Mandrioli D., Menghetti I., Vornoli A., Bua L., Falcioni L., Lesseur C., Chen J. (2018). The Ramazzini Institute 13-Week Pilot Study on Glyphosate and Roundup Administered at Human-Equivalent Dose to Sprague Dawley Rats: Effects on the Microbiome. Environ. Health.

[117] Lee D.-H., Steffes M.W., Sjödin A., Jones R.S., Needham L.L., Jacobs D.R. (2011). Low Dose Organochlorine Pesticides and Polychlorinated Biphenyls Predict Obesity, Dyslipidemia, and Insulin Resistance among People Free of Diabetes. PLoS ONE.

[118] Van der Kolk B. (2000). Posttraumatic Stress Disorder and the Nature of Trauma. Dialogues Clin. Neurosci..

[119] Wickramasinghek K., Williams J., Rakovac I., Grosso G., Heinen M. (2022). Key Messages of the WHO European Regional Obesity Report. Eur. J. Public Health.

[120] Sharon-David H., Tenenbaum G. (2017). The Effectiveness of Exercise Interventions on Coping with Stress: Research Synthesis. Stud. Sport Humanit..

[121] Sinha R. (2008). Chronic Stress, Drug Use, and Vulnerability to Addiction. Ann. N. Y. Acad. Sci..

[122] Ferrario C.R. (2017). Food Addiction and Obesity. Neuropsychopharmacology.

[123] Neumann N.J., Fasshauer M. (2022). Added Flavors: Potential Contributors to Body Weight Gain and Obesity?. BMC Med..

[124] Brondel L., Quilliot D., Mouillot T., Khan N.A., Bastable P., Boggio V., Leloup C., Pénicaud L. (2022). Taste of Fat and Obesity: Different Hypotheses and Our Point of View. Nutrients.

[125] Campana B., Brasiel P.G., de Aguiar A.S., Dutra S.C.P.L. (2019). Obesity and Food Addiction: Similarities to Drug Addiction. Obes. Med..

[126] Minks J. (2020). Examining the Relationship between Stress and Insulin Resistance in Civilians and Veterans. Clin. Diabetes Res..

[127] Tosato S., Bonetto C., Lopizzo N., Cattane N., Barcella M., Turco G., Ruggeri M., Provasi S., Tomassi S., Dazzan P. (2021). Childhood and Adulthood Severe Stressful Experiences and Biomarkers Related to Glucose Metabolism: A Possible Association?. Front. Psychiatry.

[128] Kim E.J., Pellman B., Kim J.J. (2015). Stress Effects on the Hippocampus: A Critical Review. Learn. Mem..

[129] Merabet N., Lucassen P.J., Crielaard L., Stronks K., Quax R., Sloot P.M.A., la Fleur S.E., Nicolaou M. (2022). How Exposure to Chronic Stress Contributes to the Development of Type 2 Diabetes: A Complexity Science Approach. Front. Neuroendocrinol..

[130] Ingrosso D.M.F., Primavera M., Samvelyan S., Tagi V.M., Chiarelli F. (2022). Stress and Diabetes Mellitus: Pathogenetic Mechanisms and Clinical Outcome. Horm. Res. Paediatr..

[131] Michopoulos V., Vester A., Neigh G. (2016). Posttraumatic Stress Disorder: A Metabolic Disorder in Disguise?. Exp. Neurol..

[132] Esler M., Straznicky N., Eikelis N., Masuo K., Lambert G., Lambert E. (2006). Mechanisms of Sympathetic Activation in Obesity-Related Hypertension. Hypertension.

[133] Thorp A.A., Schlaich M.P. (2015). Relevance of Sympathetic Nervous System Activation in Obesity and Metabolic Syndrome. J. Diabetes Res..

[134] Yaribeygi H., Maleki M., Butler A.E., Jamialahmadi T., Sahebkar A. (2022). Molecular Mechanisms Linking Stress and Insulin Resistance. EXCLI J..

[135] Jiang S., Postovit L., Cattaneo A., Binder E.B., Aitchison K.J. (2019). Epigenetic Modifications in Stress Response Genes Associated With Childhood Trauma. Front. Psychiatry.

[136] Mellon S.H., Gautam A., Hammamieh R., Jett M., Wolkowitz O.M. (2018). Metabolism, Metabolomics, and Inflammation in Posttraumatic Stress Disorder. Biol. Psychiatry.

[137] Scherrer J.F., Salas J., Norman S.B., Schnurr P.P., Chard K.M., Tuerk P., Schneider F.D., van den Berk-Clark C., Cohen B.E., Friedman M.J. (2019). Association Between Clinically Meaningful Posttraumatic Stress Disorder Improvement and Risk of Type 2 Diabetes. JAMA Psychiatry.

[138] Aaseth J., Roer G.E., Lien L., Bjørklund G. (2019). Is There a Relationship between PTSD and Complicated Obesity? A Review of the Literature. Biomed. Pharmacother..

[139] Blessing E.M., Reus V., Mellon S.H., Wolkowitz O.M., Flory J.D., Bierer L., Lindqvist D., Dhabhar F., Li M., Qian M. (2017). Biological Predictors of Insulin Resistance Associated with Posttraumatic Stress Disorder in Young Military Veterans. Psychoneuroendocrinology.

[140] Kohut A.O., Chaban O.S., Dolynskyi R.G., Sandal O.S., Bursa A.I., Bobryk M.I., Vertel A.V. (2022). The features of posttraumatic stress disorder development in patients with *Diabetes mellitus* 2 type. Wiad. Lek..

[141] Roberts A.L., Agnew-Blais J.C., Spiegelman D., Kubzansky L.D., Mason S.M., Galea S., Hu F.B., Rich-Edwards J.W., Koenen K.C. (2015). Posttraumatic Stress Disorder and Incidence of Type 2 Diabetes Mellitus in a Sample of Women: A 22-Year Longitudinal Study. JAMA Psychiatry.

[142] Ehlert U. (2013). Enduring Psychobiological Effects of Childhood Adversity. Psychoneuroendocrinology.

[143] Masodkar K., Johnson J., Peterson M.J. (2016). A Review of Posttraumatic Stress Disorder and Obesity: Exploring the Link. Prim. Care Companion CNS Disord..

[144] Wischik D.L., Magny-Normilus C., Whittemore R. (2019). Risk Factors of Obesity in Veterans of Recent Conflicts: Need for Diabetes Prevention. Curr. Diab. Rep..

[145] Stefanovics E.A., Potenza M.N., Pietrzak R.H. (2020). PTSD and Obesity in U.S. Military Veterans: Prevalence, Health Burden, and Suicidality. Psychiatry Res..

[146] Nagai M., Ohira T., Maeda M., Yasumura S., Miura I., Itagaki S., Harigane M., Takase K., Yabe H., Sakai A. (2021). The Association between Body Mass Index and Recovery from Post-Traumatic Stress Disorder after the Nuclear Accident in Fukushima. Sci. Rep..

[147] Scherrer J.F., Salas J., Lustman P.J., van den Berk-Clark C., Schnurr P.P., Tuerk P., Cohen B.E., Friedman M.J., Norman S.B., Schneider F.D. (2018). The Role of Obesity in the Association between Posttraumatic Stress Disorder and Incident Diabetes. JAMA Psychiatry.

[148] Hoerster K.D., Campbell S., Dolan M., Stappenbeck C.A., Yard S., Simpson T., Nelson K.M. (2019). PTSD Is Associated with Poor Health Behavior and Greater Body Mass Index through Depression, Increasing Cardiovascular Disease and Diabetes Risk among U.S. Veterans. Prev. Med. Rep..

[149] Van den Berk-Clark C., Secrest S., Walls J., Hallberg E., Lustman P.J., Schneider F.D., Scherrer J.F. (2018). Association between Posttraumatic Stress Disorder and Lack of Exercise, Poor Diet, Obesity, and Co-Occuring Smoking: A Systematic Review and Meta-Analysis. Health Psychol..

[150] Center for Substance Abuse Treatment (US) (2014). Exhibit 1.3-4, DSM-5 Diagnostic Criteria for PTSD.

[151] Fenster R.J., Lebois L.A.M., Ressler K.J., Suh J. (2018). Brain Circuit Dysfunction in Post-Traumatic Stress Disorder: From Mouse to Man. Nat. Rev. Neurosci..

[152] Ramchand R., Schell T.L., Karney B.R., Osilla K.C., Burns R.M., Caldarone L.B. (2010). Disparate Prevalence Estimates of PTSD among Service Members Who Served in Iraq and Afghanistan: Possible Explanations. J. Trauma Stress.

[153] Scott H.R., Stevelink S.A.M., Gafoor R., Lamb D., Carr E., Bakolis I., Bhundia R., Docherty M.J., Dorrington S., Gnanapragasam S. (2023). Prevalence of Post-Traumatic Stress Disorder and Common Mental Disorders in Health-Care Workers in England during the COVID-19 Pandemic: A Two-Phase Cross-Sectional Study. Lancet Psychiatry.

[154] Javanbakht A. (2022). Addressing War Trauma in Ukrainian Refugees before It Is Too Late. Eur. J. Psychotraumatol..

[155] Al-Khudhairy M.W., Al-Mutairi A., Al Mazyad B., Al Yousef S., Hatab Alanazi S. (2022). The Association between Post-Traumatic Stress Disorder and Temporomandibular Disorders: A Systematic Review. Cureus.

[156] Castro-Vale I., Carvalho D. (2020). The Pathways between Cortisol-Related Regulation Genes and PTSD Psychotherapy. Healthcare.

[157] Somvanshi P.R., Mellon S.H., Flory J.D., Abu-Amara D., Wolkowitz O.M., Yehuda R., Jett M., Hood L., Marmar C., PTSD Systems Biology Consortium (2019). Mechanistic Inferences on Metabolic Dysfunction in Posttraumatic Stress Disorder from an Integrated Model and Multiomic Analysis: Role of Glucocorticoid Receptor Sensitivity. Am. J. Physiol. Endocrinol. Metab..

[158] Zannas A.S., Wiechmann T., Gassen N.C., Binder E.B. (2016). Gene-Stress-Epigenetic Regulation of FKBP5: Clinical and Translational Implications. Neuropsychopharmacology.

[159] Gianotti L., Belcastro S., D’Agnano S., Tassone F. (2021). The Stress Axis in Obesity and Diabetes Mellitus: An Update. Endocrines.

[160] Sohn J.-W. (2015). Network of Hypothalamic Neurons That Control Appetite. BMB Rep..

[161] Bartoli F., Crocamo C., Carrà G. (2020). Metabolic Dysfunctions in People with Post-Traumatic Stress Disorder. J. Psychopathol. Behav. Assess..

[162] Ha G.E., Cheong E. (2021). Chronic Restraint Stress Decreases the Excitability of Hypothalamic POMC Neuron and Increases Food Intake. Exp. Neurobiol..

[163] Sherin J.E., Nemeroff C.B. (2011). Post-Traumatic Stress Disorder: The Neurobiological Impact of Psychological Trauma. Dialogues Clin. Neurosci..

[164] Vuong E., Mhlongo S., Chirwa E., Lombard C., Peer N., Hemmings S.M., Abrahams N., Seedat S. (2022). Serum Adiponectin-Levels Are Predictive of Posttraumatic Stress Disorder in Women. Neurobiol. Stress.

[165] Polito R., Monda V., Nigro E., Messina A., Di Maio G., Giuliano M.T., Orrù S., Imperlini E., Calcagno G., Mosca L. (2020). The Important Role of Adiponectin and Orexin-A, Two Key Proteins Improving Healthy Status: Focus on Physical Activity. Front. Physiol..

[166] Jaksic M., Martinovic M., Gligorovic-Barhanovic N., Antunovic T., Nedovic-Vukovic M. (2021). Relationship between Insulin-like Growth Factor-1, Insulin Resistance and Metabolic Profile with Pre-Obesity and Obesity in Children. J. Pediatr. Endocrinol. Metab..

[167] Fernández de Sevilla M.E., Pignatelli J., Zegarra-Valdivia J.A., Mendez P., Nuñez A., Torres Alemán I. (2022). Insulin-like Growth Factor I Mitigates Post-Traumatic Stress by Inhibiting AMP-Kinase in Orexin Neurons. Mol. Psychiatry.

[168] Sui S.X., Pasco J.A. (2020). Obesity and Brain Function: The Brain-Body Crosstalk. Medicina.

[169] Alexander N., Kirschbaum C., Stalder T., Muehlhan M., Vogel S. (2020). No Association between FKBP5 Gene Methylation and Acute and Long-Term Cortisol Output. Transl. Psychiatry.

[170] Mourtzi N., Sertedaki A., Charmandari E. (2021). Glucocorticoid Signaling and Epigenetic Alterations in Stress-Related Disorders. Int. J. Mol. Sci..

[171] Ressler K.J., Berretta S., Bolshakov V.Y., Rosso I.M., Meloni E.G., Rauch S.L., Carlezon W.A. (2022). Post-Traumatic Stress Disorder: Clinical and Translational Neuroscience from Cells to Circuits. Nat. Rev. Neurol..

[172] Bierer L.M., Bader H.N., Daskalakis N.P., Lehrner A., Provençal N., Wiechmann T., Klengel T., Makotkine I., Binder E.B., Yehuda R. (2020). Intergenerational Effects of Maternal Holocaust Exposure on FKBP5 Methylation. Am. J. Psychiatry.

[173] Yehuda R., Daskalakis N.P., Bierer L.M., Bader H.N., Klengel T., Holsboer F., Binder E.B. (2016). Holocaust Exposure Induced Intergenerational Effects on FKBP5 Methylation. Biol. Psychiatry.

[174] Sawamura T., Klengel T., Armario A., Jovanovic T., Norrholm S.D., Ressler K.J., Andero R. (2016). Dexamethasone Treatment Leads to Enhanced Fear Extinction and Dynamic Fkbp5 Regulation in Amygdala. Neuropsychopharmacology.

[175] Bishop J.R., Lee A.M., Mills L.J., Thuras P.D., Eum S., Clancy D., Erbes C.R., Polusny M.A., Lamberty G.J., Lim K.O. (2018). Methylation of FKBP5 and SLC6A4 in Relation to Treatment Response to Mindfulness Based Stress Reduction for Posttraumatic Stress Disorder. Front. Psychiatry.

[176] Tyrka A.R., Ridout K.K., Parade S.H., Paquette A., Marsit C.J., Seifer R. (2015). Childhood Maltreatment and Methylation of FK506 Binding Protein 5 Gene (FKBP5). Dev. Psychopathol..

[177] Norris T., Hawton K., Hamilton-Shield J., Crawley E. (2017). Obesity in Adolescents with Chronic Fatigue Syndrome: An Observational Study. Arch. Dis. Child..

[178] Longui C.A., Faria C.D.C. (2009). Evaluation of Glucocorticoid Sensitivity and Its Potential Clinical Applicability. Horm. Res..

[179] Maren S. (2022). Unrelenting Fear Under Stress: Neural Circuits and Mechanisms for the Immediate Extinction Deficit. Front. Syst. Neurosci..

[180] Syed S.A., Zannas A.S., Peedicayil J., Grayson D.R., Avramopoulos D. (2021). Chapter 29—Epigenetics in Psychotherapy. Epigenetics in Psychiatry.

[181] Morgan A., Mooney K., Mc Auley M. (2016). Obesity and the Dysregulation of Fatty Acid Metabolism: Implications for Healthy Aging. Expert Rev. Endocrinol. Metab..

[182] Lushchak O., Strilbytska O., Koliada A., Storey K.B. (2022). An Orchestrating Role of Mitochondria in the Origin and Development of Post-Traumatic Stress Disorder. Front. Physiol..

[183] Corrales P., Vidal-Puig A., Medina-Gómez G. (2018). PPARs and Metabolic Disorders Associated with Challenged Adipose Tissue Plasticity. Int. J. Mol. Sci..

[184] De Spiegeleer M., De Paepe E., Van Meulebroek L., Gies I., De Schepper J., Vanhaecke L. (2021). Paediatric Obesity: A Systematic Review and Pathway Mapping of Metabolic Alterations Underlying Early Disease Processes. Mol. Med..

[185] Li G., Hu Y., Zhang W., Ding Y., Wang Y., Wang J., He Y., Lv G., von Deneen K.M., Zhao Y. (2021). Resting Activity of the Hippocampus and Amygdala in Obese Individuals Predicts Their Response to Food Cues. Addict. Biol..

[186] Hayes J.P., Vanelzakker M.B., Shin L.M. (2012). Emotion and Cognition Interactions in PTSD: A Review of Neurocognitive and Neuroimaging Studies. Front. Integr. Neurosci..

[187] Lee T.H.-Y., Yau S.-Y. (2020). From Obesity to Hippocampal Neurodegeneration: Pathogenesis and Non-Pharmacological Interventions. Int. J. Mol. Sci..

[188] Saruco E., Pleger B. (2021). A Systematic Review of Obesity and Binge Eating Associated Impairment of the Cognitive Inhibition System. Front. Nutr..

[189] Gilbertson M.W., Shenton M.E., Ciszewski A., Kasai K., Lasko N.B., Orr S.P., Pitman R.K. (2002). Smaller Hippocampal Volume Predicts Pathologic Vulnerability to Psychological Trauma. Nat. Neurosci..

[190] Alexandra Kredlow M., Fenster R.J., Laurent E.S., Ressler K.J., Phelps E.A. (2022). Prefrontal Cortex, Amygdala, and Threat Processing: Implications for PTSD. Neuropsychopharmacology.

[191] Anderberg R.H., Anefors C., Bergquist F., Nissbrandt H., Skibicka K.P. (2014). Dopamine Signaling in the Amygdala, Increased by Food Ingestion and GLP-1, Regulates Feeding Behavior. Physiol. Behav..

[192] Wong H., Singh J., Go R.M., Ahluwalia N., Guerrero-Go M.A. (2019). The Effects of Mental Stress on Non-Insulin-Dependent Diabetes: Determining the Relationship Between Catecholamine and Adrenergic Signals from Stress, Anxiety, and Depression on the Physiological Changes in the Pancreatic Hormone Secretion. Cureus.

[193] Vaiva G., Ducrocq F., Jezequel K., Averland B., Lestavel P., Brunet A., Marmar C.R. (2003). Immediate Treatment with Propranolol Decreases Posttraumatic Stress Disorder Two Months after Trauma. Biol. Psychiatry.

[194] Armstrong C., Kapolowicz M.R. (2020). A Preliminary Investigation on the Effects of Atenolol for Treating Symptoms of Anxiety. Mil. Med..

[195] Buie J.J., Watson L.S., Smith C.J., Sims-Robinson C. (2019). Obesity-Related Cognitive Impairment: The Role of Endothelial Dysfunction. Neurobiol. Dis..

[196] Willmann C., Brockmann K., Wagner R., Kullmann S., Preissl H., Schnauder G., Maetzler W., Gasser T., Berg D., Eschweiler G.W. (2020). Insulin Sensitivity Predicts Cognitive Decline in Individuals with Prediabetes. BMJ Open Diabetes Res. Care.

[197] Beaupere C., Liboz A., Fève B., Blondeau B., Guillemain G. (2021). Molecular Mechanisms of Glucocorticoid-Induced Insulin Resistance. Int. J. Mol. Sci..

[198] Ansari S., Haboubi H., Haboubi N. (2020). Adult Obesity Complications: Challenges and Clinical Impact. Ther. Adv. Endocrinol. Metab..

[199] Naser K.A., Gruber A., Thomson G.A. (2006). The Emerging Pandemic of Obesity and Diabetes: Are We Doing Enough to Prevent a Disaster?. Int. J. Clin. Pract..

[200] Rabe K., Lehrke M., Parhofer K.G., Broedl U.C. (2008). Adipokines and Insulin Resistance. Mol. Med..

[201] Engin A. (2017). Adiponectin-Resistance in Obesity. Adv. Exp. Med. Biol..

[202] Malone J.I., Hansen B.C. (2019). Does Obesity Cause Type 2 Diabetes Mellitus (T2DM)? Or Is It the Opposite?. Pediatr. Diabetes.

[203] Gidaro A., Manetti R., Delitala A.P., Salvi E., Bergamaschini L., Vidili G., Castelli R. (2021). Prothrombotic and Inflammatory Markers in Elderly Patients with Non-Alcoholic Hepatic Liver Disease before and after Weight Loss: A Pilot Study. J. Clin. Med..

[204] Buzzetti E., Pinzani M., Tsochatzis E.A. (2016). The Multiple-Hit Pathogenesis of Non-Alcoholic Fatty Liver Disease (NAFLD). Metabolism.

[205] Kim H., Lee D.S., An T.H., Park H.-J., Kim W.K., Bae K.-H., Oh K.-J. (2021). Metabolic Spectrum of Liver Failure in Type 2 Diabetes and Obesity: From NAFLD to NASH to HCC. Int. J. Mol. Sci..

[206] Chiriac S., Stanciu C., Girleanu I., Cojocariu C., Sfarti C., Singeap A.-M., Cuciureanu T., Huiban L., Muzica C.M., Zenovia S. (2021). Nonalcoholic Fatty Liver Disease and Cardiovascular Diseases: The Heart of the Matter. Can. J. Gastroenterol. Hepatol..

[207] Spinosa M., Stine J.G. (2020). Nonalcoholic Fatty Liver Disease-Evidence for a Thrombophilic State?. Curr. Pharm. Des..

[208] Vilar-Gomez E., Chalasani N. (2018). Non-Invasive Assessment of Non-Alcoholic Fatty Liver Disease: Clinical Prediction Rules and Blood-Based Biomarkers. J. Hepatol..

[209] Tokarek J., Gadzinowska J., Młynarska E., Franczyk B., Rysz J. (2021). What Is the Role of Gut Microbiota in Obesity Prevalence? A Few Words about Gut Microbiota and Its Association with Obesity and Related Diseases. Microorganisms.

[210] Cheng Z., Zhang L., Yang L., Chu H. (2022). The Critical Role of Gut Microbiota in Obesity. Front. Endocrinol..

[211] Muscogiuri G., Cantone E., Cassarano S., Tuccinardi D., Barrea L., Savastano S., Colao A. (2019). Gut Microbiota: A New Path to Treat Obesity. Int. J. Obes. Suppl..

[212] Liu B.-N., Liu X.-T., Liang Z.-H., Wang J.-H. (2021). Gut Microbiota in Obesity. World J. Gastroenterol..

[213] Beam A., Clinger E., Hao L. (2021). Effect of Diet and Dietary Components on the Composition of the Gut Microbiota. Nutrients.

[214] SPF Prévalence du Surpoids, de l’Obésité et des Facteurs de Risque Cardio-Métaboliques dans la Cohorte Constances. https://www.santepubliquefrance.fr/docs/prevalence-du-surpoids-de-l-obesite-et-des-facteurs-de-risque-cardio-metaboliques-dans-la-cohorte-constances.

[215] Hoffman D.J., Powell T.L., Barrett E.S., Hardy D.B. (2021). Developmental Origins of Metabolic Diseases. Physiol. Rev..

[216] Martins dos Santos V., Müller M., de Vos W.M. (2010). Systems Biology of the Gut: The Interplay of Food, Microbiota and Host at the Mucosal Interface. Curr. Opin. Biotechnol..

[217] Müller R., de Vos M., Sun J.Y., Sønderby I.E., Halkier B.A., Wittstock U., Jander G. (2010). Differential Effects of Indole and Aliphatic Glucosinolates on Lepidopteran Herbivores. J. Chem. Ecol..

[218] Madison A., Kiecolt-Glaser J.K. (2019). Stress, Depression, Diet, and the Gut Microbiota: Human–Bacteria Interactions at the Core of Psychoneuroimmunology and Nutrition. Curr. Opin. Behav. Sci..

[219] Foster J.A., Rinaman L., Cryan J.F. (2017). Stress & the Gut-Brain Axis: Regulation by the Microbiome. Neurobiol. Stress.

[220] Bäckhed F., Ding H., Wang T., Hooper L.V., Koh G.Y., Nagy A., Semenkovich C.F., Gordon J.I. (2004). The Gut Microbiota as an Environmental Factor That Regulates Fat Storage. Proc. Natl. Acad. Sci. USA.

[221] De La Serre C.B., Ellis C.L., Lee J., Hartman A.L., Rutledge J.C., Raybould H.E. (2010). Propensity to High-Fat Diet-Induced Obesity in Rats Is Associated with Changes in the Gut Microbiota and Gut Inflammation. Am. J. Physiol. Gastrointest. Liver Physiol..

[222] Ley R.E., Bäckhed F., Turnbaugh P., Lozupone C.A., Knight R.D., Gordon J.I. (2005). Obesity Alters Gut Microbial Ecology. Proc. Natl. Acad. Sci. USA.

[223] Turnbaugh P.J., Ley R.E., Mahowald M.A., Magrini V., Mardis E.R., Gordon J.I. (2006). An Obesity-Associated Gut Microbiome with Increased Capacity for Energy Harvest. Nature.

[224] Indiani C.M.D.S.P., Rizzardi K.F., Castelo P.M., Ferraz L.F.C., Darrieux M., Parisotto T.M. (2018). Childhood Obesity and Firmicutes/Bacteroidetes Ratio in the Gut Microbiota: A Systematic Review. Child. Obes..

[225] Bergström A., Skov T.H., Bahl M.I., Roager H.M., Christensen L.B., Ejlerskov K.T., Mølgaard C., Michaelsen K.F., Licht T.R. (2014). Establishment of Intestinal Microbiota during Early Life: A Longitudinal, Explorative Study of a Large Cohort of Danish Infants. Appl. Environ. Microbiol..

[226] Scheepers L.E.J.M., Penders J., Mbakwa C.A., Thijs C., Mommers M., Arts I.C.W. (2015). The Intestinal Microbiota Composition and Weight Development in Children: The KOALA Birth Cohort Study. Int. J. Obes..

[227] Xu P., Li M., Zhang J., Zhang T. (2012). Correlation of Intestinal Microbiota with Overweight and Obesity in Kazakh School Children. BMC Microbiol..

[228] Borgo F., Verduci E., Riva A., Lassandro C., Riva E., Morace G., Borghi E. (2017). Relative Abundance in Bacterial and Fungal Gut Microbes in Obese Children: A Case Control Study. Child. Obes..

[229] Zhang H., DiBaise J.K., Zuccolo A., Kudrna D., Braidotti M., Yu Y., Parameswaran P., Crowell M.D., Wing R., Rittmann B.E. (2009). Human Gut Microbiota in Obesity and after Gastric Bypass. Proc. Natl. Acad. Sci. USA.

[230] Gong J., Shen Y., Zhang H., Cao M., Guo M., He J., Zhang B., Xiao C. (2022). Gut Microbiota Characteristics of People with Obesity by Meta-Analysis of Existing Datasets. Nutrients.

[231] Waters J.L., Ley R.E. (2019). The Human Gut Bacteria Christensenellaceae Are Widespread, Heritable, and Associated with Health. BMC Biol..

[232] Depommier C., Everard A., Druart C., Plovier H., Van Hul M., Vieira-Silva S., Falony G., Raes J., Maiter D., Delzenne N.M. (2019). Supplementation with Akkermansia Muciniphila in Overweight and Obese Human Volunteers: A Proof-of-Concept Exploratory Study. Nat. Med..

[233] Crovesy L., Ostrowski M., Ferreira D.M.T.P., Rosado E.L., Soares-Mota M. (2017). Effect of Lactobacillus on Body Weight and Body Fat in Overweight Subjects: A Systematic Review of Randomized Controlled Clinical Trials. Int. J. Obes..

[234] Aoun A., Darwish F., Hamod N. (2020). The Influence of the Gut Microbiome on Obesity in Adults and the Role of Probiotics, Prebiotics, and Synbiotics for Weight Loss. Prev. Nutr. Food Sci..

[235] Woting A., Pfeiffer N., Loh G., Klaus S., Blaut M. (2014). Clostridium Ramosum Promotes High-Fat Diet-Induced Obesity in Gnotobiotic Mouse Models. mBio.

[236] Teixeira T.F.S., Grześkowiak Ł., Franceschini S.C.C., Bressan J., Ferreira C.L.L.F., Peluzio M.C.G. (2013). Higher Level of Faecal SCFA in Women Correlates with Metabolic Syndrome Risk Factors. Br. J. Nutr..

[237] Schéle E., Grahnemo L., Anesten F., Hallén A., Bäckhed F., Jansson J.-O. (2013). The Gut Microbiota Reduces Leptin Sensitivity and the Expression of the Obesity-Suppressing Neuropeptides Proglucagon (Gcg) and Brain-Derived Neurotrophic Factor (Bdnf) in the Central Nervous System. Endocrinology.

[238] Berding K., Bastiaanssen T.F.S., Moloney G.M., Boscaini S., Strain C.R., Anesi A., Long-Smith C., Mattivi F., Stanton C., Clarke G. (2023). Feed Your Microbes to Deal with Stress: A Psychobiotic Diet Impacts Microbial Stability and Perceived Stress in a Healthy Adult Population. Mol. Psychiatry.

[239] Paula Neto H.A., Ausina P., Gomez L.S., Leandro J.G.B., Zancan P., Sola-Penna M. (2017). Effects of Food Additives on Immune Cells as Contributors to Body Weight Gain and Immune-Mediated Metabolic Dysregulation. Front. Immunol..

[240] Riedl R.A., Burnett C.M.L., Pearson N.A., Reho J.J., Mokadem M., Edwards R.A., Kindel T.L., Kirby J.R., Grobe J.L. (2021). Gut Microbiota Represent a Major Thermogenic Biomass. Function.

[241] Natividad J.M., Agus A., Planchais J., Lamas B., Jarry A.C., Martin R., Michel M.-L., Chong-Nguyen C., Roussel R., Straube M. (2018). Impaired Aryl Hydrocarbon Receptor Ligand Production by the Gut Microbiota Is a Key Factor in Metabolic Syndrome. Cell Metab..

[242] Cuomo P., Capparelli R., Iannelli A., Iannelli D. (2022). Role of Branched-Chain Amino Acid Metabolism in Type 2 Diabetes, Obesity, Cardiovascular Disease and Non-Alcoholic Fatty Liver Disease. Int. J. Mol. Sci..

[243] Newgard C.B., An J., Bain J.R., Muehlbauer M.J., Stevens R.D., Lien L.F., Haqq A.M., Shah S.H., Arlotto M., Slentz C.A. (2009). A Branched-Chain Amino Acid-Related Metabolic Signature That Differentiates Obese and Lean Humans and Contributes to Insulin Resistance. Cell Metab..

[244] Jeganathan S., Abdullahi A., Zargar S., Maeda N., Riddell M.C., Adegoke O.A.J. (2014). Amino Acid-Induced Impairment of Insulin Sensitivity in Healthy and Obese Rats Is Reversible. Physiol. Rep..

[245] Mihalik S.J., Goodpaster B.H., Kelley D.E., Chace D.H., Vockley J., Toledo F.G.S., DeLany J.P. (2010). Increased Levels of Plasma Acylcarnitines in Obesity and Type 2 Diabetes and Identification of a Marker of Glucolipotoxicity. Obesity.

[246] Adams S.H., Hoppel C.L., Lok K.H., Zhao L., Wong S.W., Minkler P.E., Hwang D.H., Newman J.W., Garvey W.T. (2009). Plasma Acylcarnitine Profiles Suggest Incomplete Long-Chain Fatty Acid Beta-Oxidation and Altered Tricarboxylic Acid Cycle Activity in Type 2 Diabetic African-American Women. J. Nutr..

[247] Tai E.S., Tan M.L.S., Stevens R.D., Low Y.L., Muehlbauer M.J., Goh D.L.M., Ilkayeva O.R., Wenner B.R., Bain J.R., Lee J.J.M. (2010). Insulin Resistance Is Associated with a Metabolic Profile of Altered Protein Metabolism in Chinese and Asian-Indian Men. Diabetologia.

[248] Liu R., Li H., Fan W., Jin Q., Chao T., Wu Y., Huang J., Hao L., Yang X. (2017). Leucine Supplementation Differently Modulates Branched-Chain Amino Acid Catabolism, Mitochondrial Function and Metabolic Profiles at the Different Stage of Insulin Resistance in Rats on High-Fat Diet. Nutrients.

[249] Wang T.J., Larson M.G., Vasan R.S., Cheng S., Rhee E.P., McCabe E., Lewis G.D., Fox C.S., Jacques P.F., Fernandez C. (2011). Metabolite Profiles and the Risk of Developing Diabetes. Obes. Metab..

[250] Deelen J., Kettunen J., Fischer K., van der Spek A., Trompet S., Kastenmüller G., Boyd A., Zierer J., van den Akker E.B., Ala-Korpela M. (2019). A Metabolic Profile of All-Cause Mortality Risk Identified in an Observational Study of 44,168 Individuals. Nat. Commun..

[251] Ma Q., Zhou X., Hu L., Chen J., Zhu J., Shan A. (2020). Leucine and Isoleucine Have Similar Effects on Reducing Lipid Accumulation, Improving Insulin Sensitivity and Increasing the Browning of WAT in High-Fat Diet-Induced Obese Mice. Food Funct..

[252] Hu C., Li F., Duan Y., Yin Y., Kong X. (2019). Dietary Supplementation with Leucine or in Combination with Arginine Decreases Body Fat Weight and Alters Gut Microbiota Composition in Finishing Pigs. Front. Microbiol..

[253] Yao K., Duan Y., Li F., Tan B., Hou Y., Wu G., Yin Y. (2016). Leucine in Obesity: Therapeutic Prospects. Trends Pharmacol. Sci..

[254] Lin R., Li D., Xu Y., Wei M., Chen Q., Deng Y., Wen J. (2021). Chronic Cereulide Exposure Causes Intestinal Inflammation and Gut Microbiota Dysbiosis in Mice. Environ. Pollut..

[255] Kaspar D., Hastreiter S., Irmler M., Hrabé de Angelis M., Beckers J. (2020). Nutrition and Its Role in Epigenetic Inheritance of Obesity and Diabetes across Generations. Mamm. Genome.

[256] Mahmoud A.M. (2022). An Overview of Epigenetics in Obesity: The Role of Lifestyle and Therapeutic Interventions. Int. J. Mol. Sci..

[257] Wu F.-Y., Yin R.-X. (2022). Recent Progress in Epigenetics of Obesity. Diabetol. Metab. Syndr..

[258] Ouni M., Schürmann A. (2020). Epigenetic Contribution to Obesity. Mamm. Genome.

[259] Gao W., Liu J.-L., Lu X., Yang Q. (2021). Epigenetic Regulation of Energy Metabolism in Obesity. J. Mol. Cell Biol..

[260] Izquierdo A.G., Crujeiras A.B. (2019). Obesity-Related Epigenetic Changes after Bariatric Surgery. Front. Endocrinol..

[261] Wu X., Zhang Y. (2017). TET-Mediated Active DNA Demethylation: Mechanism, Function and Beyond. Nat. Rev. Genet..

[262] Arguelles A.O., Meruvu S., Bowman J.D., Choudhury M. (2016). Are Epigenetic Drugs for Diabetes and Obesity at Our Door Step?. Drug Discov. Today.

[263] Christman J.K. (2002). 5-Azacytidine and 5-Aza-2’-Deoxycytidine as Inhibitors of DNA Methylation: Mechanistic Studies and Their Implications for Cancer Therapy. Oncogene.

[264] Wu Y.-L., Lin Z.-J., Li C.-C., Lin X., Shan S.-K., Guo B., Zheng M.-H., Li F., Yuan L.-Q., Li Z.-H. (2023). Epigenetic Regulation in Metabolic Diseases: Mechanisms and Advances in Clinical Study. Signal Transduct. Target Ther..

[265] Bridgeman S.C., Ellison G.C., Melton P.E., Newsholme P., Mamotte C.D.S. (2018). Epigenetic Effects of Metformin: From Molecular Mechanisms to Clinical Implications. Diabetes Obes. Metab..

[266] Martínez J.A., Milagro F.I., Claycombe K.J., Schalinske K.L. (2014). Epigenetics in Adipose Tissue, Obesity, Weight Loss, and Diabetes. Adv. Nutr..

[267] Perfilyev A., Dahlman I., Gillberg L., Rosqvist F., Iggman D., Volkov P., Nilsson E., Risérus U., Ling C. (2017). Impact of Polyunsaturated and Saturated Fat Overfeeding on the DNA-Methylation Pattern in Human Adipose Tissue: A Randomized Controlled Trial1–3. Am. J. Clin. Nutr..

[268] Małodobra-Mazur M., Cierzniak A., Myszczyszyn A., Kaliszewski K., Dobosz T. (2021). Histone Modifications Influence the Insulin-Signaling Genes and Are Related to Insulin Resistance in Human Adipocytes. Int. J. Biochem. Cell Biol..

[269] Wu Q.-J., Zhang T.-N., Chen H.-H., Yu X.-F., Lv J.-L., Liu Y.-Y., Liu Y.-S., Zheng G., Zhao J.-Q., Wei Y.-F. (2022). The Sirtuin Family in Health and Disease. Signal Transduct. Target. Ther..

[270] Greenhill C. (2017). Non-Coding RNA: Exosomal MicroRNAs as Novel Adipokines. Nat. Rev. Genet..

[271] Panera N., Mandato C., Crudele A., Bertrando S., Vajro P., Alisi A. (2022). Genetics, Epigenetics and Transgenerational Transmission of Obesity in Children. Front. Endocrinol..

[272] Ash G.I., Kim D., Choudhury M. (2019). Promises of Nanotherapeutics in Obesity. Trends Endocrinol. Metab..

[273] Wen X., Zhang B., Wu B., Xiao H., Li Z., Li R., Xu X., Li T. (2022). Signaling Pathways in Obesity: Mechanisms and Therapeutic Interventions. Signal Transduct. Target. Ther..

[274] Walmsley R., Sumithran P. (2023). Current and Emerging Medications for the Management of Obesity in Adults. Med. J. Aust..

[275] Angelidi A.M., Belanger M.J., Kokkinos A., Koliaki C.C., Mantzoros C.S. (2022). Novel Noninvasive Approaches to the Treatment of Obesity: From Pharmacotherapy to Gene Therapy. Endocr. Rev..

[276] Müller T.D., Blüher M., Tschöp M.H., DiMarchi R.D. (2022). Anti-Obesity Drug Discovery: Advances and Challenges. Nat. Rev. Drug Discov..

[277] Kim J.D., Diano S. (2020). A Sympathetic Treatment for Obesity. Cell Metab..

[278] Haller C.A. (2004). Weight Reduction Therapies: Anorectants, Thermogenics, and Lipolytics. Principles of Gender-Specific Medicine.

[279] Weiner C.P., Mason C.P. (2019). Drugs for Pregnant and Lactating Women.

[280] Pilitsi E., Farr O.M., Polyzos S.A., Perakakis N., Nolen-Doerr E., Papathanasiou A.-E., Mantzoros C.S. (2019). Pharmacotherapy of Obesity: Available Medications and Drugs under Investigation. Metabolism.

[281] EMA Recommends Withdrawal of Obesity Drugs Containing Amfepramone. https://www.fdanews.com/articles/208218-ema-recommends-withdrawal-of-obesity-drugs-containing-amfepramone?v=preview.

[282] https://www.accessdata.fda.gov/drugsatfda_docs/label/2004/11722s029,12546s032lbl.pdf.

[283] Abramowicz M.J., Van Haecke P., Demedts M., Delcroix M. (2003). Primary Pulmonary Hypertension after Amfepramone (Diethylpropion) with BMPR2 Mutation. Eur. Respir. J..

[284] Kalyanasundar B., Perez C.I., Arroyo B., Moreno M.G., Gutierrez R. (2020). The Appetite Suppressant D-Norpseudoephedrine (Cathine) Acts via D1/D2-like Dopamine Receptors in the Nucleus Accumbens Shell. Front. Neurosci..

[285] Hauner H., Hastreiter L., Werdier D., Chen-Stute A., Scholze J., Blüher M. (2017). Efficacy and Safety of Cathine (nor-Pseudoephedrine) in the Treatment of Obesity: A Randomized Dose-Finding Study. Obes. Facts.

[286] Li M.-F., Cheung B.M. (2011). Rise and Fall of Anti-Obesity Drugs. World J. Diabetes.

[287] Tchang B.G., Aras M., Kumar R.B., Aronne L.J. (2021). Pharmacologic Treatment of Overweight and Obesity in Adults.

[288] Ionică F.E., Negreș S., Șeremet O.C., Chiriță C. (2016). Pharmacotherapy in the Treatment of Obesity. Rom. J. Diabetes Nutr. Metab. Dis..

[289] Chakhtoura M., Haber R., Ghezzawi M., Rhayem C., Tcheroyan R., Mantzoros C.S. (2023). Pharmacotherapy of Obesity: An Update on the Available Medications and Drugs under Investigation. EClinicalMedicine.

[290] Lafferty R.A., Flatt P.R., Irwin N. (2022). Is Polypharmacy the Future for Pharmacological Management of Obesity?. Curr. Opin. Endocr. Metab. Res..

[291] Finlin B.S., Memetimin H., Zhu B., Confides A.L., Vekaria H.J., El Khouli R.H., Johnson Z.R., Westgate P.M., Chen J., Morris A.J. (2020). The Β3-Adrenergic Receptor Agonist Mirabegron Improves Glucose Homeostasis in Obese Humans. J. Clin. Investig..

[292] https://go.drugbank.com/drugs/DB08893/clinical_trials?conditions=DBCOND0015947&phase=2&purpose=basic_science&status=completed.

[293] O’Mara A.E., Johnson J.W., Linderman J.D., Brychta R.J., McGehee S., Fletcher L.A., Fink Y.A., Kapuria D., Cassimatis T.M., Kelsey N. (2020). Chronic Mirabegron Treatment Increases Human Brown Fat, HDL Cholesterol, and Insulin Sensitivity. J. Clin. Investig..

[294] Kosmalski M., Deska K., Bąk B., Różycka-Kosmalska M., Pietras T. (2023). Pharmacological Support for the Treatment of Obesity-Present and Future. Healthcare.

[295] Dawood O., El-Zawahry A. (2022). Mirabegron.

[296] Caron A., Jane Michael N. (2021). New Horizons: Is Obesity a Disorder of Neurotransmission?. J. Clin. Endocrinol. Metab..

[297] Zonisamide SR Plus Bupropion SR Combination Therapy in Subjects with Obesity. https://www.clinicaltrials.gov/ct2/show/NCT00709371.

[298] Singh R., Kumar B., Kuhad A., Kuhad A. (2018). Tesofensine. Triple Monoamine Reuptake Inhibitor of Dopamine, Norepinephrine and Serotonin; Treatment of Obesity. Drugs Future.

[299] Rebello C.J., Greenway F.L. (2020). Obesity Medications in Development. Expert Opin. Investig. Drugs.

[300] Leddy J.J., Epstein L.H., Jaroni J.L., Roemmich J.N., Paluch R.A., Goldfield G.S., Lerman C. (2004). Influence of Methylphenidate on Eating in Obese Men. Obes. Res..

[301] Mellström E., Forsman C., Engh L., Hallerbäck M.U., Wikström S. (2020). Methylphenidate and Reduced Overweight in Children with ADHD. J. Atten. Disord..

[302] Khajehpiri Z., Mahmoudi-Gharaei J., Faghihi T., Karimzadeh I., Khalili H., Mohammadi M. (2014). Adverse Reactions of Methylphenidate in Children with Attention Deficit-Hyperactivity Disorder: Report from a Referral Center. J. Res. Pharm. Pract..

[303] Surapaneni P., Vinales K.L., Najib M.Q., Chaliki H.P. (2011). Valvular Heart Disease with the Use of Fenfluramine-Phentermine. Tex. Heart Inst. J..

[304] Belohlávková S., Simák J., Kokesová A., Hnilicková O., Hampl V. (2001). Fenfluramine-Induced Pulmonary Vasoconstriction: Role of Serotonin Receptors and Potassium Channels. J. Appl. Physiol..

[305] Kim K.K. (2019). Understanding the Mechanism of Action and Clinical Implications of Anti-Obesity Drugs Recently Approved in Korea. Korean J. Fam. Med..

[306] Woloshin S., Schwartz L.M. (2014). The New Weight-Loss Drugs, Lorcaserin and Phentermine-Topiramate: Slim Pickings?. JAMA Intern. Med..

[307] Martin C.K., Redman L.M., Zhang J., Sanchez M., Anderson C.M., Smith S.R., Ravussin E. (2011). Lorcaserin, a 5-HT(2C) Receptor Agonist, Reduces Body Weight by Decreasing Energy Intake without Influencing Energy Expenditure. J. Clin. Endocrinol. Metab..

[308] Radius Health, Inc Announces Acquisition of Orphan Disease Program. https://www.biospace.com/article/releases/radius-health-inc-announces-acquisition-of-orphan-disease-program/.

[309] De Ceglia M., Decara J., Gaetani S., Rodríguez de Fonseca F. (2021). Obesity as a Condition Determined by Food Addiction: Should Brain Endocannabinoid System Alterations Be the Cause and Its Modulation the Solution?. Pharmaceuticals.

[310] Cinar R., Iyer M.R., Kunos G. (2020). The Therapeutic Potential of Second and Third Generation CB1R Antagonists. Pharmacol. Ther..

[311] Kale V.P., Gibbs S., Taylor J.A., Zmarowski A., Novak J., Patton K., Sparrow B., Gorospe J., Anand S., Cinar R. (2019). Preclinical Toxicity Evaluation of JD5037, a Peripherally Restricted CB1 Receptor Inverse Agonist, in Rats and Dogs for Treatment of Nonalcoholic Steatohepatitis. Regul. Toxicol. Pharmacol..

[312] A Phase 3 Extension Study of RAD011 (Cannabidiol Oral Solution) in Patients with Prader-Willi Syndrome. https://clinicaltrials.gov/ct2/show/NCT05387798.

[313] Huestis M.A., Solimini R., Pichini S., Pacifici R., Carlier J., Busardò F.P. (2019). Cannabidiol Adverse Effects and Toxicity. Curr. Neuropharmacol..

[314] Nicolucci A., Maffeis C. (2022). The Adolescent with Obesity: What Perspectives for Treatment?. Ital. J. Pediatr..

[315] Singh G., Krauthamer M., Bjalme-Evans M. (2022). Wegovy (Semaglutide): A New Weight Loss Drug for Chronic Weight Management. J. Investig. Med..

[316] Chao A.M., Tronieri J.S., Amaro A., Wadden T.A. (2023). Semaglutide for the Treatment of Obesity. Trends Cardiovasc. Med..

[317] Bonora E., Frias J.P., Tinahones F.J., Van J., Malik R.E., Yu Z., Mody R., Bethel A., Kwan A.Y.M., Cox D.A. (2021). Effect of Dulaglutide 3.0 and 4.5 Mg on Weight in Patients with Type 2 Diabetes: Exploratory Analyses of AWARD-11. Diabetes Obes. Metab..

[318] Collins L., Costello R.A. (2023). Glucagon-like Peptide-1 Receptor Agonists.

[319] The Effect of SHR20004 (Noiiglutide) on Body Weight in Obese Subjects without Diabetes. https://clinicaltrials.gov/ct2/show/NCT04799327.

[320] Kawai T., Sun B., Yoshino H., Feng D., Suzuki Y., Fukazawa M., Nagao S., Wainscott D.B., Showalter A.D., Droz B.A. (2020). Structural Basis for GLP-1 Receptor Activation by LY3502970, an Orally Active Nonpeptide Agonist. Proc. Natl. Acad. Sci. USA.

[321] Effects of XW003 Versus Liraglutide on Body Weight of Adult Participants with Obesity. https://clinicaltrials.gov/ct2/show/NCT05111912.

[322] Seo Y.-G. (2021). Side Effects Associated with Liraglutide Treatment for Obesity as Well as Diabetes. J. Obes. Metab. Syndr..

[323] Colin I.M., Gérard K.M. (2022). Once-Weekly 2.4 Mg Semaglutide for Weight Management in Obesity: A Game Changer?. touchREV. Endocrinol..

[324] Pinto L.C., Rados D.V., Barkan S.S., Leitão C.B., Gross J.L. (2018). Dipeptidyl Peptidase-4 Inhibitors, Pancreatic Cancer and Acute Pancreatitis: A Meta-Analysis with Trial Sequential Analysis. Sci. Rep..

[325] Fukuda M. (2021). The Role of GIP Receptor in the CNS for the Pathogenesis of Obesity. Diabetes.

[326] Min X., Yie J., Wang J., Chung B.C., Huang C.-S., Xu H., Yang J., Deng L., Lin J., Chen Q. (2020). Molecular Mechanism of an Antagonistic Antibody against Glucose-Dependent Insulinotropic Polypeptide Receptor. mAbs.

[327] Killion E.A., Wang J., Yie J., Shi S.D.-H., Bates D., Min X., Komorowski R., Hager T., Deng L., Atangan L. (2018). Anti-Obesity Effects of GIPR Antagonists Alone and in Combination with GLP-1R Agonists in Preclinical Models. Sci. Transl. Med..

[328] Nakamura T., Tanimoto H., Okamoto M., Takeuchi M., Tsubamoto Y., Noda H. (2021). GIP Receptor Antagonist, SKL-14959 Indicated Alteration of the Lipids Metabolism to Catabolism by the Inhibition of Plasma LPL Activity, Resulting in the Suppression of Weight Gain on Diets-Induced Obesity Mice. Diabetes Metab. Syndr. Obes..

[329] Samms R.J., Cosgrove R., Snider B.M., Furber E.C., Droz B.A., Briere D.A., Dunbar J., Dogra M., Alsina-Fernandez J., Borner T. (2022). GIPR Agonism Inhibits PYY-Induced Nausea-like Behavior. Diabetes.

[330] Borner T., Geisler C.E., Fortin S.M., Cosgrove R., Alsina-Fernandez J., Dogra M., Doebley S., Sanchez-Navarro M.J., Leon R.M., Gaisinsky J. (2021). GIP Receptor Agonism Attenuates GLP-1 Receptor Agonist-Induced Nausea and Emesis in Preclinical Models. Diabetes.

[331] Zhao F., Zhou Q., Cong Z., Hang K., Zou X., Zhang C., Chen Y., Dai A., Liang A., Ming Q. (2022). Structural Insights into Multiplexed Pharmacological Actions of Tirzepatide and Peptide 20 at the GIP, GLP-1 or Glucagon Receptors. Nat. Commun..

[332] Kleinert M., Sachs S., Habegger K.M., Hofmann S.M., Müller T.D. (2019). Glucagon Regulation of Energy Expenditure. Int. J. Mol. Sci..

[333] Hope D.C.D., Vincent M.L., Tan T.M.M. (2021). Striking the Balance: GLP-1/Glucagon Co-Agonism as a Treatment Strategy for Obesity. Front. Endocrinol..

[334] Campbell J.E. (2021). Targeting the GIPR for Obesity: To Agonize or Antagonize? Potential Mechanisms. Mol. Metab..

[335] Chavda V.P., Ajabiya J., Teli D., Bojarska J., Apostolopoulos V. (2022). Tirzepatide, a New Era of Dual-Targeted Treatment for Diabetes and Obesity: A Mini-Review. Molecules.

[336] Jastreboff A.M., Aronne L.J., Ahmad N.N., Wharton S., Connery L., Alves B., Kiyosue A., Zhang S., Liu B., Bunck M.C. (2022). Tirzepatide Once Weekly for the Treatment of Obesity. N. Engl. J. Med..

[337] Holst J.J., Rosenkilde M.M. (2020). GIP as a Therapeutic Target in Diabetes and Obesity: Insight from Incretin Co-Agonists. J. Clin. Endocrinol. Metab..

[338] GMA106. https://www.gmaxbiopharm.com/wap_product_detailen/id/6.html.

[339] Jepsen M.M., Christensen M.B. (2021). Emerging Glucagon-like Peptide 1 Receptor Agonists for the Treatment of Obesity. Expert Opin. Emerg. Drugs.

[340] Mishra R., Raj R., Elshimy G., Zapata I., Kannan L., Majety P., Edem D., Correa R. (2023). Adverse Events Related to Tirzepatide. J. Endocr. Soc..

[341] Innovent Announces First Participant Dosed in a Phase 3 Clinical Study (GLORY-1) of Mazdutide (IBI362) in Chinese Adults with Overweight or Obesity. https://www.prnewswire.com/news-releases/innovent-announces-first-participant-dosed-in-a-phase-3-clinical-study-glory-1-of-mazdutide-ibi362-in-chinese-adults-with-overweight-or-obesity-301676778.html.

[342] Ji L., Gao L., Jiang H., Yang J., Yu L., Wen J., Cai C., Deng H., Feng L., Song B. (2022). Safety and Efficacy of a GLP-1 and Glucagon Receptor Dual Agonist Mazdutide (IBI362) 9 Mg and 10 Mg in Chinese Adults with Overweight or Obesity: A Randomised, Placebo-Controlled, Multiple-Ascending-Dose Phase 1b Trial. eClinicalMedicine.

[343] Kosinski J.R., Hubert J., Carrington P.E., Chicchi G.G., Mu J., Miller C., Cao J., Bianchi E., Pessi A., Sinharoy R. (2012). The Glucagon Receptor Is Involved in Mediating the Body Weight-Lowering Effects of Oxyntomodulin. Obesity.

[344] Nahra R., Wang T., Gadde K.M., Oscarsson J., Stumvoll M., Jermutus L., Hirshberg B., Ambery P. (2021). Effects of Cotadutide on Metabolic and Hepatic Parameters in Adults With Overweight or Obesity and Type 2 Diabetes: A 54-Week Randomized Phase 2b Study. Diabetes Care.

[345] Hong S.M., Ko J.-K., Moon J.-J., Kim Y.-R. (2021). Oxytocin: A Potential Therapeutic for Obesity. J. Obes. Metab. Syndr..

[346] Doggrell S.A. (2023). Is Retatrutide (LY3437943), a GLP-1, GIP, and Glucagon Receptor Agonist a Step Forward in the Treatment of Diabetes and Obesity?. Expert Opin. Investig. Drugs.

[347] Castillo E.J., Delgado-Aros S., Camilleri M., Burton D., Stephens D., O’Connor-Semmes R., Walker A., Shachoy-Clark A., Zinsmeister A.R. (2004). Effect of Oral CCK-1 Agonist GI181771X on Fasting and Postprandial Gastric Functions in Healthy Volunteers. Am. J. Physiol. Gastrointest. Liver Physiol..

[348] Roses A.D. (2009). Stimulation of Cholecystokinin-A Receptors with Gl181771X: A Failed Clinical Trial That Did Not Test the Pharmacogenetic Hypothesis for Reduction of Food Intake. Clin. Pharmacol. Ther..

[349] Nyborg N.C.B., Kirk R.K., de Boer A.S., Andersen D.W., Bugge A., Wulff B.S., Thorup I., Clausen T.R. (2020). Cholecystokinin-1 Receptor Agonist Induced Pathological Findings in the Exocrine Pancreas of Non-Human Primates. Toxicol. Appl. Pharmacol..

[350] Jastreboff A.M., Kushner R.F. (2023). New Frontiers in Obesity Treatment: GLP-1 and Nascent Nutrient-Stimulated Hormone-Based Therapeutics. Annu. Rev. Med..

[351] Dengler D.G., Sun Q., Harikumar K.G., Miller L.J., Sergienko E.A. (2022). Screening for Positive Allosteric Modulators of Cholecystokinin Type 1 Receptor Potentially Useful for Management of Obesity. SLAS Discov..

[352] Christoffersen B.Ø., Skyggebjerg R.B., Bugge A., Kirk R.K., Vestergaard B., Uldam H.K., Fels J.J., Pyke C., Sensfuss U., Sanfridson A. (2020). Long-Acting CCK Analogue NN9056 Lowers Food Intake and Body Weight in Obese Göttingen Minipigs. Int. J. Obes..

[353] Holzer P., Reichmann F., Farzi A. (2012). Neuropeptide Y, Peptide YY and Pancreatic Polypeptide in the Gut-Brain Axis. Neuropeptides.

[354] Behary P., Tharakan G., Alexiadou K., Johnson N., Wewer Albrechtsen N.J., Kenkre J., Cuenco J., Hope D., Anyiam O., Choudhury S. (2019). Combined GLP-1, Oxyntomodulin, and Peptide YY Improves Body Weight and Glycemia in Obesity and Prediabetes/Type 2 Diabetes: A Randomized, Single-Blinded, Placebo-Controlled Study. Diabetes Care.

[355] Rangwala S.M., D’Aquino K., Zhang Y.-M., Bader L., Edwards W., Zheng S., Eckardt A., Lacombe A., Pick R., Moreno V. (2019). A Long-Acting PYY3-36 Analog Mediates Robust Anorectic Efficacy with Minimal Emesis in Nonhuman Primates. Cell Metab..

[356] Zhang W., Cline M.A., Gilbert E.R. (2014). Hypothalamus-Adipose Tissue Crosstalk: Neuropeptide Y and the Regulation of Energy Metabolism. Nutr. Metab..

[357] A Study to Determine the Effects of MK0557 in Obese Subjects (0557-006)(COMPLETED). https://clinicaltrials.gov/ct2/show/NCT00533598.

[358] Yulyaningsih E., Zhang L., Herzog H., Sainsbury A. (2011). NPY Receptors as Potential Targets for Anti-Obesity Drug Development. Br. J. Pharmacol..

[359] Trapp C.M., Censani M. (2023). Setmelanotide: A Promising Advancement for Pediatric Patients with Rare Forms of Genetic Obesity. Curr. Opin. Endocrinol. Diabetes Obes..

[360] EMA EU/3/19/2192. https://www.ema.europa.eu/en/medicines/human/orphan-designations/eu-3-19-2192.

[361] Yeo G.S.H., Chao D.H.M., Siegert A.-M., Koerperich Z.M., Ericson M.D., Simonds S.E., Larson C.M., Luquet S., Clarke I., Sharma S. (2021). The Melanocortin Pathway and Energy Homeostasis: From Discovery to Obesity Therapy. Mol. Metab..

[362] Hussain A., Farzam K. (2023). Setmelanotide.

[363] Altabas V., Zjačić-Rotkvić V. (2015). Anti-Ghrelin Antibodies in Appetite Suppression: Recent Advances in Obesity Pharmacotherapy. ImmunoTargets Ther..

[364] Delporte C. (2012). Recent Advances in Potential Clinical Application of Ghrelin in Obesity. J. Obes..

[365] A Study of Oral ARD-101 in Patients with Prader-Willi Syndrome. https://clinicaltrials.gov/ct2/show/NCT05153434.

[366] A Study of GLWL-01 in Patients with Prader-Willi Syndrome. https://clinicaltrials.gov/ct2/show/NCT03274856.

[367] (2019). Kernel Networks Inc. Liver-Enriched Antimicrobial Peptide 2. Case Med. Res..

[368] Obradovic M., Sudar-Milovanovic E., Soskic S., Essack M., Arya S., Stewart A.J., Gojobori T., Isenovic E.R. (2021). Leptin and Obesity: Role and Clinical Implication. Front. Endocrinol..

[369] Hukshorn C.J., van Dielen F.M.H., Buurman W.A., Westerterp-Plantenga M.S., Campfield L.A., Saris W.H.M. (2002). The Effect of Pegylated Recombinant Human Leptin (PEG-OB) on Weight Loss and Inflammatory Status in Obese Subjects. Int. J. Obes..

[370] Chellappa K., Perron I.J., Naidoo N., Baur J.A. (2019). The Leptin Sensitizer Celastrol Reduces Age-Associated Obesity and Modulates Behavioral Rhythms. Aging Cell.

[371] Shao J., Li C., Bai L., Ni X., Ge S., Zhang J., Zhao H. (2022). Recent Evidence in Support of Traditional Chinese Medicine to Restore Normal Leptin Function in Simple Obesity. Heliyon.

[372] Chan J.L., Koda J., Heilig J.S., Cochran E.K., Gorden P., Oral E.A., Brown R.J. (2016). Immunogenicity Associated with Metreleptin Treatment in Patients with Obesity or Lipodystrophy. Clin. Endocrinol..

[373] Ravussin E., Smith S.R., Mitchell J.A., Shringarpure R., Shan K., Maier H., Koda J.E., Weyer C. (2009). Enhanced Weight Loss with Pramlintide/Metreleptin: An Integrated Neurohormonal Approach to Obesity Pharmacotherapy. Obesity.

[374] Mathiesen D.S., Lund A., Vilsbøll T., Knop F.K., Bagger J.I. (2020). Amylin and Calcitonin: Potential Therapeutic Strategies to Reduce Body Weight and Liver Fat. Front. Endocrinol..

[375] Gydesen S., Andreassen K.V., Hjuler S.T., Christensen J.M., Karsdal M.A., Henriksen K. (2016). KBP-088, a Novel DACRA with Prolonged Receptor Activation, Is Superior to Davalintide in Terms of Efficacy on Body Weight. Am. J. Physiol. Endocrinol. Metab..

[376] Yokote K., Sano M., Tsumiyama I., Keefe D. (2020). Dose-Dependent Reduction in Body Weight with LIK066 (Licogliflozin) Treatment in Japanese Patients with Obesity. Diabetes Obes. Metab..

[377] Bays H.E., Kozlovski P., Shao Q., Proot P., Keefe D. (2020). Licogliflozin, a Novel SGLT1 and 2 Inhibitor: Body Weight Effects in a Randomized Trial in Adults with Overweight or Obesity. Obesity.

[378] Cefalo C.M.A., Cinti F., Moffa S., Impronta F., Sorice G.P., Mezza T., Pontecorvi A., Giaccari A. (2019). Sotagliflozin, the First Dual SGLT Inhibitor: Current Outlook and Perspectives. Cardiovasc. Diabetol..

[379] Single and Multiple Ascending Dose Study of AMG 133 in Participants with Obesity. https://clinicaltrials.gov/ct2/show/NCT04478708.

[380] Study to Assess Safety, Tolerability and Efficacy of SC Administered MBL949 in Obese Participants with or without T2DM. https://clinicaltrials.gov/ct2/show/NCT05199090.

[381] Xu W., Zhang J., Xiao J. (2021). Roflumilast Suppresses Adipogenic Differentiation via AMPK Mediated Pathway. Front. Endocrinol..

[382] The Possible Effects of Roflumilast on Obesity Related Disorders. https://clinicaltrials.gov/ct2/show/NCT04800172.

[383] Joo H., Han D., Lee J.H., Rhee C.K. (2018). Incidence of Adverse Effects and Discontinuation Rate between Patients Receiving 250 Micrograms and 500 Micrograms of Roflumilast: A Comparative Study. Tuberc. Respir. Dis..

[384] Iside C., Scafuro M., Nebbioso A., Altucci L. (2020). SIRT1 Activation by Natural Phytochemicals: An Overview. Front. Pharmacol..

[385] Zemel M.B., Kolterman O., Rinella M., Vuppalanchi R., Flores O., Barritt A.S., Siddiqui M., Chalasani N. (2019). Randomized Controlled Trial of a Leucine-Metformin-Sildenafil Combination (NS-0200) on Weight and Metabolic Parameters. Obesity.

[386] Sonoda J., Chen M.Z., Baruch A. (2017). FGF21-Receptor Agonists: An Emerging Therapeutic Class for Obesity-Related Diseases. Horm. Mol. Biol. Clin. Investig..

[387] Shao W., Jin T. (2022). Hepatic Hormone FGF21 and Its Analogues in Clinical Trials. Chronic Dis. Transl. Med..

[388] Kaufman A., Abuqayyas L., Denney W.S., Tillman E.J., Rolph T. (2020). AKR-001, an Fc-FGF21 Analog, Showed Sustained Pharmacodynamic Effects on Insulin Sensitivity and Lipid Metabolism in Type 2 Diabetes Patients. Cell Rep. Med..

[389] Yan J., Nie Y., Cao J., Luo M., Yan M., Chen Z., He B. (2021). The Roles and Pharmacological Effects of FGF21 in Preventing Aging-Associated Metabolic Diseases. Front. Cardiovasc. Med..

[390] Chen M.Z., Chang J.C., Zavala-Solorio J., Kates L., Thai M., Ogasawara A., Bai X., Flanagan S., Nunez V., Phamluong K. (2017). FGF21 Mimetic Antibody Stimulates UCP1-Independent Brown Fat Thermogenesis via FGFR1/ΒKlotho Complex in Non-Adipocytes. Mol. Metab..

[391] Prikhodko V.A., Bezborodkina N.N., Okovityi S.V. (2022). Pharmacotherapy for Non-Alcoholic Fatty Liver Disease: Emerging Targets and Drug Candidates. Biomedicines.

[392] Panzitt K., Zollner G., Marschall H.-U., Wagner M. (2022). Recent Advances on FXR-Targeting Therapeutics. Mol. Cell. Endocrinol..

[393] Ge H., Zhang J., Gong Y., Gupte J., Ye J., Weiszmann J., Samayoa K., Coberly S., Gardner J., Wang H. (2014). Fibroblast Growth Factor Receptor 4 (FGFR4) Deficiency Improves Insulin Resistance and Glucose Metabolism under Diet-Induced Obesity Conditions. J. Biol. Chem..

[394] Jin L., Yang R., Geng L., Xu A. (2023). Fibroblast Growth Factor–Based Pharmacotherapies for the Treatment of Obesity-Related Metabolic Complications. Annu. Rev. Pharmacol. Toxicol..

[395] Dhuri K., Bechtold C., Quijano E., Pham H., Gupta A., Vikram A., Bahal R. (2020). Antisense Oligonucleotides: An Emerging Area in Drug Discovery and Development. J. Clin. Med..

[396] Isis Initiates Phase 1 Clinical Trial of ISIS-FGFR4Rx a Peripherally Acting Drug to Treat Obesity. https://ir.ionispharma.com/news-releases/news-release-details/isis-initiates-phase-1-clinical-trial-isis-fgfr4rx-peripherally.

[397] Yang L., Chang C.-C., Sun Z., Madsen D., Zhu H., Padkjær S.B., Wu X., Huang T., Hultman K., Paulsen S.J. (2017). GFRAL Is the Receptor for GDF15 and Is Required for the Anti-Obesity Effects of the Ligand. Nat. Med..

[398] Breit S.N., Brown D.A., Tsai V.W.-W. (2021). The GDF15-GFRAL Pathway in Health and Metabolic Disease: Friend or Foe?. Annu. Rev. Physiol..

[399] Xie H., Yepuri N., Meng Q., Dhawan R., Leech C.A., Chepurny O.G., Holz G.G., Cooney R.N. (2020). Therapeutic Potential of A7 Nicotinic Acetylcholine Receptor Agonists to Combat Obesity, Diabetes, and Inflammation. Rev. Endocr. Metab. Disord..

[400] Heymsfield S.B., Coleman L.A., Miller R., Rooks D.S., Laurent D., Petricoul O., Praestgaard J., Swan T., Wade T., Perry R.G. (2021). Effect of Bimagrumab vs Placebo on Body Fat Mass among Adults with Type 2 Diabetes and Obesity: A Phase 2 Randomized Clinical Trial. JAMA Netw. Open.

[401] Zhang J., Wu X., Zhong B., Liao Q., Wang X., Xie Y., He X. (2023). Review on the Diverse Biological Effects of Glabridin. Drug Des. Devel. Ther..

[402] Choi L.S., Jo I.G., Kang K.S., Im J.H., Kim J., Kim J., Chung J.W., Yoo S.-K. (2021). Discovery and Preclinical Efficacy of HSG4112, a Synthetic Structural Analog of Glabridin, for the Treatment of Obesity. Int. J. Obes..

[403] Mansor F., Gu H.F., Ostenson C.-G., Mannerås-Holm L., Stener-Victorin E., Wan Mohamud W.N. (2013). Labisia Pumila Upregulates Peroxisome Proliferator-Activated Receptor Gamma Expression in Rat Adipose Tissues and 3T3-L1 Adipocytes. Adv. Pharmacol. Sci..

[404] Turck D., Bohn T., Castenmiller J., De Henauw S., Hirsch-Ernst K.I., Maciuk A., Mangelsdorf I., McArdle H.J., Naska A., EFSA Panel on Nutrition, Novel Foods and Food Allergens (NDA) (2022). Safety of an Aqueous Ethanolic Extract of Labisia Pumila as a Novel Food Pursuant to Regulation (EU) 2015/2283. EFSA J..

[405] Dose-Ranging Study of SKF7TM for Obesity. https://clinicaltrials.gov/ct2/show/NCT04557267.

[406] Iwaza R., Wasfy R.M., Dubourg G., Raoult D., Lagier J.-C. (2022). Akkermansia Muciniphila: The State of the Art, 18 Years after Its First Discovery. Front. Gastroenterol..

[407] Effect of Akkermansia Muciniphila WST01 Strain in Overweight or Obese Patients with Type 2 Diabetes. https://clinicaltrials.gov/ct2/show/NCT04797442.

[408] Wang K., Wu W., Wang Q., Yang L., Bian X., Jiang X., Lv L., Yan R., Xia J., Han S. (2022). The Negative Effect of Akkermansia Muciniphila-Mediated Post-Antibiotic Reconstitution of the Gut Microbiota on the Development of Colitis-Associated Colorectal Cancer in Mice. Front. Microbiol..

[409] Diez-Echave P., Vezza T., Algieri F., Ruiz-Malagón A.J., Hidalgo-García L., García F., Morón R., Sánchez M., Toral M., Romero M. (2022). The Melatonergic Agonist Agomelatine Ameliorates High Fat Diet-Induced Obesity in Mice through the Modulation of the Gut Microbiome. Biomed. Pharmacother..

[410] Guan Q., Wang Z., Cao J., Dong Y., Chen Y. (2021). Mechanisms of Melatonin in Obesity: A Review. Int. J. Mol. Sci..

[411] Cherngwelling R., Pengrattanachot N., Swe M.T., Thongnak L., Promsan S., Phengpol N., Sutthasupha P., Lungkaphin A. (2021). Agomelatine Protects against Obesity-Induced Renal Injury by Inhibiting Endoplasmic Reticulum Stress/Apoptosis Pathway in Rats. Toxicol. Appl. Pharmacol..

[412] Xu L., Li D., Li H., Zhang O., Huang Y., Shao H., Wang Y., Cai S., Zhu Y., Jin S. (2022). Suppression of Obesity by Melatonin through Increasing Energy Expenditure and Accelerating Lipolysis in Mice Fed a High-Fat Diet. Nutr. Diabetes.

[413] Williams W.P., McLin D.E., Dressman M.A., Neubauer D.N. (2016). Comparative Review of Approved Melatonin Agonists for the Treatment of Circadian Rhythm Sleep-wake Disorders. Pharmacotherapy.

[414] Partonen T. (2023). Medication Effects. Encyclopedia of Sleep and Circadian Rhythms.

[415] Zizzo J., Reddy R., Kulkarni N., Blachman-Braun R., Ramasamy R. (2022). Impact of Low-Dose Melatonin Supplementation on Testosterone Levels in U.s. Adult Males. Urology.

[416] Study to Evaluate the Safety, Tolerability, and Efficacy of ASC41 in Adults with NASH. https://clinicaltrials.gov/ct2/show/NCT05118360.

[417] Perra A., Kowalik M.A., Cabras L., Runfola M., Sestito S., Migliore C., Giordano S., Chiellini G., Rapposelli S., Columbano A. (2020). Potential Role of Two Novel Agonists of Thyroid Hormone Receptor-β on Liver Regeneration. Cell Prolif..

[418] Double-Blind, Placebo Controlled, Phase 3 Trial of ZGN-440 (Beloranib) in Obese Subjects With Prader-Willi Syndrome—Full Text View—Clinicaltrials.gov. https://clinicaltrials.gov/ct2/show/NCT02179151.

[419] McCandless S.E., Yanovski J.A., Miller J., Fu C., Bird L.M., Salehi P., Chan C.L., Stafford D., Abuzzahab M.J., Viskochil D. (2017). Effects of MetAP2 Inhibition on Hyperphagia and Body Weight in Prader-Willi Syndrome: A Randomized, Double-Blind, Placebo-Controlled Trial. Diabetes Obes. Metab..

[420] Pang K.-L., Chin K.-Y. (2019). The Role of Tocotrienol in Protecting against Metabolic Diseases. Molecules.

[421] Tocotrienols for Obesity of Postmenopausal Women—Full Text View—Clinicaltrials.gov. https://clinicaltrials.gov/ct2/show/NCT03705845.

[422] Denison H., Nilsson C., Kujacic M., Löfgren L., Karlsson C., Knutsson M., Eriksson J.W. (2013). Proof of Mechanism for the DGAT1 Inhibitor AZD7687: Results from a First-Time-in-Human Single-Dose Study. Diabetes Obes. Metab..

[423] Amin N.B., Saxena A.R., Somayaji V., Dullea R. (2023). Inhibition of Diacylglycerol Acyltransferase 2 versus Diacylglycerol Acyltransferase 1: Potential Therapeutic Implications of Pharmacology. Clin. Ther..

[424] Mochida T., Take K., Maki T., Nakakariya M., Adachi R., Sato K., Kitazaki T., Takekawa S. (2020). Inhibition of MGAT2 Modulates Fat-Induced Gut Peptide Release and Fat Intake in Normal Mice and Ameliorates Obesity and Diabetes in Ob/Ob Mice Fed on a High-Fat Diet. FEBS Open Bio.

[425] McFie P.J., Patel A., Stone S.J. (2022). The Monoacylglycerol Acyltransferase Pathway Contributes to Triacylglycerol Synthesis in HepG2 Cells. Sci. Rep..

[426] Okuma C., Ohta T., Tadaki H., Hamada H., Oda T., Taniuchi H., Yamanaka K., Ishii Y., Ohe Y., Yata S. (2015). JTP-103237, a Novel Monoacylglycerol Acyltransferase Inhibitor, Modulates Fat Absorption and Prevents Diet-Induced Obesity. Eur. J. Pharmacol..

[427] Yang M., Nickels J.T. (2015). MOGAT2: A New Therapeutic Target for Metabolic Syndrome. Diseases.

[428] Perreault M., Erbe D.V., Tobin J.F. (2008). PPAR δ Agonism for the Treatment of Obesity and Associated Disorders: Challenges and Opportunities. PPAR Res..

[429] Greenway F.L., Aronne L.J., Raben A., Astrup A., Apovian C.M., Hill J.O., Kaplan L.M., Fujioka K., Matejkova E., Svacina S. (2019). A Randomized Double-Blind, Placebo-Controlled Study of Gelesis100: A Novel Nonsystemic Oral Hydrogel for Weight Loss. Obesity.

[430] Giruzzi N. (2020). Plenity (Oral Superabsorbent Hydrogel). Clin. Diabetes.

[431] Urban L.E., Audet D., Ron E.S., Sannino A., Zohar Y., Demitri C., Panteca E., Surano I., Heshmati H.M. (2018). Effect of a Nonsystemic, Orally Administered Hydrogel, GS100, on Metformin Pharmacokinetics. Can. J. Physiol. Pharmacol..

[432] Cuda S., Censani M., Kharofa R., O’Hara V., Conroy R., Williams D.R., Paisley J., Browne A.F., Karjoo S., Browne N.T. (2022). Medication-Induced Weight Gain and Advanced Therapies for the Child with Overweight and Obesity: An Obesity Medicine Association (OMA) Clinical Practice Statement 2022. Obes. Pillars.

[433] Singh S., Ricardo-Silgado M.L., Bielinski S.J., Acosta A. (2021). Pharmacogenomics of Medication-Induced Weight Gain and Antiobesity Medications. Obesity.

[434] Gill H., Gill B., El-Halabi S., Chen-Li D., Lipsitz O., Rosenblat J.D., Van Rheenen T.E., Rodrigues N.B., Mansur R.B., Majeed A. (2020). Antidepressant Medications and Weight Change: A Narrative Review. Obesity.

[435] Haslam D.W., James W.P.T. (2005). Obesity. Lancet.

[436] Ortega F.B., Lavie C.J., Blair S.N. (2016). Obesity and Cardiovascular Disease. Circ. Res..

[437] Severin R., Sabbahi A., Mahmoud A.M., Arena R., Phillips S.A. (2019). Precision Medicine in Weight Loss and Healthy Living. Prog. Cardiovasc. Dis..

[438] Caulfield T. (2015). Obesity Genes, Personalized Medicine, and Public Health Policy. Curr. Obes. Rep..

[439] (2022). World Obesity Atlas. https://www.worldobesity.org/resources/resource-library/world-obesity-atlas-2022.

[440] https://www.who.int/news-room/fact-sheets/detail/obesity-and-overweight.

[441] De Lorenzo A., Romano L., Di Renzo L., Di Lorenzo N., Cenname G., Gualtieri P. (2020). Obesity: A Preventable, Treatable, but Relapsing Disease. Nutrition.

[442] Schauer P.R., Bhatt D.L., Kirwan J.P., Wolski K., Aminian A., Brethauer S.A., Navaneethan S.D., Singh R.P., Pothier C.E., Nissen S.E. (2017). Bariatric Surgery versus Intensive Medical Therapy for Diabetes—5-Year Outcomes. N. Engl. J. Med..

[443] Peterli R., Wölnerhanssen B.K., Peters T., Vetter D., Kröll D., Borbély Y., Schultes B., Beglinger C., Drewe J., Schiesser M. (2018). Effect of Laparoscopic Sleeve Gastrectomy vs Laparoscopic Roux-En-Y Gastric Bypass on Weight Loss in Patients with Morbid Obesity. JAMA.

[444] Chen G., Zhang G.-X., Peng B.-Q., Cheng Z., Du X. (2021). Roux-En-Y Gastric Bypass versus Sleeve Gastrectomy plus Procedures for Treatment of Morbid Obesity: Systematic Review and Meta-Analysis. Obes. Surg..

[445] Eisenberg D., Shikora S.A., Aarts E., Aminian A., Angrisani L., Cohen R.V., de Luca M., Faria S.L., Goodpaster K.P.S., Haddad A. (2023). Publisher Correction: 2022 American Society of Metabolic and Bariatric Surgery (ASMBS) and International Federation for the Surgery of Obesity and Metabolic Disorders (IFSO) Indications for Metabolic and Bariatric Surgery. Obes. Surg..

[446] Ghiassi S., Morton J.M. (2020). Safety and Efficacy of Bariatric and Metabolic Surgery. Curr. Obes. Rep..

[447] Gomes-Rocha S.R., Costa-Pinho A.M., Pais-Neto C.C., de Araújo Pereira A., Nogueiro J.P.M., Carneiro S.P.R., Santos-Sousa H.M.T.F., Lima-da-Costa E.J., Bouça-Machado R., Preto J.R. (2022). Roux-En-Y Gastric Bypass vs Sleeve Gastrectomy in Super Obesity: A Systematic Review and Meta-Analysis. Obes. Surg..

[448] Nuzzo A., Czernichow S., Hertig A., Ledoux S., Poghosyan T., Quilliot D., Le Gall M., Bado A., Joly F. (2021). Prevention and Treatment of Nutritional Complications after Bariatric Surgery. Lancet Gastroenterol. Hepatol..

[449] Pinzariu A.C., Pasca S.A., Sindilar A., Drochioi C., Balan M., Oboroceanu T., Niculescu S., Crauciuc D.V., Crauciuc E.G., Luca A. (2017). Adipose Tissue Remodeling by Prolonged Administration of High Dose of Vitamin D3 in Rats Treated to Prevent Sarcopenia. Rev. Chim..

[450] Pinzariu A.C., Oboroceanu T., Eloae F.Z., Hristov I., Costan V.V., Labusca L., Cianga P., Verestiuc L., Hanganu B., Crauciuc D.V. (2018). Vitamin D as a Regulator of Adipocyte Differentiation Effects in Vivo and in Vitro. Rev. Chim..

[451] Gasmi A., Bjørklund G., Mujawdiya P.K., Semenova Y., Peana M., Dosa A., Piscopo S., Gasmi Benahmed A., Costea D.O. (2022). Micronutrients Deficiences in Patients after Bariatric Surgery. Eur. J. Nutr..

[452] Mingrone G., Panunzi S., De Gaetano A., Guidone C., Iaconelli A., Capristo E., Chamseddine G., Bornstein S.R., Rubino F. (2021). Metabolic Surgery versus Conventional Medical Therapy in Patients with Type 2 Diabetes: 10-Year Follow-up of an Open-Label, Single-Centre, Randomised Controlled Trial. Lancet.

[453] Niroomand M., Fotouhi A., Irannejad N., Hosseinpanah F. (2019). Does High-Dose Vitamin D Supplementation Impact Insulin Resistance and Risk of Development of Diabetes in Patients with Pre-Diabetes? A Double-Blind Randomized Clinical Trial. Diabetes Res. Clin. Pract..

[454] Tareque M.I., Saito Y., Chan A., Visaria A., Ma S., Malhotra R. (2019). Years of Life with and without Limitation in Physical Function and in Activities of Daily Living by Body Mass Index among Older Adults. Int. J. Obes..

[455] Rozjabek H., Fastenau J., LaPrade A., Sternbach N. (2020). Adult Obesity and Health-Related Quality of Life, Patient Activation, Work Productivity, and Weight Loss Behaviors in the United States. Diabetes Metab. Syndr. Obes..

[456] Buckell J., Mei X.W., Clarke P., Aveyard P., Jebb S.A. (2021). Weight Loss Interventions on Health-Related Quality of Life in Those with Moderate to Severe Obesity: Findings from an Individual Patient Data Meta-Analysis of Randomized Trials. Obes. Rev..

[457] Huang H., Yan Z., Chen Y., Liu F. (2016). A Social Contagious Model of the Obesity Epidemic. Sci. Rep..

[458] Blüher M. (2019). Obesity: Global Epidemiology and Pathogenesis. Nat. Rev. Endocrinol..

[459] Flint S.W., Snook J. (2015). Disability Discrimination and Obesity: The Big Questions?. Curr. Obes. Rep..

[460] Withrow D., Alter D.A. (2011). The Economic Burden of Obesity Worldwide: A Systematic Review of the Direct Costs of Obesity. Obes. Rev..

[461] Kracht C.L., Burkart S., Flanagan E.W., Melnick E., Luecking C., Neshteruk C. (2023). Policy, System, and Environmental Interventions Addressing Obesity and Diet-Related Outcomes in Early Childhood Education Settings: A Systematic Review. Obes. Rev..

[462] Wolfenden L., Barnes C., Jones J., Finch M., Wyse R.J., Kingsland M., Tzelepis F., Grady A., Hodder R.K., Booth D. (2020). Strategies to Improve the Implementation of Healthy Eating, Physical Activity and Obesity Prevention Policies, Practices or Programmes within Childcare Services. Cochrane Database Syst. Rev..

[463] Spencer L., Rollo M., Hauck Y., MacDonald-Wicks L., Wood L., Hutchesson M., Giglia R., Smith R., Collins C. (2015). The Effect of Weight Management Interventions That Include a Diet Component on Weight-Related Outcomes in Pregnant and Postpartum Women: A Systematic Review Protocol. JBI Database System. Rev. Implement. Rep..

